# Association of Immune and Inflammatory Gene Polymorphism With the Risk of IgA Nephropathy: A Systematic Review and Meta-Analysis of 45 Studies

**DOI:** 10.3389/fimmu.2021.683913

**Published:** 2021-06-30

**Authors:** Xiaonan Ding, Yan Mei, Zhi Mao, Lingling Long, Qiuxia Han, Yanqin You, Hanyu Zhu

**Affiliations:** ^1^ Medical School of Chinese PLA, Beijing, China; ^2^ Department of Nephrology, The First Medical Center, Chinese PLA General Hospital, Chinese PLA Institute of Nephrology, State Key Laboratory of Kidney Diseases, National Clinical Research Center for Kidney Diseases, Beijing, China; ^3^ Department of Critical Care Medicine, The First Medical Center, Chinese PLA General Hospital, Beijing, China; ^4^ Department of Obstetrics and Gynecology, The First Medical Center, Chinese PLA General Hospital, Beijing, China

**Keywords:** IgA nephropathy, susceptibility, inflammatory molecules, immune pathway, single-nucleotide polymorphism, meta-analysis

## Abstract

IgA nephropathy is the most prevalent primary glomerulonephritis worldwide, with identical immunopathological characteristics caused by multiple etiologies as well as influenced by geographical and ethnical factors. To elucidate the role of immunologic and inflammatory mechanisms in the susceptibility to IgA nephropathy, we explored single nucleotide polymorphisms of related molecules in the immune pathways. We searched the PubMed database for studies that involved all gene variants of molecules in the 20 immunologic and inflammatory pathways selected from the Kyoto Encyclopedia of Genes and Genomes database. The odds ratios with their corresponding 95% confidence intervals in six genetic models (allele model, dominant model, homozygote model, heterozygote model, overdominant model, and recessive model) were summarized using fixed or random effect models. Subgroup analysis was conducted based on different ethnicities with generalized odds ratios. Heterogeneity was evaluated using the Q and I^2^ tests. Begg’s funnel plot and Egger’s linear regression test were used to evaluating possible publication bias among the included studies, and sensitivity analysis was used to test the stability of the overall results. A total of 45 studies met our selection criteria and eight related genetic association studies were retrieved, including 320 single-nucleotide polymorphisms from 20 candidate pathways, ranging from 2000 to 2021. A total of 28,994 healthy people *versus* 20,600 IgA nephropathy patients were enrolled. Upon meta-analyzed results that *TGFB1* (rs1800469, rs1982073, rs1800471), *IL-1B* (rs1143627), *IL-18* (rs1946518), and *TLR1* (rs5743557) showed effect with or without ethnicity difference. And 10 variants presented stable and robust related to IgA nephropathy. This research showed that genetic variants are related to the immunologic and inflammatory effects of IgA nephropathy pathogenesis. The meta-analysis results supported the previous researches, and may help deepen the understanding of pathogenesis and explore new targets for IgA nephropathy-specific immunotherapy.

## Introduction

IgA nephropathy (IgAN) is the most prevalent primary glomerulonephritis in the world, and 30–40% of patients progress to the end-stage renal disease within 20–30 years after diagnosis, requiring kidney transplantation or renal replacement therapy (1). However, the recurrence of IgAN most likely occurs during kidney transplantation treatment, because the abnormalities in the immune system are still not corrected (2). The prevalence of IgAN shows great variations across the world; among patients undergoing renal biopsy, the incidence of IgAN is highest in Asian populations, occupying almost 45%; the prevalence of IgAN is moderate in European populations and is around 25%, and African populations show the lowest prevalence at just under 5% (3). IgAN also shows gender differences in prevalence: its prevalence in males is five times as that in females and male gender is a prognostic factor which indicates poor renal outcomes (4). Although the disease appears to be sporadic, there is an increasing number of reports showing familial clusters of IgAN. In addition, these familial cases have poorer renal outcomes and have fewer gender differences than sporadic cases (5). Gender as well as geographical factors hint at a possibility of genetic vulnerability. To date, the understanding of the pathogenic mechanisms of IgAN is still being explored but based on previous studies, immunologic regulation and inflammation contributes significantly to IgAN (6, 7). The characteristics of IgAN includes an IgA deposition in the glomerular mesangium area with complexes which activate mesangial cells to overproduce cytokines, chemokines, and complements (8). These mesangial-released inflammatory mediators cause local glomerular damage and may also aggravate podocytes and the renal tubular interstitium through humoral crosstalk (9). Recently, several susceptibility loci have been identified in different populations which involved in antigen processing and presentation, the mucosal defense system and the alternative complement pathway (10). Previous systematic review and meta-analysis usually focused on one gene and its single variant. However, the immunologic regulation is a huge network with interactions between various proteins which requires systematic studying of the immune pathways. The magnitude and significance of risk effects in genetic association studies is usually estimated by different genetic models with odds ratio (OR) and its 95% confidence interval (CI). However, the genetic contrasts are not independent and might cause confusion during interpretation of results and due to the use of inappropriate method without general justification. In reality, the true underlying genetic model is unknown and most studies have provided several potential models which may contribute to the loss of information resolution. The generalized linear odds ratio (OR_G_) analyzes the account of cases/healthy control pairs in the study to indicate the mutational load of disease susceptibility (11).

In past studies, the chosen of genetic models might not have been detailed enough to support results. In order to provide a strong support to existing evidence, we performed cluster analysis on six genetic models using SNPs in studies and calculated OR_G._ However, the large disparities in prevalence based on ethnicity that most studies have reported in cohorts of Asian populations has limited the overall review and meta-analysis of IgAN.

## Materials and Methods

### Search Strategy

To elucidate the role of the immunologic and inflammatory mechanisms in the susceptibility to IgA nephropathy, we selected 20 candidate pathways classified by the Kyoto Encyclopedia of Genes and Genomes (KEGG) database (**Supplementary Box 1**). In the meta-analysis, we searched the PubMed database for genetic association studies of all molecules in the aforementioned pathways involved in IgA nephropathy. The search strategy was based on the following combined MeSH terms: “IgA nephropathy”[Title/Abstract] AND (“genes”[MeSH Terms] OR “genes”[All Fields] OR “gene”[All Fields]) AND “related molecule name”[All Fields], (GWAS[Title]) AND (IgA nephropathy[Title]) (genome-wide association study[Title]) AND (IgA nephropathy[Title]). The eligible studies were all published in English and peer-reviewed, up to February 24, 2021, and we did not search gray literature (i.e., preprints, reports, or conference abstracts). Finally, references in the included articles were retrieved to identify further relevant studies.

### Selection Criteria

The following inclusion criteria were met for the meta-analysis: (1) the IgA nephropathy diagnosis was confirmed by renal biopsy; (2) healthy individuals included in the study as a control group; (3) sufficient genotypic data, such as genotype count data or allele frequencies, were provided; and (4) disease susceptibility was investigated.

Studies meeting any one of the following criteria were excluded: (1) the research subjects were patients after kidney transplantation or recurrent patients; (2) no healthy subjects as a control group; (3) studies investigating progression, prognosis, severity, response to treatment; (4) comments, case reports, editorials, letters, reviews, and non-English articles; (5) animal studies and *in vitro* experiments; and (6) data unextractable studies.

Two reviewers independently screened the abstracts of the retrieved articles, and if necessary, the full text was also reviewed. Discrepancies regarding potentially eligible studies were finalized through discussion to reach a consensus.

### Data Extraction

We used electronic data to extract the following information from each eligible study: first author, publication year, study country, PMID, ethnicity, disease type, sample size, age, and sex. For genotypic data, we extracted genotype count data in cases and controls, and, if available, the odds ratios (ORs) with their corresponding 95% confidence intervals (CIs) of five genetic models and evidence of Hardy-Weinberg equilibrium (HWE) in controls were also recorded.

### Quality Assessment

We appraised the quality and risk of bias of the included studies according to the Newcastle–Ottawa Quality Assessment Scale (NOS). The total score of NOS is 9 points, including three aspects: selection, comparability, and exposure. According to the final score, the studies could be categorized into high quality (score >6), medium quality (score between 4 and 6), and low quality (score less than 4).

### Statistical Analysis

STATA SE software (version 15.0) was used to conduct all the data processing. HWE was examined using Fisher’s exact test and whether controls were confronted with HWE according to P-value results, with 0.05 as the boundary. The association between the mutational load of a variant and susceptibility to IgAN was presented by ORs with 95% CIs. For each variant, the OR estimates were calculated using the fixed-effects model (Mantel-Haenszel method). Statistical significance was set at P < 0.05. If the heterogeneity test I^2^≥50% or p < 0.05 of the Q test, indicating that the homogeneity was insignificant, then the random-effects model (DerSimonian and Laird method) was considered. Since genetic models are not independent and there is no *a priori* biological justification for the choice, we performed genetic association analyses using six genetic models (allele model, dominant model, homozygote model, heterozygote model, overdominant model, and recessive model) for each molecule (12). Multiple genetic models could also reduce the probability of type I errors in genotype distribution in previous studies (13). Hence, OR_G_ was calculated using ORGGASMA (http://biomath.med.uth.gr) software that was programmed by statist Elias Zintzaras (11).

Additionally, when the heterogeneity between studies was statistically significant, we used meta-regression analysis to identify potential sources of such heterogeneity. Begg’s funnel plot and Egger’s linear regression test were used to evaluating possible publication bias among the included studies. The stability of the combined results was examined by performing a sensitivity analysis.

## Results

### Characteristics of Studies

**Figure 1:**
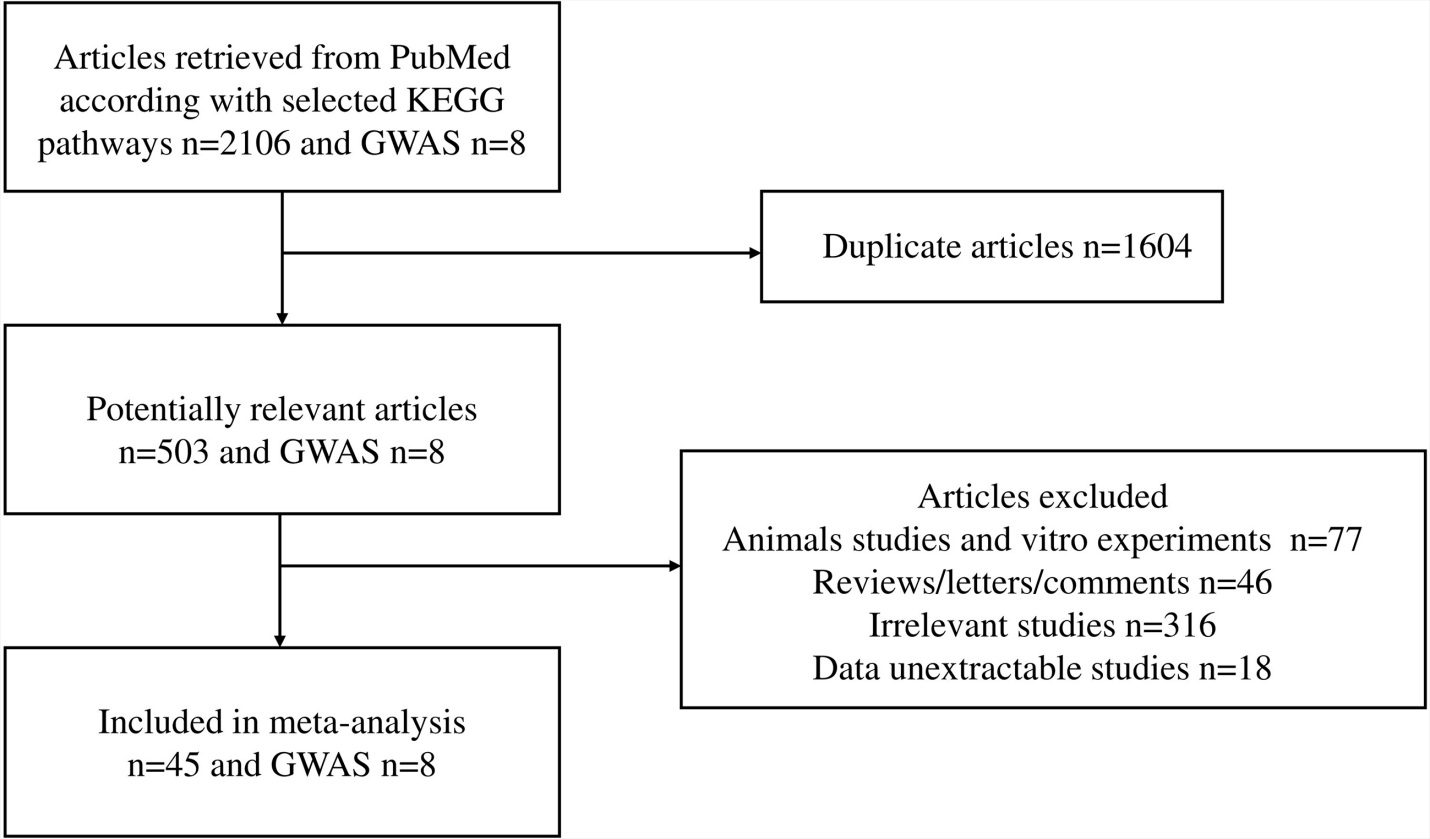
Flow chart of studies selected for meta-analysis.

The workflow of the literature selected for meta-analysis is shown in **Figure 1**. We obtained 45 studies, of which 8 studies involved Caucasians, and 37 studies recruited Asians (25 studies in Chinese, 1 study in Japanese, and 11 studies in Korean). Across these publications, nine studies included only pediatric patients, whereas most study subjects were adults. The participants ranged in age from 11.15 ± 5.11 to 48.1 ± 13.79, and 57.1% were male patients. Overall, 96 molecules from 20 candidate pathways and 320 polymorphisms were investigated in 45 studies published between 2000 and 2021. A total of 28,994 healthy people versus 20,600 patients had IgA nephropathy. The general characteristics of each included study and their references are shown in **Supplementary Table 1**. Among a total of 320 gene polymorphisms, 48 were not confronted with HWE.

### Meta-Analysis Results

#### Allele Model

**Figure 2:**
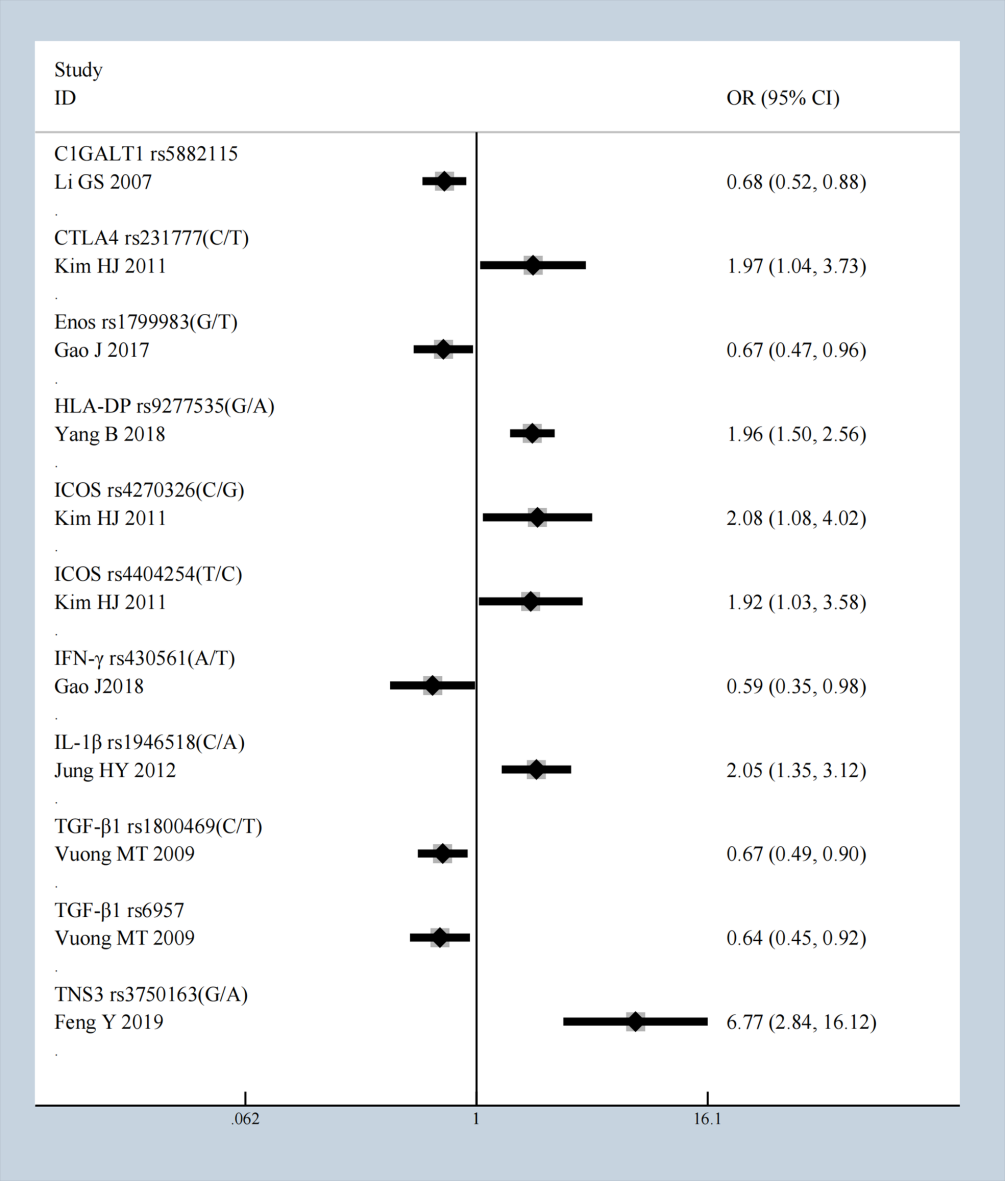
The most statistically significant SNPs under allele model.

We conducted a meta-analysis of all molecules with gene polymorphisms that were confronted with HWE. The results show significant association with susceptivity to IgAN under the allele contrast model, as follows: *core 1 synthase, glycoprotein-n-acetylgalactosamine 3-beta-galactosyltransferase 1 (C1GALT1), complement factor B (CFB), Cytotoxic T-Lymphocyte Associated Protein 4 (CTLA4), C-X-C Motif Chemokine Ligand 8 (CXCL8), endothelial nitric oxide synthase (Enos), Fc Receptor Like A (FCRLA), FCRLB, G Protein Subunit Gamma 2 (GNG2), Major Histocompatibility Complex, Class II, DP (HLA-DP), Inducible T Cell Costimulator (ICOS), Interferon-γ (IFN-γ), Interleukin 10 (IL-10), IL-1B, Interleukin 1 Receptor Antagonist (IL1RN), IL-6, Integrin Subunit Alpha X (ITGAX), LEM Domain Nuclear Envelope Protein 2 (LEMD2), Netrin 4 (NTN4), Pleckstrin Homology Like Domain Family B Member 1 (PHLDB1), Signal Transducer And Activator Of Transcription 4 (STAT4), Transforming Growth Factor-β (TGF‐β1), Toll Like Receptor 1 (TLR1), TLR10, Tumor Necrosis Factor Superfamily Member 13 (TNFSF13), Tensin 3 (TNS3)*, and *Vascular Endothelial Growth Factor (VEGF)*; the details are shown in **Supplementary Table 2**. The studies with the most statistically significant results, and the detailed information on per-single-nucleotide polymorphisms (SNPs) of significant OR with 95% CIs under the allele model are presented in **Table 1** and **Figure 2**.

**Table 1:** SNPsof most significant OR with 95% CIs under the allele model.

author	year	PMID	SNP	Ethnicity	Allele model OR	0.95LCI	0.95UCI	P
Li GS (14)	2007	17228361	C1GALT1 rs5882115	Han Chinese	0.679	0.522	0.884	0.004
Kim HJ (15)	2011	21677403	CTLA4 rs231777	Korean pediatric patients	1.973	1.045	3.725	0.036
Gao J (16)	2017	28946141	Enos rs1799983	Han Chinese	0.671	0.469	0.959	0.029
Yang B (17)	2018	29467950	HLA-DP rs9277535	Han Chinese	1.958	1.497	2.560	0
Kim HJ (15)	2011	21677403	ICOS rs4270326	Korean pediatric patients	2.082	1.079	4.018	0.029
Kim HJ (15)	2011	21677403	ICOS rs4404254	Korean pediatric patients	1.919	1.029	3.579	0.04
Gao J (18)	2018	28391282	IFN-γ rs430561	Han Chinese	0.589	0.353	0.982	0.042
Jung HY (19)	2012	26889427	IL-1β rs1946518	Korean	2.055	1.353	3.120	0.001
Vuong MT (20)	2009	19258388	TGF‐β1 rs1800469	Sweden	0.666	0.494	0.898	0.008
Vuong MT (20)	2009	19258388	TGF‐β1 rs6957	Sweden	0.644	0.449	0.924	0.017
Feng Y (21)	2019	30928649	TNS3 rs3750163	Han Chinese	6.768	2.841	16.123	0

**Figure 3:**
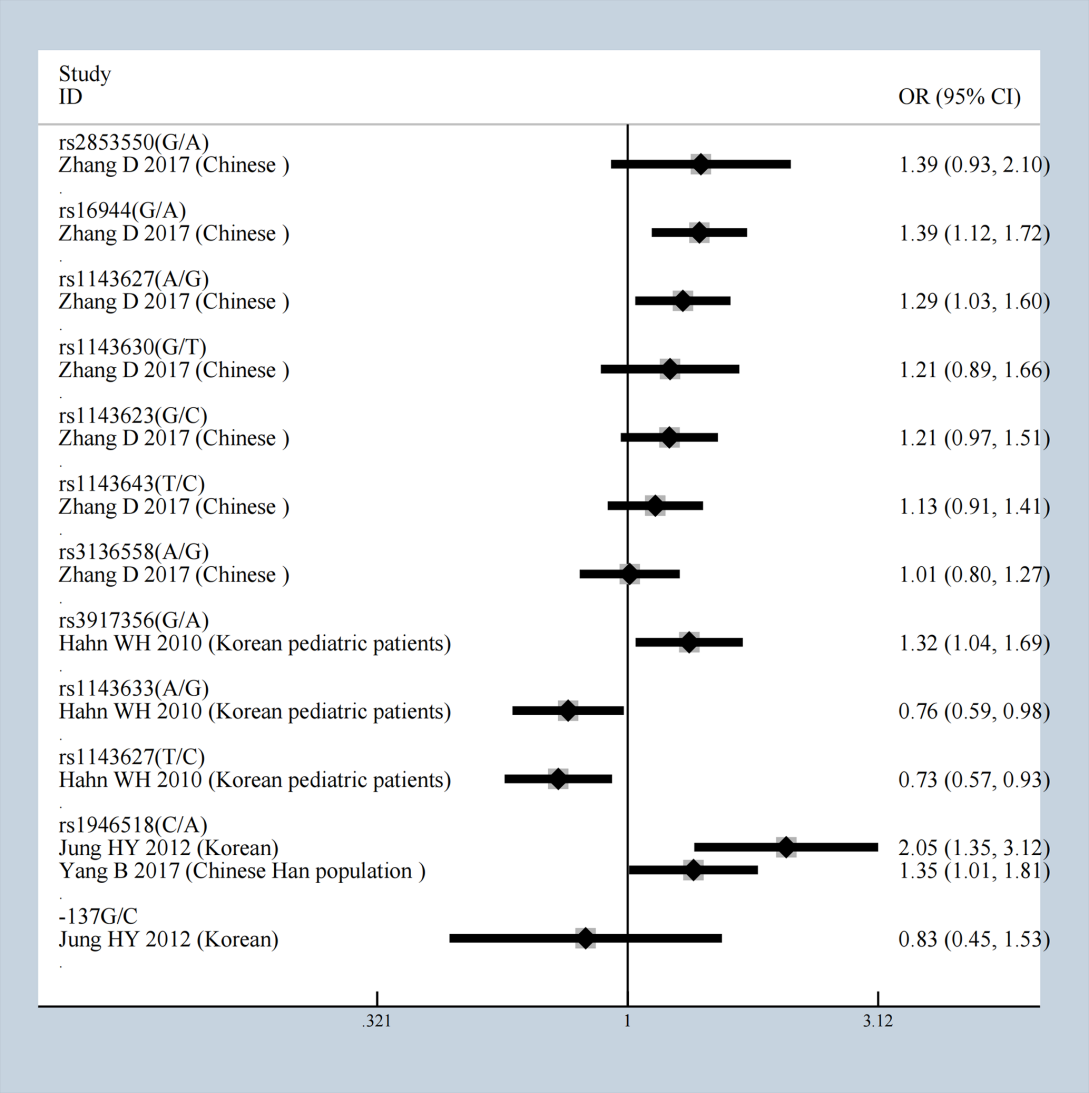
The results of 13 gene variants of IL-1b under allele model.

It is worth mentioning that the five gene variants of *FCRLB* (rs12079477, rs1891019, rs1891020, rs1417582, and rs4657093) have all been shown to have a statistical impact on IgAN susceptibility in the allele model **Supplementary Figure 1** (22). The meta-analysis results of 13 gene variants of IL-1β are shown in **Figure 3**, in which rs1946518 showed an increase in the IgAN susceptibility both in the Chinese Han population and Korean. *CTLA4* has three SNPs (rs231777, rs5742909, and rs231779), all of which were significant. Both rs9277535 and rs3077 in HLA-DP showed an increased risk of developing IgAN. In addition, *ICOS* has eight gene variants, five of which have protective effects under the allele model (rs4270326, rs4404254, rs10183087, rs11571314, rs1559931) were associated with susceptibility to IgA in **Supplementary Figure 2**.

#### Dominant Model

To further validate the association of these significant SNPs with the risk of IgAN under allele model, we analyzed all gene polymorphisms in dominant contrast model. The genes significantly associated with IgAN occurrence and compounded with HWE were as follows: *C1GALT1, CFB, CTLA4, CXCL8, FCRLA, FCRLB, HLA-DP, ICOS, IFN-γ, IL-10, IL-1β, IL1RN, IL4R, IL5RA, IL-6, ITGAM, ITGAX, Netrin 4 (NTN4), ST6GAL1, ST6GALNAC2, TGF‐β1, TLR1, TLR10, TNFRSF6B, TNFSF13*, and *TNS3.* The general characteristics and details of each research on significant SNPs are described in **Supplementary Table 3**. We identified ORs higher than 2 and lower than 0.5 as the most significant and the results are presented in **Table 2**. Then, we noticed that *ICOS* has five SNPs (rs4270326, rs4404254, rs10183087, rs11571314, and rs1559931), all of which showed a robust significant risk of developing IgAN under dominant model which corresponded with the allele model results. Besides that, *TNFRSF6B* with the mutations of rs1291205 [OR = 0.45 (95% CI 0.31–0.54)], rs1291206 [OR = 0.46 (95% CI 0.31–0.67)], rs3208008 [OR = 0.47 (95% CI 0.32–0.69)] showed significant protective effect. And we obtained interesting results that did not show significance in allele model. The SNPs rs12054151, rs2239611, rs1990677, rs4686838, rs2284750, rs6784233, and rs7634389 in *ST6GAL1* showed robust association with the occurrence of IgAN; rs3840858 in *ST6GALNAC2* [OR = 3.68 95% CI (1.75–7.73)] showed a great correlation with IgAN risk in Chinese Uyghur population.

**Table 2:** The most significant SNPs with ORs under dominant model higher than 2 and lower than 0.5.

Author	Year	PMID	Ethnicity	SNP	Dominant model OR	0.95LCI	0.95UCI	P
Yang B (17)	2018	29467950	Han Chinese	HLA-DP rs9277535	2.05354	1.34	3.158399	0.001
Kim HJ (15)	2011	21677403	Korean pediatric patients	ICOS rs4270326	2.23918	1.08	4.632189	0.03
Kim HJ (15)	2011	21677403	Korean pediatric patients	ICOS rs4404254	2.21088	1.09	4.467172	0.027
Kim HJ (15)	2011	21677403	Korean pediatric patients	ICOS rs10183087	2.17830	1.09	4.347564	0.027
Kim HJ (15)	2011	21677403	Korean pediatric patients	ICOS rs11571314	2.17830	1.09	4.347564	0.027
Kim HJ (15)	2011	21677403	Korean pediatric patients	ICOS rs1559931	2.17830	1.09	4.347564	0.027
Liu XQ (23)	2008	18256355	Caucasians (St. Etienne)	IL4R rs1805015	0.43366	0.28	0.664227	0
Fu D (24)	2020	32747022	Han Chinese	ST6GAL1 rs4686838	0.36516	0.30	0.443134	0
Lu C (25)	2015	26136946	Uyghur Chinse	ST6GALNAC2 rs3840858	3.67568	1.75	7.73074	0.001
Vuong MT (20)	2009	19258388	Sweden(male)	TGF‐β1 rs6957	0.14469	0.04	0.542827	0.004
Vuong MT (20)	2009	19258388	Sweden(male)	TGF‐β1 rs180047	0.04851	0.00	0.907181	0.043
Liu XQ (23)	2008	18256355	Caucasians (St. Etienne)	TNFRSF6B rs3208008	0.46880	0.32	0.684625	0
Liu XQ (23)	2008	18256355	Caucasians (St. Etienne)	TNFRSF6B rs1291206	0.45731	0.31	0.667693	0
Liu XQ (23)	2008	18256355	Caucasians (St. Etienne)	TNFRSF6B rs1291205	0.44846	0.31	0.654722	0

#### Overdominance Model

**Figure 4:**
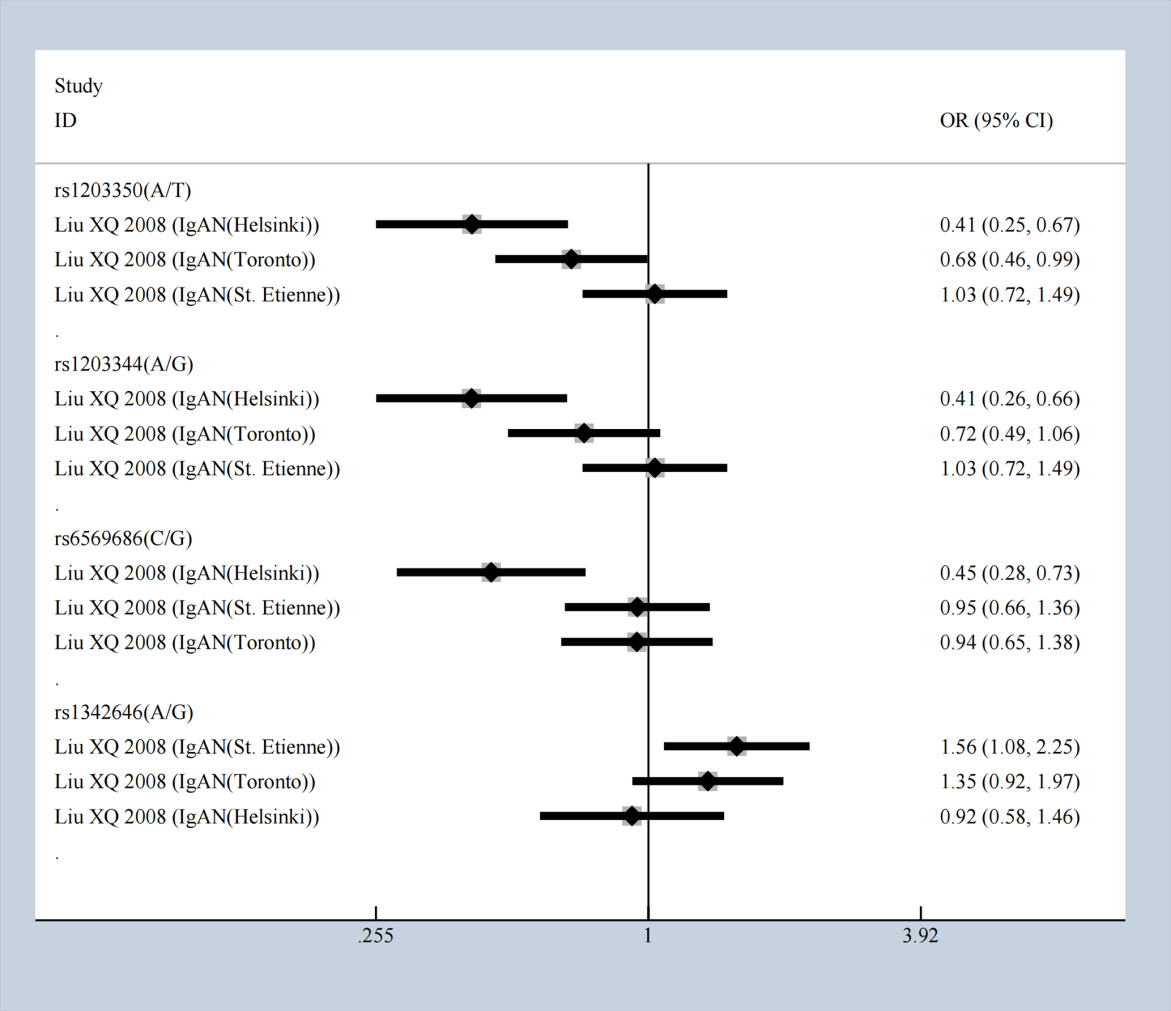
The SNPs of IGAN1 in different population under overdominance model.

The SNPs rs3840858 and rs23840858 in *ST6GALNAC2* showed protective effect respectively with [OR = 0.27 (95% CI 0.13–0.57)] and [OR = 0.64 (95% CI 0.41–0.99)] under the overdominance model which contradicted with dominance model result, but still hinted at IgAN occurrence in Uyghur Chinese population. The five SNPs of *ICOS* mentioned above were stably and significantly associated with IgAN but were associated with protective effects under overdominance model. Our meta-analysis results showed that rs1203350 and rs1203344 in *IGAN1* decrease the susceptibility of IgAN in Helsinki and Toronto population, but not in St. Etienne patients. Besides that, according to the results of overdominance model, rs6569686 decreases the susceptibility of IgAN in Helsinki people (**Figure 4**). The following showed a significant association including *C1GALT1, CFB, CXCL8, FCRLA, FCRLB, HLA-DP, ICOS, IFN-γ, IL-10, IL-1β, IL4R, ITGAM, ITGAX, ST6GAL1, ST6GALNAC2, TGF‐β1. TNFRSF6B, TNS3, IGAN1*, *IL22RA1*, and *STAT4* emerged with significant statistic meaning from multitudinous immune molecules under overdominance model (**Supplementary Table 4**) and most significant results were presented in **Table 3**.

**Table 3:** SNPs of most significant OR with 95% CIs under the overdominant model.

Author	Year	Ethnicity	PMID	SNP	Overdominant OR	0.95-LCI	0.95-UCI	P
Yang B (17)	2018	Han Chinese	29467950	HLA-DP rs3077	4.62	2.809256	7.588952	0
Liu XQ (23)	2008	Caucasians (St. Etienne)	18256355	IL4R rs1805015	2.45	1.58403	3.788273	0
Yang B (17)	2018	Han Chinese	29467950	STAT4 rs7574865	2.38	1.553347	3.638879	0
Fu D (24)	2020	Han Chinese	32747022	ST6GAL1 rs4686838	1.85	1.490156	2.274617	0
Liu XQ (23)	2008	Caucasians (St. Etienne)	18256355	TNFRSF6B rs1291205	1.78	1.209218	2.614096	0.003
Gao J (18)	2018	Han Chinese (St. Etienne)	28391282	IFN-γ rs430561	1.75	1.033877	2.949718	0.037
Liu XQ (23)	2008	Caucasians	18256355	TNFRSF6B rs1291206	1.74	1.184463	2.562628	0.005
Liu XQ (23)	2008	Caucasians (St. Etienne)	18256355	TNFRSF6B rs3208008	1.70	1.154023	2.499533	0.007
Fu D (24)	2020	Han Chinese	32747022	ST6GAL1 rs2284750	1.58	1.274685	1.964356	0
Liu XQ (23)	2008	Caucasians (St. Etienne)	18256355	IGAN1 rs1342646	1.56	1.080724	2.245152	0.017
Yang B (26)	2017	Han Chinese	27028244	IL-1β rs1946518	1.53	1.00525	2.338833	0.047
Hahn WH (27)	2010	Korean pediatric patients	19280228	IL-1β rs1143633	1.50	1.060556	2.127999	0.022
Kim HJ (15)	2011	Korean pediatric patients	21677403	ICOS rs4270326	0.47	0.22301	0.992154	0.048
Kim HJ (15)	2011	Korean pediatric patients	21677403	ICOS rs10183087	0.45	0.221188	0.92188	0.029
Kim HJ (15)	2011	Korean pediatric patients	21677403	ICOS rs11571314	0.45	0.221188	0.92188	0.029
Kim HJ (15)	2011	Korean pediatric patients	21677403	ICOS rs1559931	0.45	0.221188	0.92188	0.029
Kim HJ (15)	2011	Korean pediatric patients	21677403	ICOS rs4404254	0.44	0.214075	0.91871	0.029
Lu C (25)	2015	Uyghur Chinese	26136946	ST6GALNAC2 rs3840858	0.27	0.129354	0.572199	0.001
Feng Y (21)	2019	Han Chinese	30928649	TNS3 rs3750163	0.06	0.013265	0.23499	0

#### Homozygote Model

It is generally recognized that homozygote of the mutation increases the risk for disease than the heterozygote, whereas homozygotes of the wild allele are not risky (11). *C1GALT1* rs5882115 showed a significant effect under homozygote model [OR = 0.70 95% CI (0.55–0.91], allele model [OR = 0.68 95% CI (0.52–0.89)], dominant model [OR = 0.68 95% CI (0.51–0.90)], and overdominant model [OR = 1.39 95% CI (1.04–1.85)]. Several SNPs showed significance only in homozygote model compared to the other genetic models mentioned above including *Activating Transcription Factor 6 (ATF6), Bone Morphogenetic Proteins 2* (*BMP2), C Motif Chemokine Receptor 6 (CCR6), F-Box And Leucine Rich Repeat Protein 21 (FBXL21), LEM Domain Nuclear Envelope Protein 2 (LEMD2), Pecanex 3 (PCNXL3), RAS Guanyl Releasing Protein 1(RASGRP1)*,and *Regulator Of G Protein Signaling 1 (RGS1).* The details of the homozygote model with p<0.05 and HWE≥ 0.05 are shown in **Supplementary Table 5**. Based on the homozygote model, compared to healthy controls, the OR value of IgA nephropathy was greater than 1.5, or less than 0.5 as shown in **Supplementary Figure 3**. Twelve variants of *IL-1B* under homozygote model are shown in **Supplementary Figure 4**, of which rs1946518 has a similar effect as that suggested by the allele model in Korean and Chinese Han population that increase the susceptibility of IgAN.

#### Heterozygote Model

**Figure 5:**
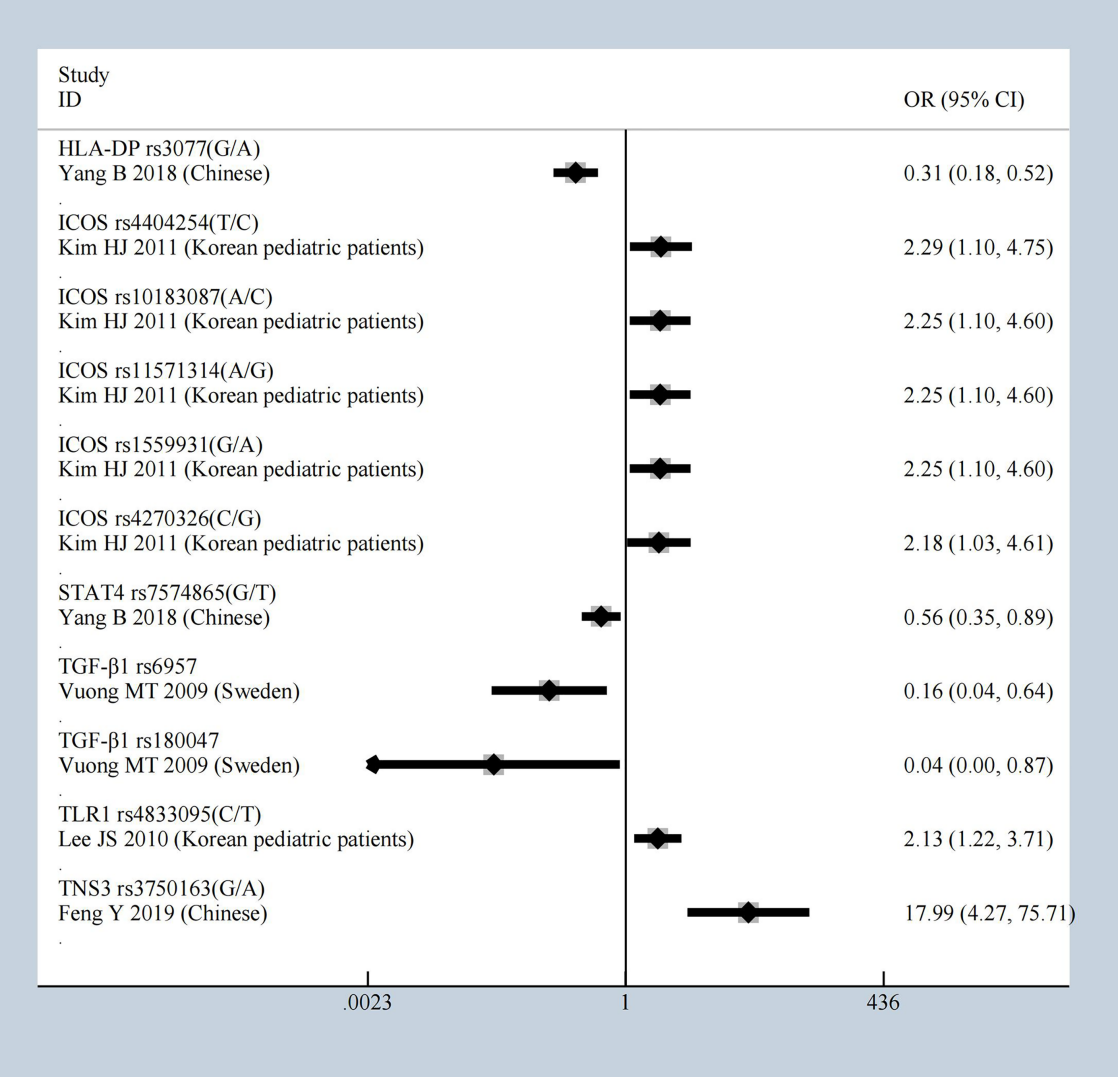
The most statistically significant SNPs under Heterozygote model.

Under heterozygote model, compared to healthy controls, ICOS showed significant effect again with the mutations of rs4404254, rs10183087, rs11571314, rs1559931, rs4270326, and *HLA-DP* rs3077 [OR = 0.31 (95% CI 0.18–0.52)], *STAT4* rs7574865 [OR = 0.56 (95% CI 0.35–0.89)], *TGF‐β1* rs6957 [OR = 0.16 (95% CI 0.04–0.64)] and *TGF‐β1* rs180047 [OR = 0.04 (95% CI 0–0.87)], *TLR1* rs4833095 [OR = 2.13 (95% CI 1.22–3.71)], *TNS3* rs3750163 [OR = 17.99 (95% CI 4.27–75.71)]; all had a significant effect which was identified as having an OR greater than 1.5 or less than 0.5, the results shown in **Figure 5**. All results of the heterozygote model (p < 0.05 and HWE ≥ 0.05) were: *CFB, C1GALT1*, and *CCR6, CD29, CTLA4, CXCL8, F-Box and Leucine Rich Repeat Protein 21 (FBXL21), Fc Fragment Of IgG Receptor IIb (FCGR2B), FCRLA, FCRLB, HLA-DP, ICOS, IFN-γ, IL-10, IL-1β, ITGAM, ITGAX, RGS1, STAT4, TGF‐β1, TLR1, TLR10, TNFSF13*, and *TNS3.* General characteristics of each studies about molecules are described in **Supplementary Table 6**.

### Recessive Model and Cluster Analysis

**Figure 6:**
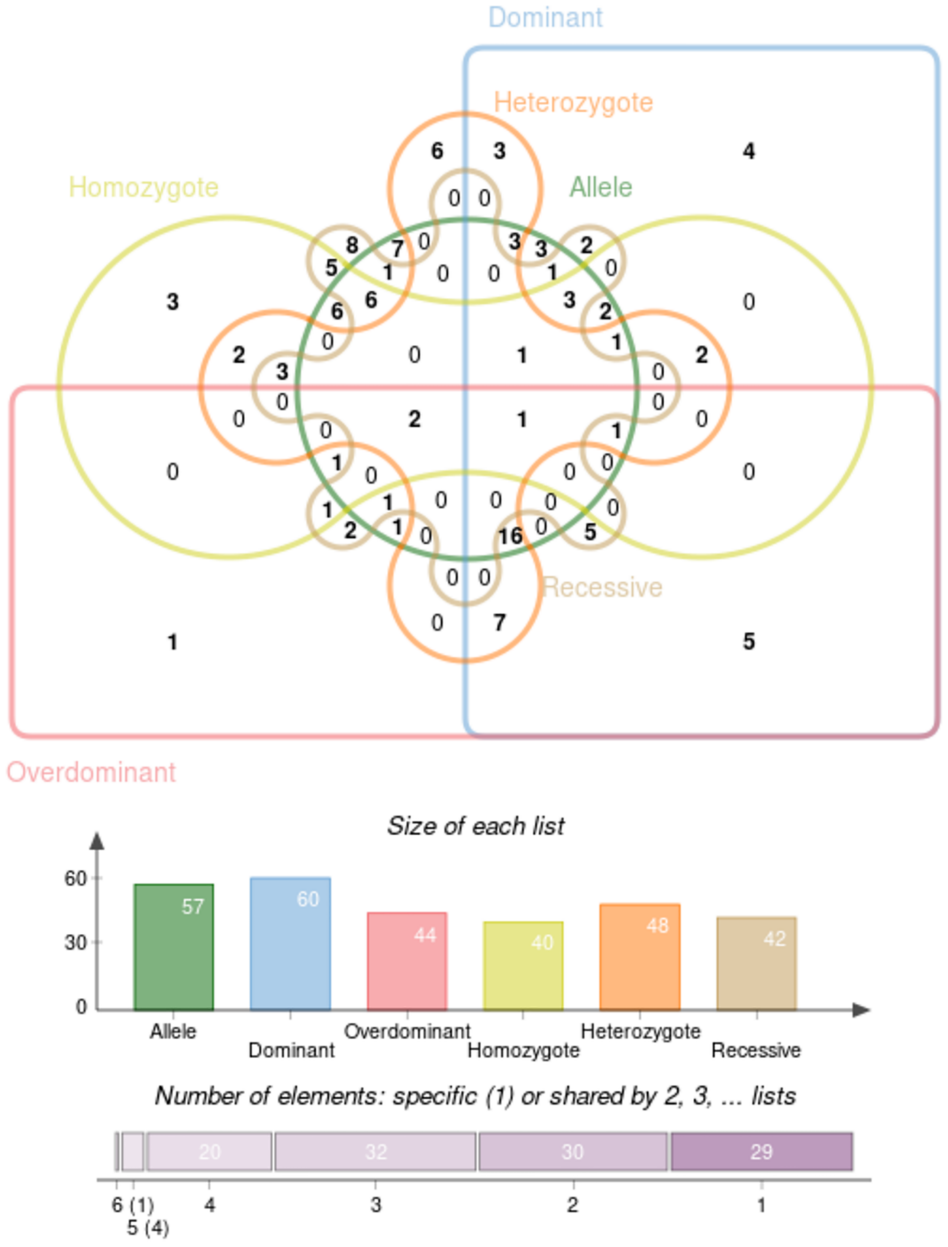
The cluster analysis of the SNPs with significant ORs under the six genetic models.

All results of recessive model were described in **Supplementary Table 7**. In order to find the SNPs which are stable and contribute to the predisposition of IgAN, we clustered the molecules with significant ORs under the six genetic models above, and the result was represented as a Venn diagram in **Figure 6**. And five SNPs (*IL-10* rs1800872, *CXCL8* rs2227543, *CXCL8* rs2227306, *ITGAM* rs4597342, and *TGF‐β1* rs2241715) have a significant effect in dominant model, overdominant model, and heterozygote model. Among alleles, *FCRLA* rs1954174, *IL1RN* rs928940, *IL1RN* rs315951, *LEMD2* rs751728, and *VEGF* 405C-G showed significance in homozygote and recessive models. Furthermore*, HLA-DP* rs3077 and *STAT4* rs7574865 showed statistical significance in allele model, overdominant model, homozygote model, heterozygote model, and recessive model. In addition, *TNFSF13* rs3803800 showed the most stable effect under five genetic models excepted the overdominant model; *CFB* rs549182 has a robust impact on susceptibility in all models except the homozygote one; there were two gene variants of *CFB* (rs549182 and rs4151657) that were significant in different patient groups with IgA nephropathy and we summarized it with OR_G_ in **Table 4**.

**Table 4:** Summary of the pooled ORs of two variants of *CFB* in patients with IgAN stage I or stage II.

	Allele model		Overdominant model		Dominant model		Heterozygote model		Homozygote model		Recessive model		OR_G_ (95% CI)
	OR (95% CI)	P_H_	OR (95% CI)	P_H_	OR (95% CI)	P_H_	OR (95% CI)	P_H_	OR (95% CI)	P_H_	OR (95% CI)	P_H_	
rs549182													
IgAN Stage I	1.33 (1.10,1.61)	0.003	0.88 (0.66,0.99)	0.040	1.30 (1.07,1.60)	0.010	1.25 (1.02,1.53)	0.032	4.03 (1.34,12.19)	0.013	3.90 (1.29,11.77)	0.016	1.31 (1.07,1.60)
IgAN stage II	1.19 (1.02,1.40)	0.031	1.0 (0.73,1.39)	0.979	0.94 (0.69,1.29)	0.716	0.99 (0.71,1.36)	0.937	0.41 (0.11,1.59)	0.197	0.41 (0.11,1.59)	0.197	0.94 (0.68,1.28)
IgAN Stage I and stage II	0.91 (0.68,1.22)	0.513	0.86 (0.72,1.02)	0.085	1.19 (1.00,1.41)	0.050	1.17 (0.98,1.39)	0.077	1.70 (0.80,3.61)	0.167	1.66 (0.78,3.52)	0.187	1.19 (1.01,1.41)
rs4151657													
IgAN Stage I	1.13 (1.01,1.27)	0.031	0.80 (0.69,0.93)	0.003	1.25 (1.07,1.44)	0.004	1.28 (1.09,1.49)	0.002	1.11 (0.86,1.44)	0.417	0.99 (0.77,1.27)	0.928	1.18 (1.04,1.35)
IgAN stage II	1.21 (1.02,1.45)	0.033	0.77 (0.61,0.97)	0.029	1.35 (1.07,1.70)	0.012	1.36 (1.06,1.73)	0.014	1.30 (0.86,1.98)	0.217	1.12 (0.75,1.67)	0.584	1.28 (1.04,1.58)
IgAN Stage I and stage II	1.15 (1.05,1.27)	0.003	0.79 (0.70,0.90)	0.000	1.27 (1.12,1.44)	0.000	1.30 (1.14,1.48)	0.000	1.16 (0.93,1.45)	0.181	1.02 (0.83,1.26)	0.839	1.21 (1.08,1.35)

### Genetic Association Studies

To date, eight GWASs have been performed that provide valuable information on the susceptibility of IgAN. **Table 5** shows the information and significant molecules of the eight GWAS studies. Because none of them provided extractable data, we used their conclusions to prove our meta-analysis results. *C1GALT1*, *HLA-DP*, *ITGAX*, *ITGAM*, *TNFSF13*, and *ST6GAL1* showed significant associations according to our meta-analysis results, further confirmed by GWAS studies. Recently, a large-scale genome-wide meta-analysis included four GWAS studies on people of Chinese and European descent to investigate genetic variants of IgAN susceptibility (34). This analysis revealed novel SNPs as susceptibility genes for IgAN: *FCRL3* rs6427389, *Dual Specificity Phosphatase 22 (DUSP22), interferon regulatory factor 4 (IRF4)* rs6942325, and *Peptidyl Arginine Deiminase 4 (PADI4)* rs2240335.

**Table 5:** The general characteristics and relevant molecules of genetic association studies.

Number	Author	Year	PMID	Ethnicity	Case	Control	Molecule
1	John Feehally (28)	2010	20595679	British	431	4,980	HLA-A, HLA-B, HLA-C, DRB, DQA, DRB (9)
2	Xue-Qing Yu (29)	2011	22197929	Chinese	4,137	7,734	TNFSF13, DEFA, MHC, HLA-A, HLA-DR-DQ (10)
3	Krzysztof Kiryluk (10)	2014	25305756	European	2,747	3,952	VAV3, CARD9, ITGAM-ITGAX, HLA-DQ/DR, DEFA, TAP1/PSMB8, HLA-DP, CFHR3, TNFSF13, HORMAD2 (11)
				East Asian			
4	Krzysztof Kiryluk (30)	2012	22737082	Asian, European, African-American	4,789	10,755	HLA-DQB1/DRB1, PSMB9/TAP1, DPA1/DPB2, CFHR3/R1, HORMAD2 (12)
5	Ali G Gharavi (31)	2011	21399633	Chinese	3,144	2,852	CFHR1, CFHR3, HLA-DQB1, DQA1, DRB1, PSMB8, TAP2, TAP1, PSMB9, HLA-DPA1, -DPB1, -DPB2 (13)
				European			
6	Sanae Saka (32)	2015	26202575	Japanese	915	481	HLA-DRA, HLA-DRB1, HLA-DQA1, HLA-DQB1, TSPAN8 and PTPRR (22)
7	Yan-Na Wang (33)	2021	33593824	Chinese	2,352	2,632	GALNT12, C1GALT1 (34)
8	Ming Li (35)	2015	26028593	Chinese	8,313	19,680	ST6GAL1, ACCS, ODF1-KLF10, ITGAX-ITGAM, DEFA (20)

### Ethnicity Differences Analysis

**Figure 7:**
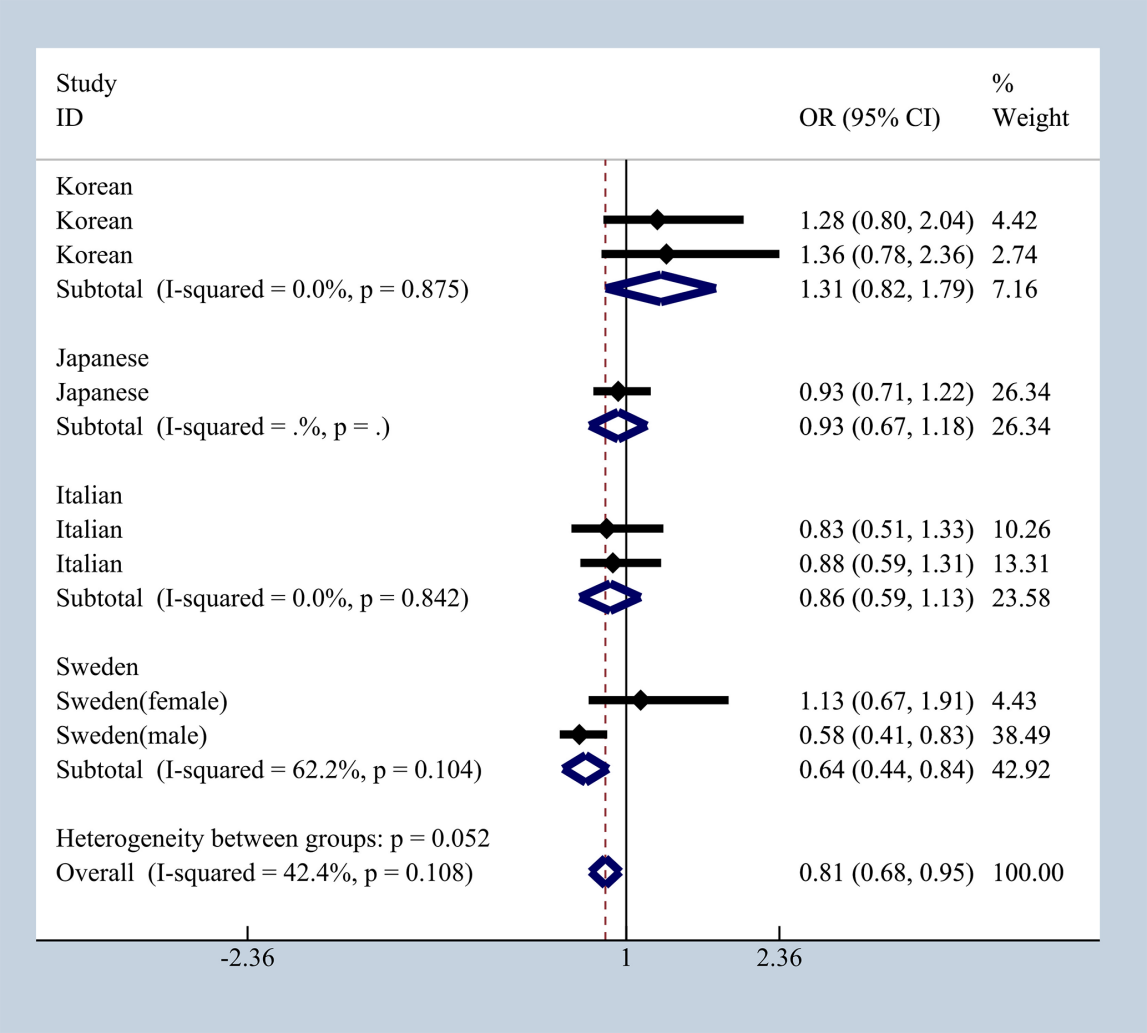
The different effects in four different populations of rs1800469 in TGFB1.

**Figure 8:**
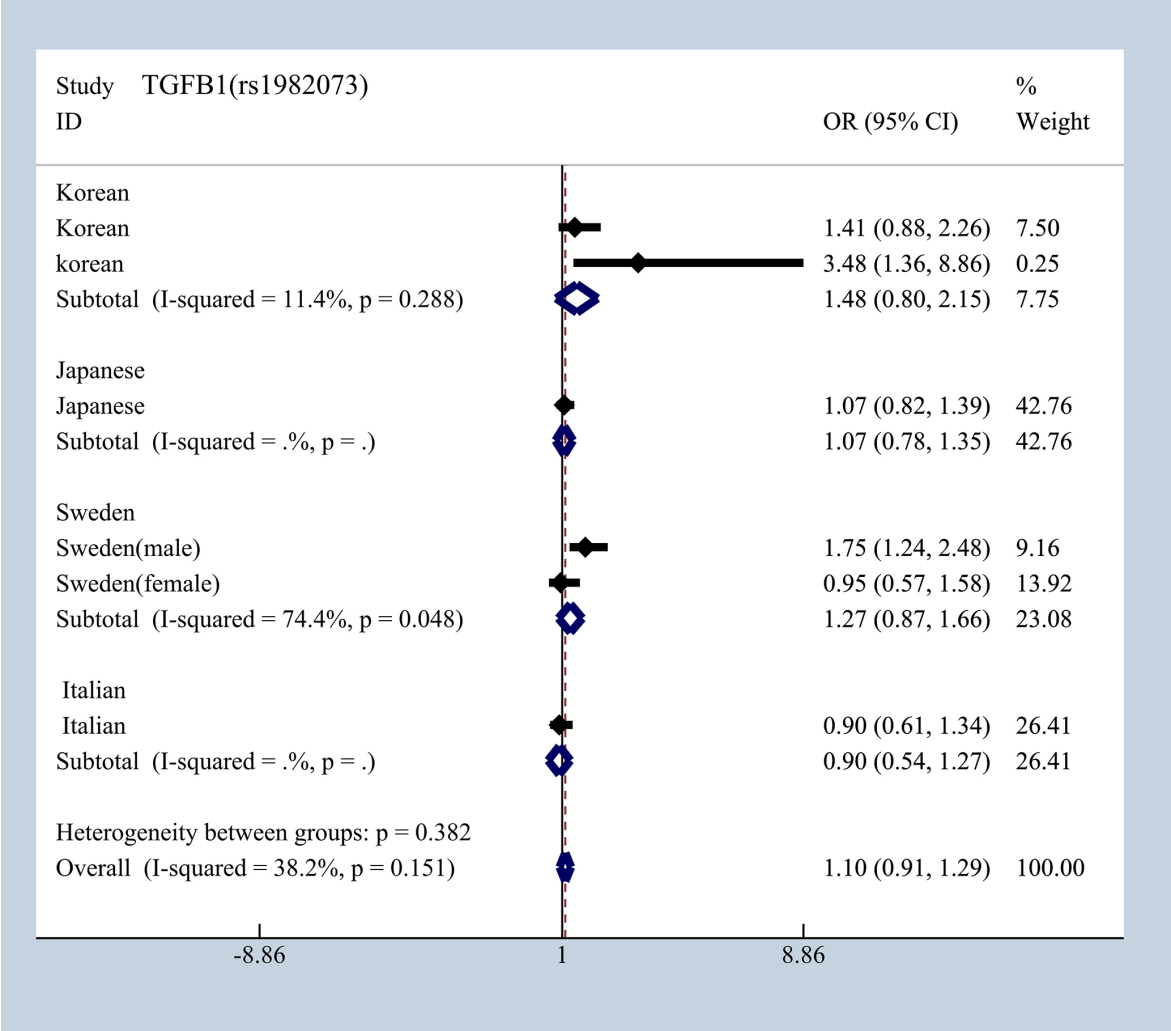
The different effects in four different populations of rs1982073 in TGFB1.

**Figure 9:**
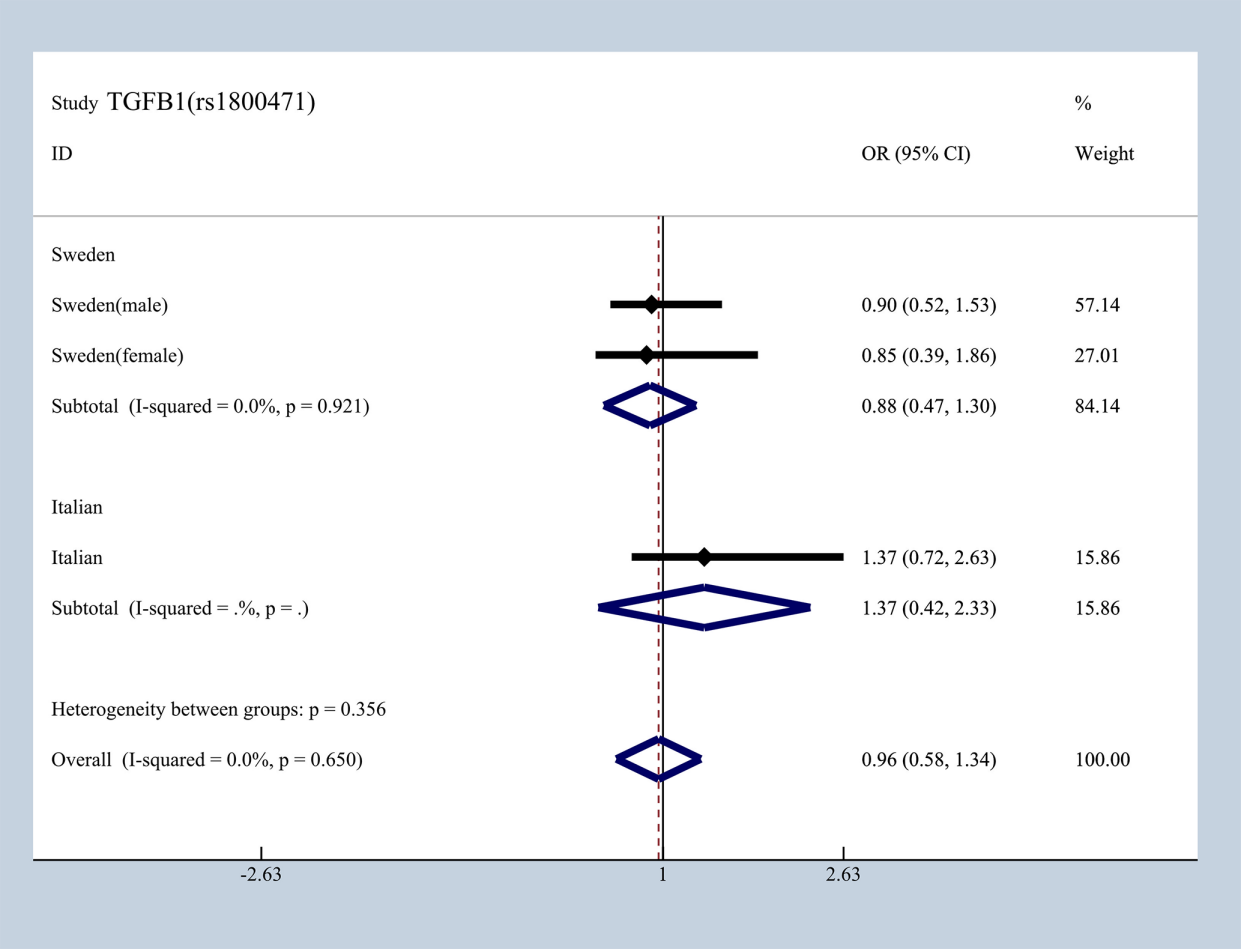
Different effects between Sweden and Italian in rs1800471of TGFB1.

To elucidate the effect of different ethnicities on SNPs of immunologic process in IgAN, we investigated all available data to conduct a meta-analysis based on OR_G_ and 95% CI. We summarized published studies related to rs1800469, rs1982073, and rs1800471 in TGFB1. Excluding those that did not meet our selection criteria, we finally enrolled five studies involving Korean, Japanese, Italian, and Swedish populations (19, 20, 36–39). In subgroup analyses by ethnicity and gender that we found in Japanese, Italian, and Sweden male group, we found that rs1800469 in TGFB1 has a moderate protective effect, whereas contradicting results was observed in Korean and Swedish female group with overall OR = 1.10, 95% CI 0.91–1.29, I^2^ = 38.2% (**Figure 7**). The mutation in SNP 1982073 in TGFB1 presented a slightly increased susceptibility of IgAN among Korean, Japanese, and Swedish male group and showed a decreased possibility risk of disease (**Figure 8**). The mutation in SNP rs1800471 has a similar moderate protective effect in Swedish population, both in males and females but showed an increased occurrence in Italian group (**Figure 9**) (20, 38).

**Figure 10:**
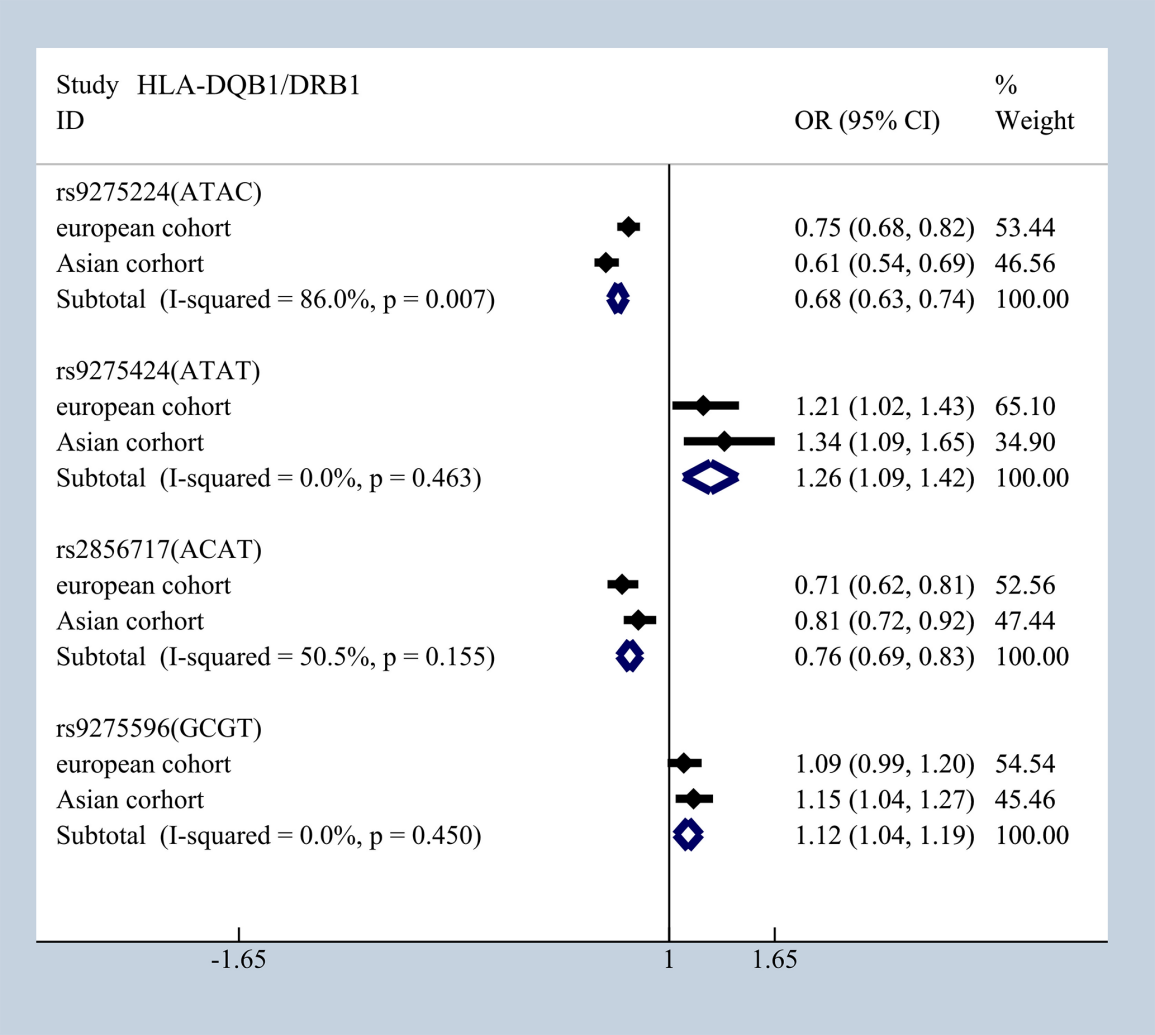
Consistent results in European and Asian cohorts in mutations of HLA-DQB1/DRB1.

Furthermore, based on available data, interleukin family proteins were investigated in Korean and Chinese Han population that had an IL-18 (rs1946518) mutation; it showed a significant protective effect with overall OR_G_ = 0.56, 95% CI (0.39–0.73) (19, 26) (**Supplementary Figure 5**). However, the contradictory result was observed in Chinese Han population and Korean pediatric patients in IL-1B (rs1143627) (40, 41) (**Supplementary Figure 6**). Chinese population and Korean pediatric patients shared the same predisposition of rs4833095 and rs5743557 in TLR1which presented in (**Supplementary Figure 7**) (42, 43). Besides that, mutations of HLA-DQB1/DRB1, rs9275224 [OR_G_ = 0.68 95% CI (0.63–0.74)], rs9275424 [OR_G_ = 1.26 95% CI (1.09–1.42)]; rs2856717 [OR_G_ = 0.76 95% CI (0.69–0.83)], and rs9275596 [OR_G_ = 1.12 95% CI (1.04–1.19)] had consistent significant results in European and Asian cohorts (**Figure 10**).

### Sensitivity Analysis

In the sensitivity analysis, recalculated ORs after removing any single eligible study showed that no material alterations were detected implying that our results are reliable and robust (**Supplementary Figures 8–10**).

### Publication Bias

**Figure 11:**
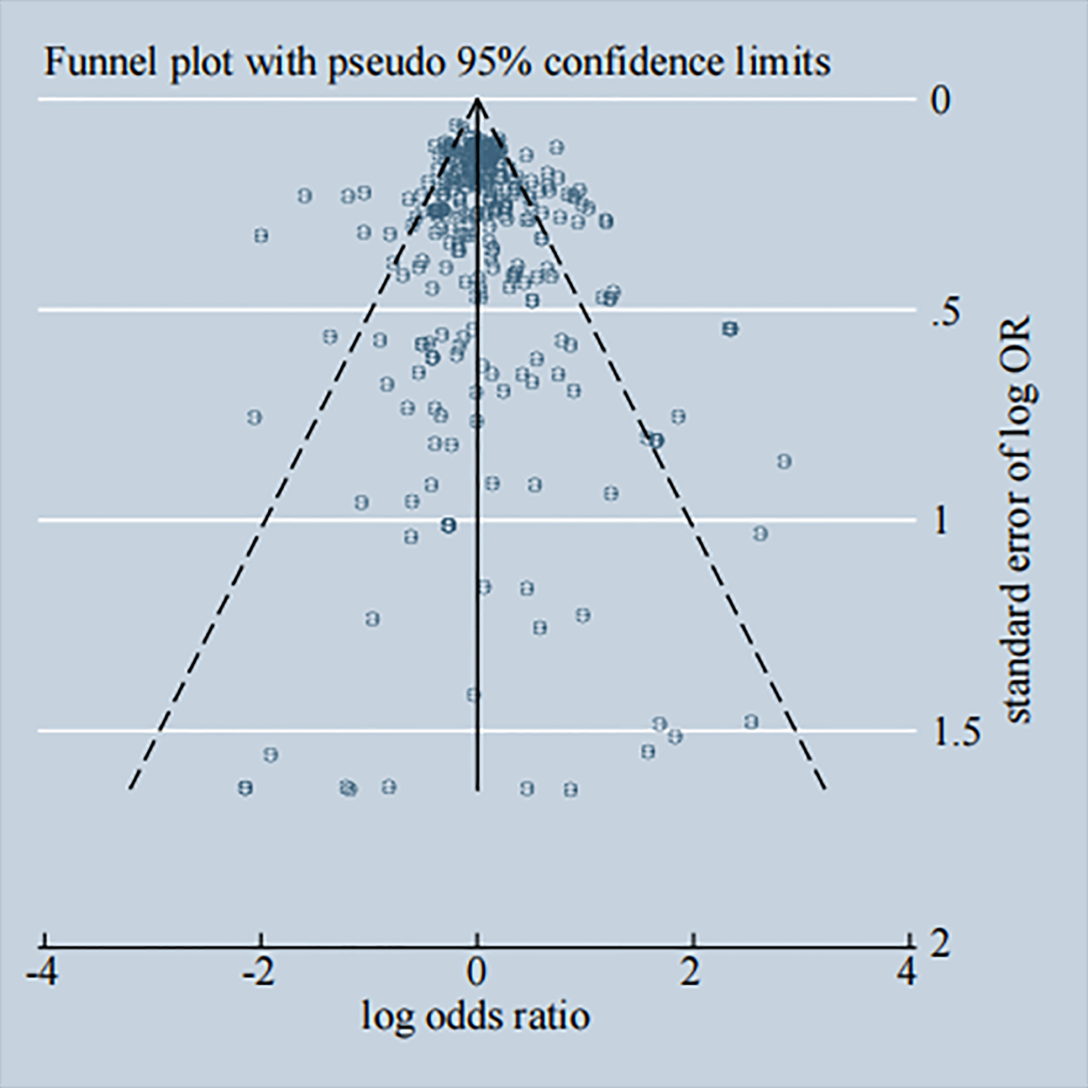
Begg's funnel plot of publication bias.

Potential publication bias among the included studies was assessed through the visual inspection of Begg’s funnel plot, shown in **Figure 11**, that the funnel plot is symmetrical. The P-value of Egger’s test was 0.037, and the regression line crossed the zero line, which indicated the absence of significant publication bias (**Supplementary Figure 11**). Additionally, most of enrolled studies got high scores of NOS quality assessment and details of it are shown in **Supplementary Table 8**.

## Discussion

We analyzed 45 studies, 9 of which involved Caucasians, and 37 studies recruited Asians (25 studies in Chinese, 1 study in Japanese, and 11 studies in Korean). These studies were consistent with the global prevalence of IgAN. Although the prevalence in some Western countries has progressively increased, they still have a lower prevalence of IgAN, and Asian populations such as the Chinese, Japanese, and Korean are more vulnerable to IgAN (44).

The pathogenetic progress of IgAN are mainly characterized by four biochemical hits: (1) aberrant glycosylation of IgA1. (2) anti-glycan/glycopeptides antibodies against unnormal IgA1 synthesized (3) aberrant glycosylation of IgA1 combined with anti- glycan/glycopeptides antibodies so that immune complexes (IC_S_) are formed, (4) of which these IC_S_ accumulated in the glomerular mesangium, induce cell proliferation, and secrete immune molecular to aggravate renal injuries (9, 45).

To avoid generating aberrant IgA1 with galactose-deficient O-glycans, it is necessary to maintain the normal activity and expression of core 1 β1,3­galactosyltransferase 1 (encoded by *C1GALT1*), α­N­acetylgalactosaminide α2,6­sialyltransferase 2 (encoded by *ST6GALNAC2*), and core 1 β1,3­galactosyltransferase-specific chaperone 1 (encoded by *C1GALT1C1*) (46). Two studies enrolled Chinese Han and Chinese Uyghur populations both of which revealed that rs3840858 in *ST6GALNAC2* is associated with susceptibility to IgAN; this was supported by our results. However, a larger patient size and different ethnic populations are required to further bolster the results. Moreover, the mutations of these three molecules are not only associated with the risk of developing IgAN but interactively contribute to the predisposition and severity of it (14, 25). According to a recent Chinese genetic association study and interactive protein network analysis, a possible interaction between *C1GALT1* and *GANT12* through O-glycan processing or the mucin-type O-glycan biosynthesis pathway exists, which is associated with susceptibility to IgAN (33).

Upon investigation of 20 immune pathways, we obtained results consistent with previous research that several significant SNPs were involved in the complement pathway especially the alternative pathway. CFB plays a pivotal role in the production of C3; it combines with C3b to form C3 convertase in the complement response (47). Products of CFH and CFH-related genes co-modulate the activation of alternative complement pathway (48). The SNP rs4151657 in CFB showed stable association with the risk of IgAN in Chinese Han population (under allele dominant overdominant and heterozygote models). It was also associated with higher serum CFB and decreased serum C3 level that indicated poor renal outcomes (49). A single deletion in both CFHR1 and CFHR3 confers a 30% reduction in the risk of IgA nephropathy, and a meta-analysis with risk-score model including Asian, European, and African population evaluated five susceptibility loci of three MHC, one CHF and one HORMAD2. These five loci moderately increased the risk of disease but together explained 4–5% of the predisposition after being examined (30). The region of IgG (FcγRs) and the complement system are engaged in renal injury caused by autoantibody/immune complex and IC clearance (50). C3 is the most common co-deposited molecule with IgA in up to 90% of patients, followed by properdin and complement factor H. Genes encoding low-affinity FcγRs FCGR2A, FCGR3A, FCGR2C, FCGR3B, and FCGR2B (rs12118043, FCRLA rs1954174, FCRLB rs4657093) showed a significant association with the predisposition of IgAN, but the linkage disequilibrium and conditional analysis suggest that these SNPs have a weak association with each other (22).

*TGF-β1* encoded by TGFB1 is a multifunctional cytokine that contributes to cell proliferation, differentiation, expansion of mesangial matrix, the development of fibrosis, and various immunologic processes (51). We conducted a meta-analysis of rs1800469, rs1982073, and rs1800471 in TGFB1 among Korean, Japanese, Italian, and Swedish populations with OR_G_. The result showed that genotype shows a different effect regarding the susceptibility to IgAN according to ethnicity and gender. It is also noteworthy to mention that rs1800469, rs1982073, and rs1800471 shared high LD in the recombination block. Following haplotype analysis, we found that having TAC haplotype significantly increased the risk of IgAn by more than two times (39). Considering SNP effect on prognosis, no significant difference as observed in different genotype as contributing to either progressive or stable IgAN, but patients with homozygote mutation producing higher *TGF-β1* levels show poorer survival due to renal failures (36).

Toll-like receptors (TLRs) are a family of innate immune receptors expressed in the membrane of leukocytes, renal tubular epithelial cells, and various cells which induce inflammatory cytokine expression through intracellular signaling pathway. The agonized TLRs can directly injure kidney and also overproduce antibodies *via* B lymphocytes (52). TLR1 is expressed in tubular epithelial cells and enhances the activation level of NFκB. Our meta-analysis found that rs4833095 and rs5743557 in TLR1 increases the risk of IgAN both in Chinese and Korean pediatric patients group; upon haplotype analysis, we found that T_rs4833095_ and T_rs5743557_ haplotype were associated with IgAN. In European population, the major allele of rs4833095 is T but in African American, sub-Saharan African, and Asian group, the major allele is C allele which corresponded with the prevalence of IgAN. The SNP rs4833095 was associated with the severity of pathology according to Lee’s grades (42).

Interleukin-1 receptors shared the same pathway with TLRs to produce cytokines. IL-1Ra is an anti-inflammatory contributor, while IL-1β as pro-inflammatory, which balances the immune response. In patients with IgAN with a lower IL-1Ra/IL-1βr ratio, more severe pathological changes were observed (53). IL-1B (rs1143627) in Korean pediatric patients show moderate protective effect but the Chinese Han group show a risk of IgAN. The reason for the difference may be that the pediatric patients were prone to have milder lesions in early stage of the disease. Haplotype analysis found CAT showed significant association with the disease (41).

IL-6, a common cytokine, elevated the activity of the GalNAc-specific sialyltransferase encoded by the *ST6GALNAC2 *gene and decreased the activity of core 1 β1,3­galactosyltransferase 1 encoded by *C1GALT1*,* *leading to the accentuation of aberrant glycan-deficient IgA (54).

IL-18 is a cytokine that stimulates the production of several immune molecules such as tumor necrosis factor-α, IFN-γ, and IL-2 to regulate autoimmune conditions. In our meta-analysis which showed that rs1946518 in IL-18 has a robust effect in both Chinese Han and Korean populations. Although the IL-18 level hinted at development of glomerulonephritis and genotype of IL-18 rs1946518CC was related with a higher level of IL-18 mRNA, the current study revealed that rs1946518 may not be associated with clinical symptoms and pathology grade in IgAN (26, 55).

In the results of meta-analysis, in the T-cell co-stimulatory pathway, CTLA4 and ICOS have variant mutations and most of them presented different degree statistical meaning under different genetic models. CTLA4 is a negative regulator in CD28 signaling and plays a key role in the initiation and termination of immune responses, whereas ICOS regulates the activation and tolerance of T cells. The G allele in CTLA4 rs231779 significantly increased the risk and was related to the prevalence of proteinuria (15, 56). As for the correlation between SNPs and clinical symptoms, HLA-DQB1/DRB1 had a high association with a minor allele in rs2856717 that cannot be covered by adjustment of LD structure, protective haplotype (ACAT) and at-risk haplotypes (ATAT, GCGT). Additionally, rs12537 was related to severe proteinuria and rs2523946 indicated a higher incidence of gross hematuria (30). Besides the co-stimulating pathway of T cells, TNFSF13 has a key role in T cell-dependent antibody responses by encode proliferation-inducing ligand (APRIL) which is a powerful cytokine stimulated by Toll-like receptor 9 and other immune-associated factors (57). The minor allele A of TNFSF13(rs3803800) was observed to have a significant association with IgAN under additive and recessive model and was also associated with several clinical phenotypes such as severe proteinuria, low eGFR, and higher occurrence of mesangial hypercellularity (58).

There are several advantages of this meta-analysis. Since genetic comparisons are not independent, the true underlying genetic model is unknown, and the genetic contrasts are defined by merging information of the genotype distribution, we tested six kinds of genetic models to provide as much information as possible to reveal the SNPs in immunologic proteins with susceptibility to IgAN, which decreased the loss of information resolution. Furthermore, the subgroup analysis based on different ethnicities were performed with OR_G_. Genetic variants with a stable and robust risk of susceptibility to IgAN were found through cluster analysis. In addition, we provide a new perspective that combines genetic variability with the inflammatory effects of IgAN and attempts to further explore disease susceptibility and pathological mechanisms.

Our meta-analysis also has some limitations. IgAN is a complex disease, and SNPs without consideration of epistatic genes or gene interactions have reliable and conclusive inferences. We enrolled studies that only included healthy individuals as controls and excluded cohorts of secondary IgAN and comorbidity with other nephropathy diseases. We did not analyze the genetic risk of the subtypes of IgAN, and so we may conduct systematic review and meta-analysis in the future. Considering the large disparities of prevalence in ethnicity, most studies of the same SNP in different ethnicities did not provide sufficient data which limited the subgroup analysis performed. Several of our conclusions are based on individual studies and need to be interpreted with caution.

## Conclusion

The overall meta-analysis results revealed most supportive SNPs in immunology or related pathways significantly associated with susceptibility to IgAN, and six of them were proved by genetic association studies. Furthermore, the subgroup analysis based on ethnicities revealed detailed information. In a polygenic complex disorder such as IgAN, the association of individual polymorphisms in genes may be limited compared with combinations of specific genotypes. However, multiple genetic contrasts may produce diverse results and positive associations resulted from pooling a small number of studies; therefore, these results must be interpreted with caution.

## Data Availability Statement

The original contributions presented in the study are included in the article/**Supplementary Material**. Further inquiries can be directed to the corresponding authors.

## Author Contributions

Conceptualization: XD, YM, ZM, and HZ. Methodology: XD. Validation: XD, YM, and ZM. Formal analysis: XD. Investigation: XD and YM. Resources: XD and HZ. Data curation: XD, YM, and HZ. Writing—original draft preparation: XD. Writing—review and editing: XD, YM, and HZ. Supervision: YY and HZ. Project administration: YY and HZ. Funding acquisition: HZ. All authors contributed to the article and approved the submitted version.

## Funding

This research was funded by The National Natural Science Foundation of China (Nos. 61971441) and the National Key R&D Program of China (Nos. 2016YFC1305500).

## Conflict of Interest

The authors declare that the research was conducted in the absence of any commercial or financial relationships that could be construed as a potential conflict of interest.
