# Supplementary figures and images for: Association of Immune and Inflammatory Gene Polymorphism With the Risk of IgA Nephropathy: A Systematic Review and Meta-Analysis of 45 Studies
Source: Front Immunol. 2021 Jun 30;12:683913. doi: 10.3389/fimmu.2021.683913 (PMC8329849; doi:10.3389/fimmu.2021.683913)

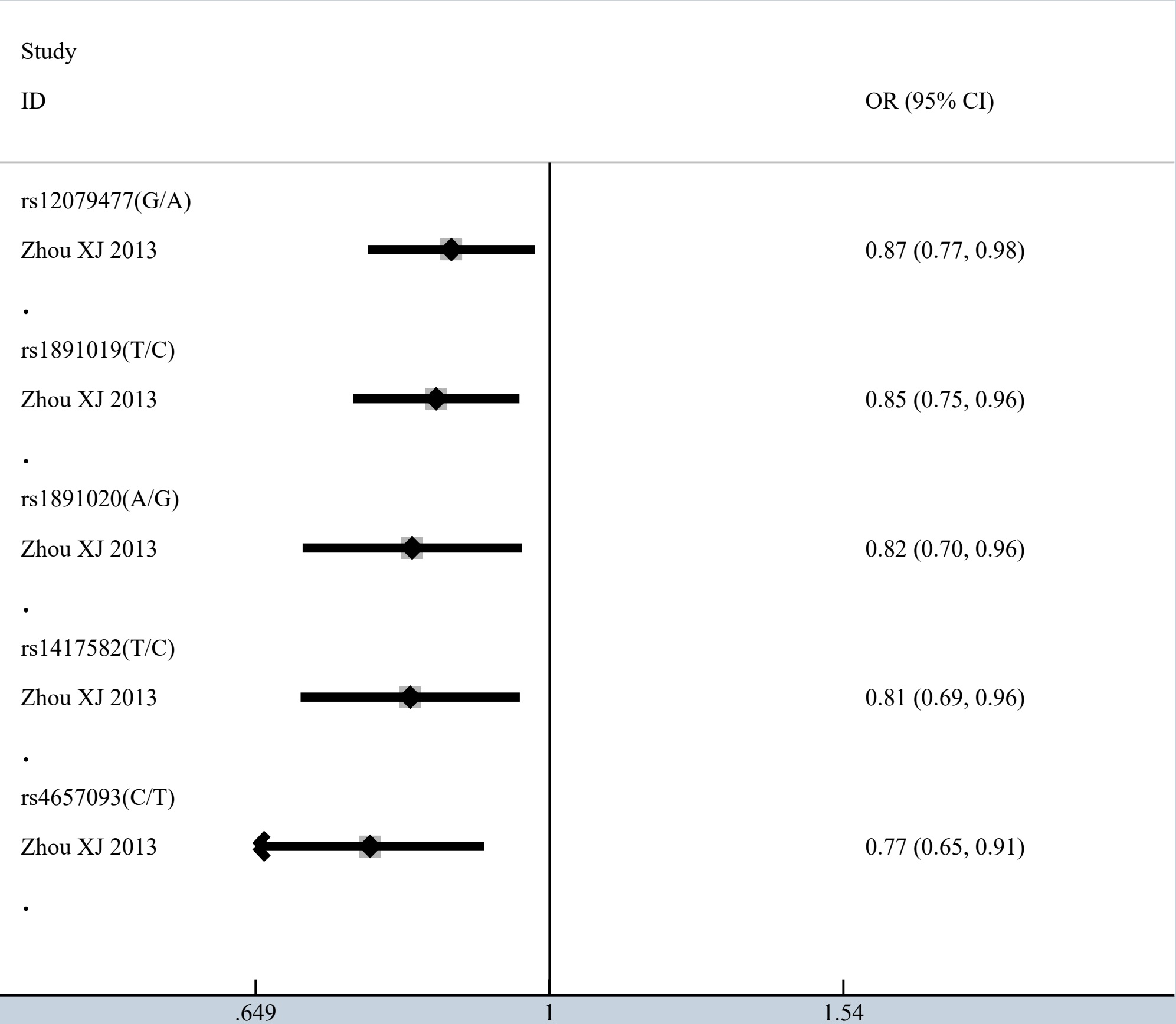

Supplement: Supplementary file 1 [file DataSheet_1.pdf]

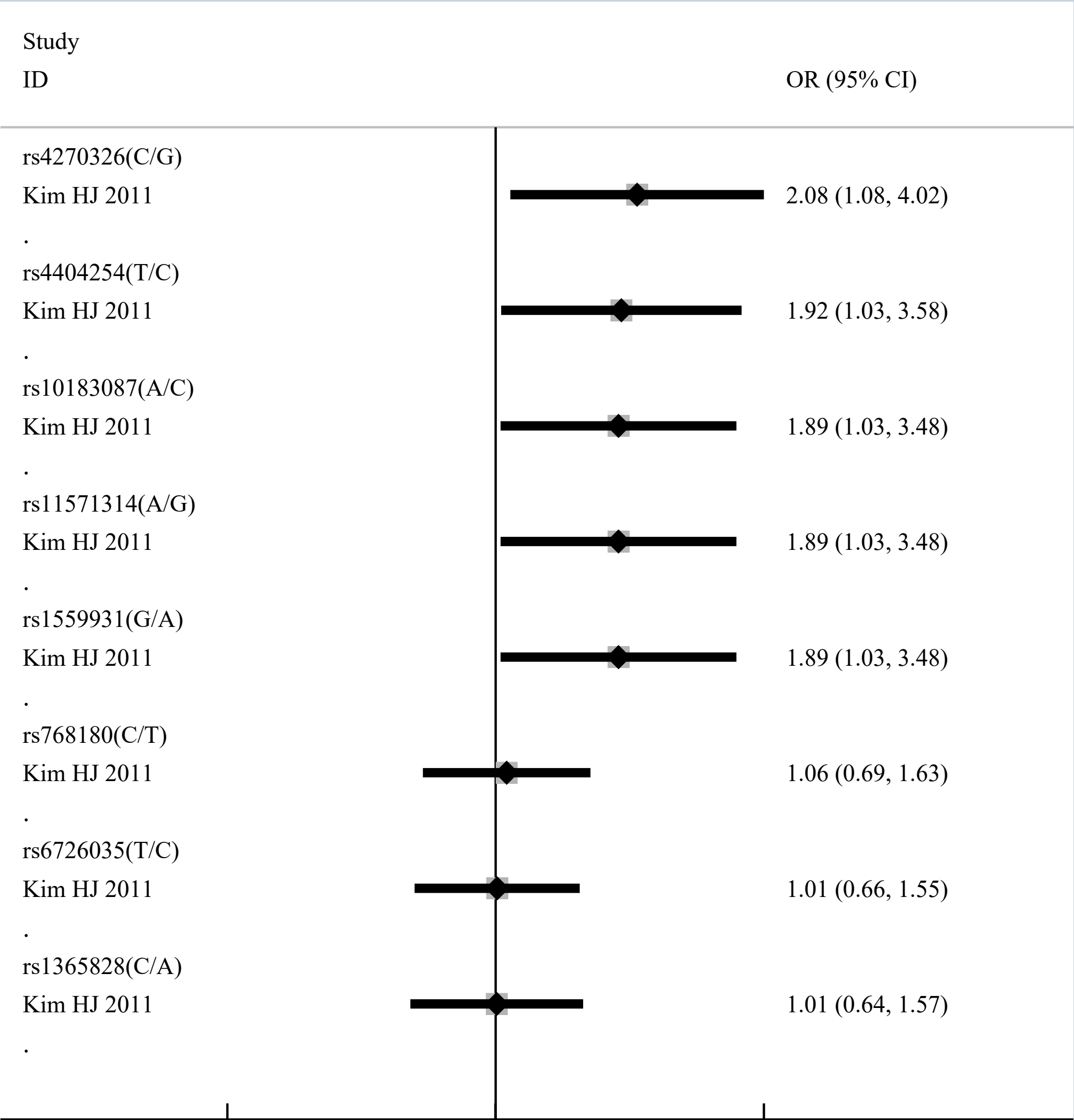

Supplement: Supplementary file 2 [file DataSheet_2.pdf]

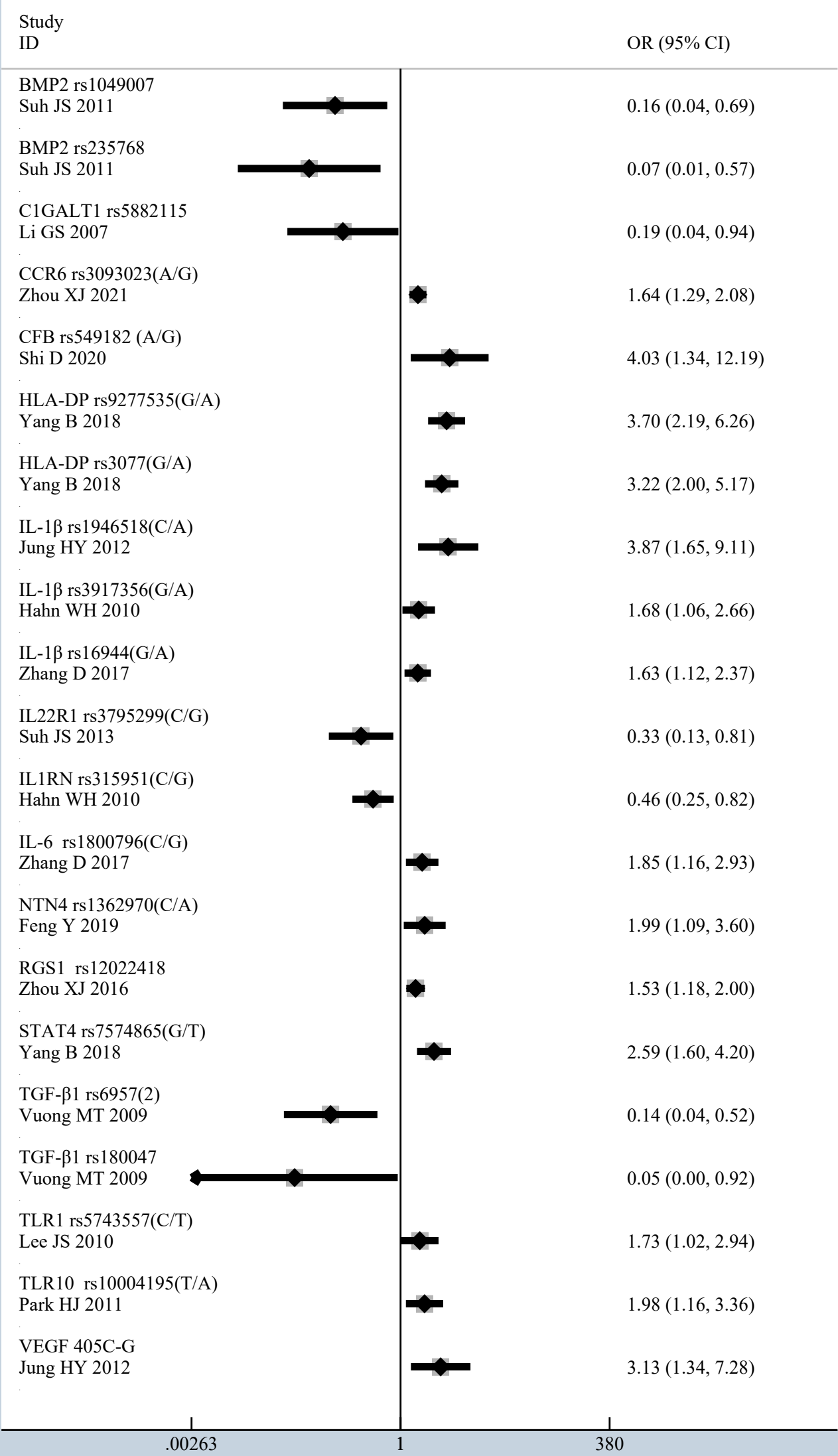

Supplement: Supplementary file 3 [file DataSheet_3.pdf]

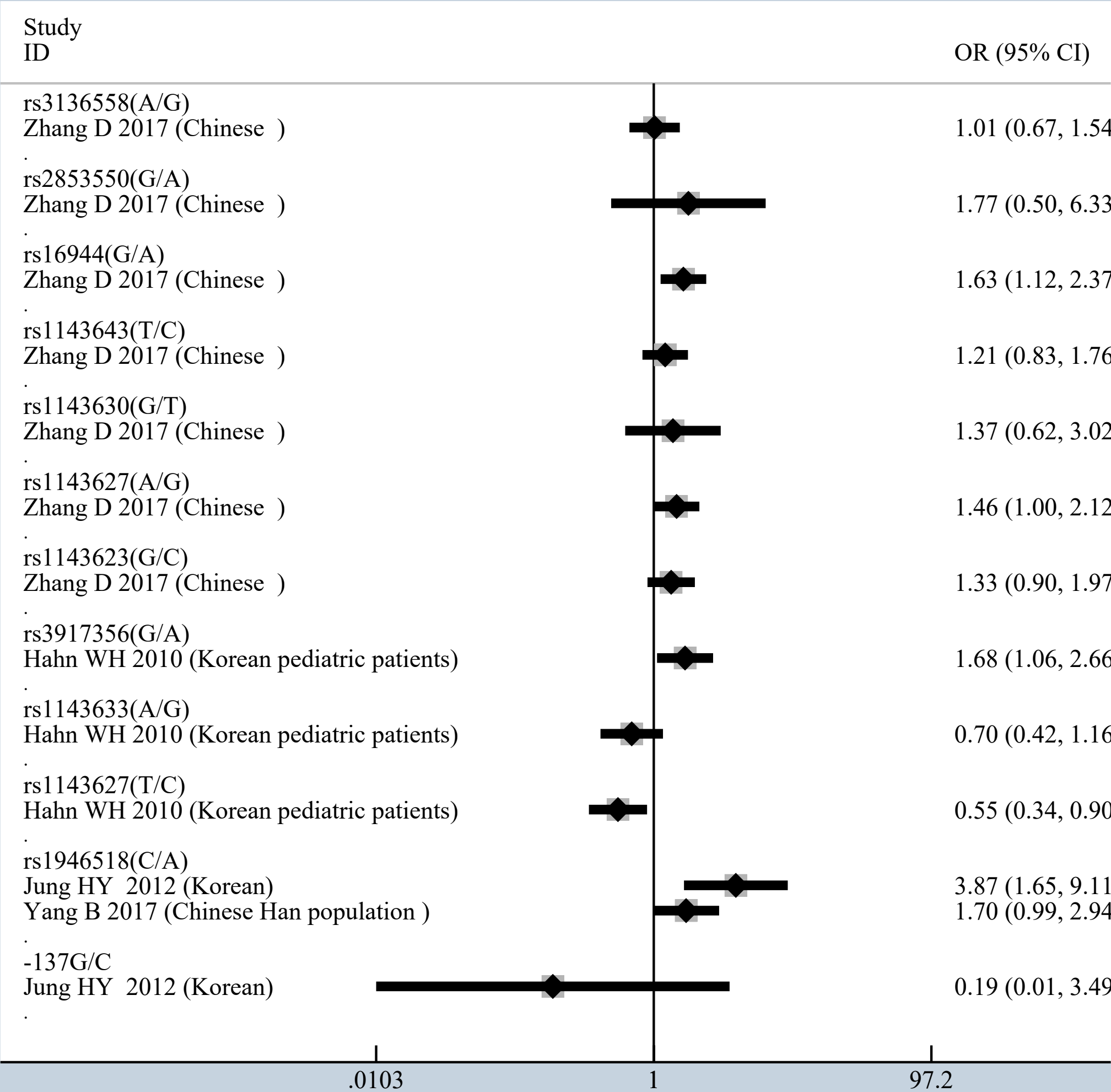

Supplement: Supplementary file 4 [file DataSheet_4.pdf]

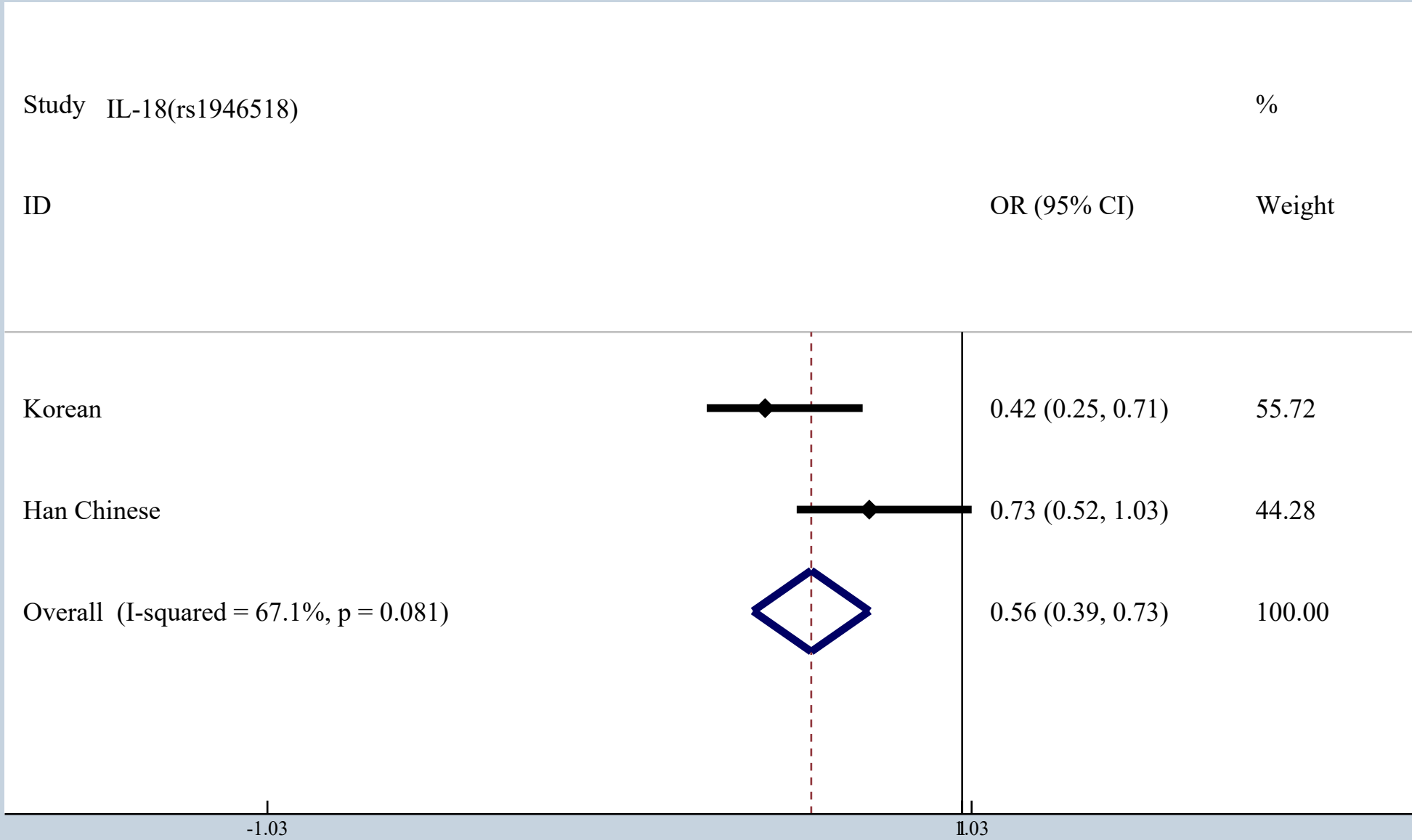

Supplement: Supplementary file 5 [file DataSheet_5.pdf]

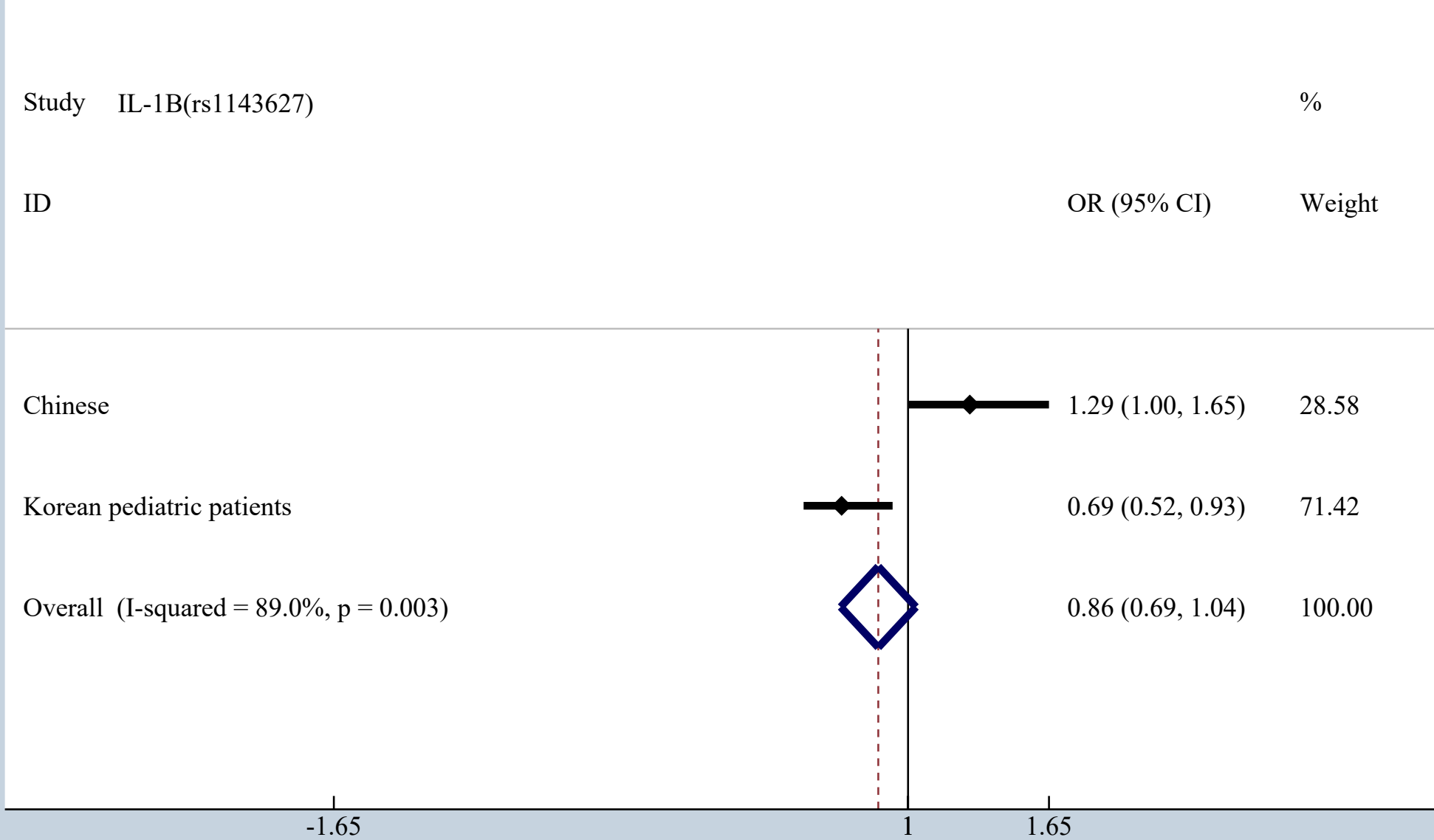

Supplement: Supplementary file 6 [file DataSheet_6.pdf]

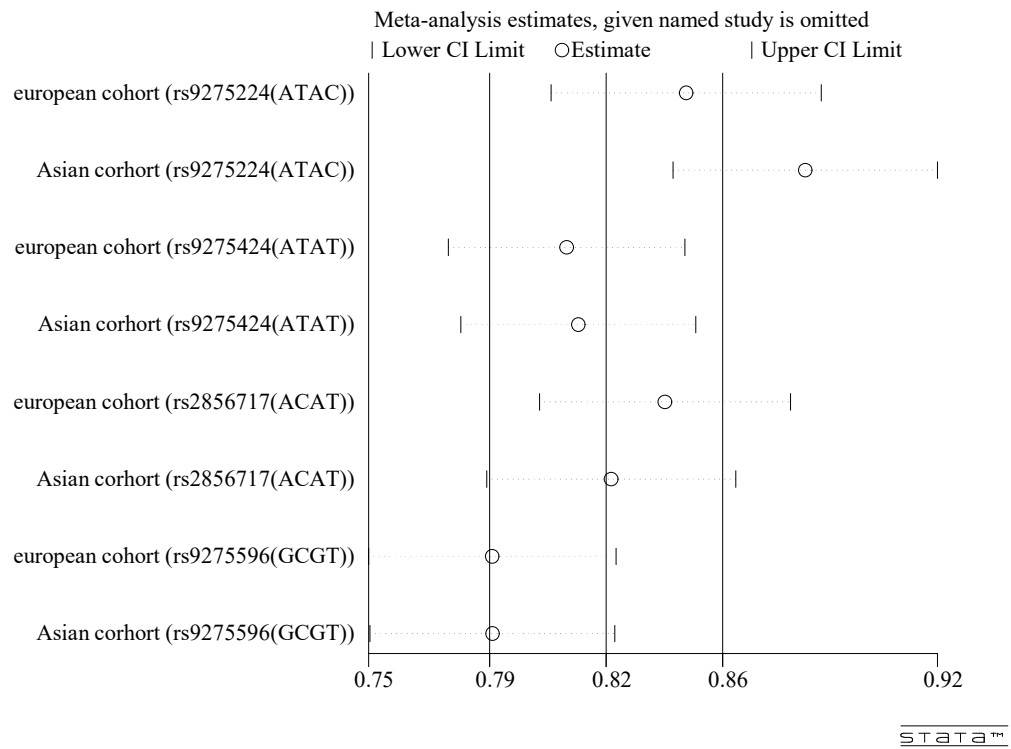

Supplement: Supplementary file 8 [file DataSheet_8.pdf]

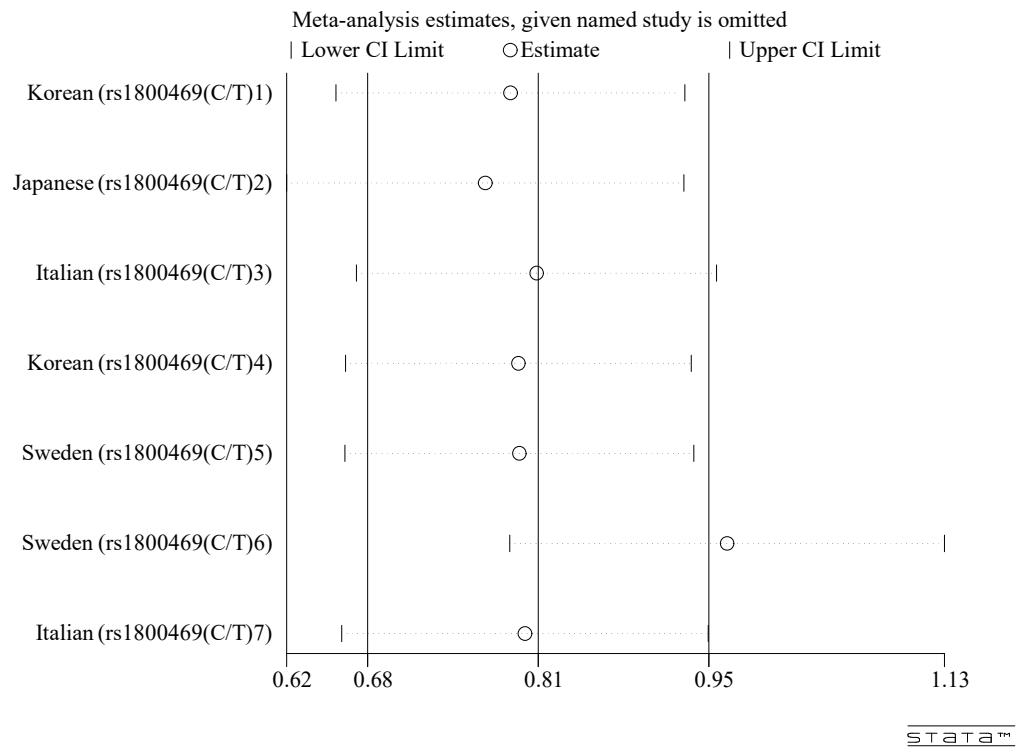

Supplement: Supplementary file 9 [file DataSheet_9.pdf]

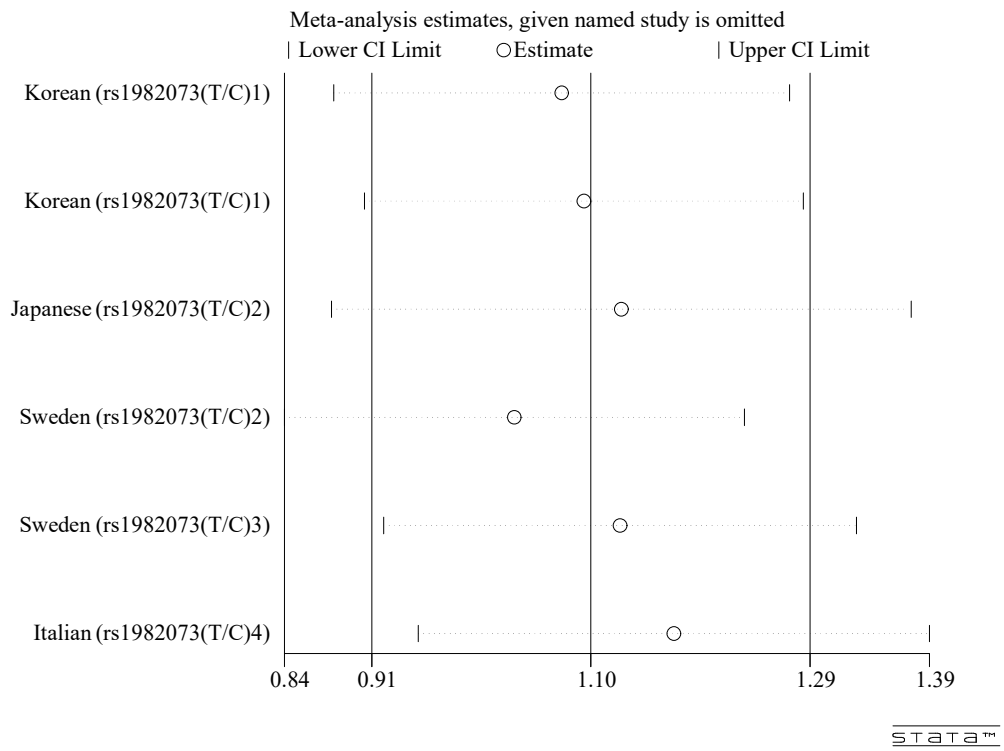

Supplement: Supplementary file 10 [file DataSheet_10.pdf]

● Study

— regression line

|—| 95% CI for intercept

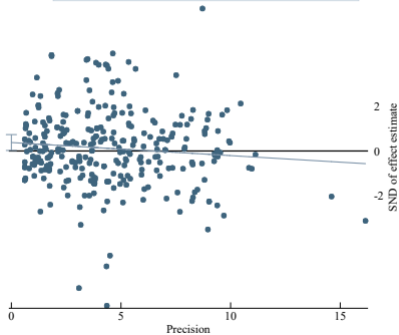

Supplement: Supplementary file 11 [file DataSheet_11.pdf]

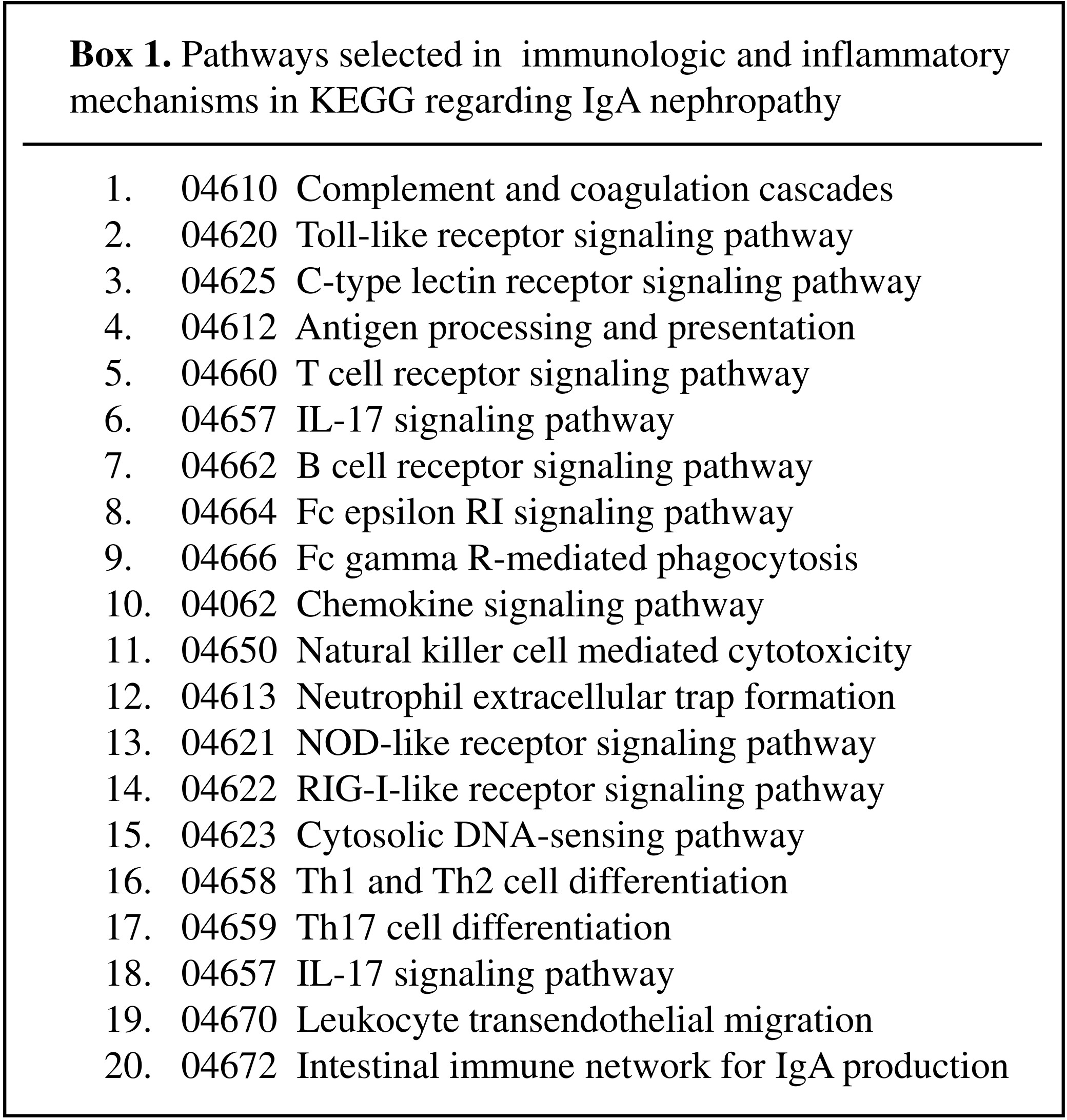

Supplement: Supplementary file 12 [file Image_1.tif]
