# Supplementary material for: Association of Immune and Inflammatory Gene Polymorphism With the Risk of IgA Nephropathy: A Systematic Review and Meta-Analysis of 45 Studies
Source: Front Immunol. 2021 Jun 30;12:683913. doi: 10.3389/fimmu.2021.683913 (PMC8329849; doi:10.3389/fimmu.2021.683913)
Supplement: Supplementary file 7 [file DataSheet_7.pdf]

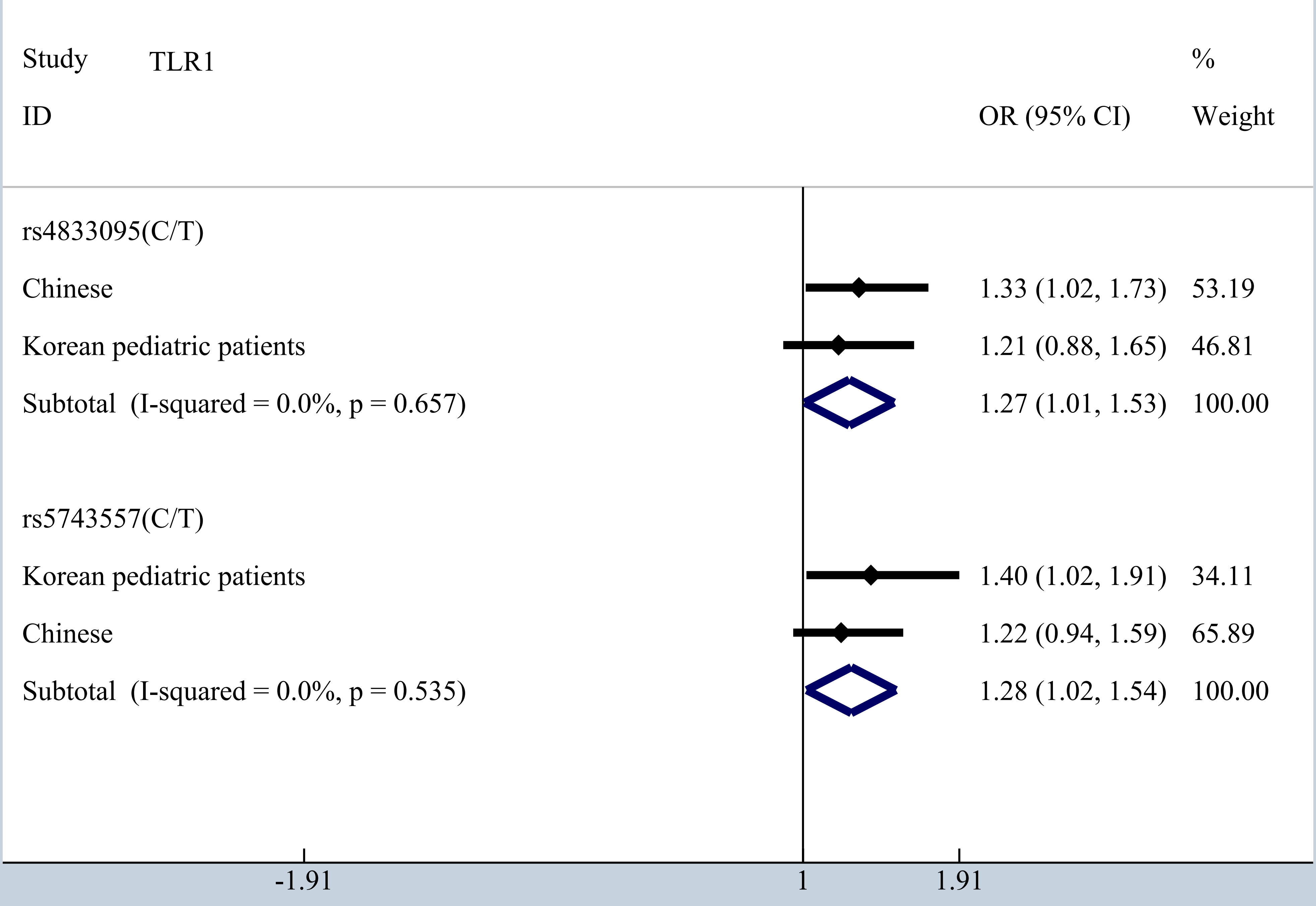

Study TLR1
ID
OR (95% CI)
% Weight
rs4833095(C/T)
Chinese 1.33 (1.02, 1.73) 53.19
Korean pediatric patients 1.21 (0.88, 1.65) 46.81
Subtotal (I-squared = 0.0%, p = 0.657) 1.27 (1.01, 1.53) 100.00
rs5743557(C/T)
Korean pediatric patients 1.40 (1.02, 1.91) 34.11
Chinese 1.22 (0.94, 1.59) 65.89
Subtotal (I-squared = 0.0%, p = 0.535) 1.28 (1.02, 1.54) 100.00
-1.91
1
1.91
