# Supplementary material for: Association of Immune and Inflammatory Gene Polymorphism With the Risk of IgA Nephropathy: A Systematic Review and Meta-Analysis of 45 Studies
Source: Front Immunol. 2021 Jun 30;12:683913. doi: 10.3389/fimmu.2021.683913 (PMC8329849; doi:10.3389/fimmu.2021.683913)
Supplement: Supplementary file 13 [file Table_1.docx]

**Supplementary table1：**

|  | **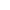**   \| **author** \| \| --- \| | **year** | **ethnicity** | **country** | **PMID** | **case age(years)** | **case**  **male/female (%)** | **control age(years)** | **control male/female (%)** | **case/control** |
| --- | --- | --- | --- | --- | --- | --- | --- | --- | --- | --- | --- |
| 1 | Shi D (1) | 2020 | Chinese | China | [31857673](https://www.ncbi.nlm.nih.gov/pubmed/31857673) | 34.97 ± 11.29 | 49.6/50.4 | 35.16 ± 11.00 | 49.5/50.5 | 1399/1442 |
| 2 | Zhou XJ (2) | 2013 | Chinese | China | 23593433 | NA | NA | NA | NA | 1194/900 |
| 3 | Wang H (3) | 2015 | Chinese | China | 25193896 | Mean=41.6  (15-55) | 52.4/47.6 | Mean=44.32  (34-54) | NA | 21/37 |
| 4 | Szelestei T (4) | 2000 | Caucasians | Hungary | 10977777 | NA | 66.4/33.6 | NA | NA | 110/104 |
| 5 | Steinmetz OM (5) | 2004 | Caucasians | German | 14767014 | 38.59±14.5 | 96.6/3.4 | 38 ±12.3 | 1/0 | 207/140 |
| 6 | Lee JS (6) | 2010 | Korean pediatric patients | Korea | 21108742 | 12.77 ± 5.15 | 60/40 | 37.14 ± 13.14 | 55.8/44.2 | 190/283 |
| 7 | Park HJ (7) | 2011 | Korean pediatric patients | Korea | 20953797 | Mean=12.0  (9.5–14.3) | 59.3/40.7 | Mean=30.0  (29.0–52.0) | 54.7/45.3 | 199/289 |
| 8 | Gao J(8) | 2018 | Chinese | China | [28391282](https://www.ncbi.nlm.nih.gov/pubmed/28391282) | 32±11.9 | 65.2/34.8 | 35±12.6 | 60/40 | 351/310 |
| 9 | Zhang D (9) | 2017 | Chinese | China | 29069743 | 33.22±12.15 | 65.5/34.5 | 50.65 ±11.79 | 57.2/42.8 | 417/463 |
| 10 | Gao J (10) | 2017 | Chinese | China | 28359052 | 32±11.9 | 65.2/34.8 | 35±12.6 | 60/40 | 351/310 |
| 11 | Wu C (11) | 2017 | Chinese | China | 29100328 | NA | 37.4/62.6 | NA | NA | 586/606 |
| 12 | Kim HJ (12) | 2011 | Korean pediatric patients | Korea | 21677403 | Boys:12.55 ± 5.65  Girls: 11.04 ±5.16 | 56.4/43.6 | Men: 38.57±10.03  Women: 39.13±9.20 | 44.1/55.9 | 172/399 |
| 13 | Zhou XJ (13) | 2016 | Chinese | China | 27804980 | NA | NA | NA | NA | 1248/1187 |
| 14 | Suh JS (14) | 2011 | Korean pediatric patients | Korea | 21214373 | Boys:12.58±4.66  Girls:11.57±5.31 | 58.3/41.7 | 38.8±9.52/ 40.39±9.21 | 44/56 | 192/397 |
| 15 | Cheng W (15) | 2011 | Chinese | China | 21245129 | 34.4±11.7 | 51/49 | 31.9±8.7 | 65.4/34.6 | 527/522 |
| 16 | Zhou XJ (16) | 2021 | Chinese | China | 33462083 | 33±9 | 55/45 | 32±7 | 68/32 | 1194/902 |
|  |  |  |  |  |  | 36±10 | 51/49 | 36±8 | 43/57 | 640/4295 |
|  |  |  |  |  |  | 37±10 | 55/45 | 35±11 | 49/51 | 500/4123 |
|  |  |  |  |  |  | 36±9 | 50/50 | 36±8 | 51/49 | 1230/1922 |
| 17 | Yang B (17) | 2018 | Chinese | China | 29467950 | 33.79 ± 8.96 | 50/50 | 37.82 ± 9.89 | 62.7/37.3 | 140/490 |
| 18 | Jacob M (18) | 2018 | Caucasians | German | 29539619 | NA | NA | NA | NA | 455/252 |
| 19 | Wolf G (19) | 2002 | Caucasians | German | 12147803 | NA | 71.7/28.3 | NA | (20-55) | 127/152 |
| 20 | Wei L (20) | 2018 | Chinese | China | 29402846 | NA | NA | NA | NA | 351/310 |
| 21 | Li GS (21) | 2007 | Chinese | China | 17228361 | NA | NA | NA | NA | 670/494 |
| 22 | Hahn WH (22) | 2010 | Korean pediatric patients | Korea | 19280228 | Boys:12.54±5.51  Gilrs:11.15±5.11 | 57.1/42.9 | Men:42.52±12.41  Women:44.49±13.33 | 43.2/56.8 | 182/500 |
| 23 | Jung HY (23) | 2012 | Korean | Korea | 26889427 | 34.22±13.50 | 60.9/39.1 | 42.40±11.73 | 19.9/80.1 | 69/146 |
| 24 | Yang B (24) | 2017 | Chinese Han population | China | 27028244 | 34.4± 9.5 | 52/48 | 38.3± 10.2 | 51/49 | 166/198 |
| 25 | Mao J (25) | 2007 | Chinese children | China | 17635752 | (2.8-16.5) | 61.5/38.5 | NA | NA | 26/30 |
| 26 | Gao J (26) | 2015 | Chinese | China | 26588355 | 32 ±11.9 | 65.2/34.8 | 35±12.6 | 60/40 | 351/310 |
| 27 | Wei LT (27) | 2016 | Chinese | China | 26871801 | 32± 11.9 | 65.2/34.8 | 35±12.6 | 60/40 | 351/310 |
| 28 | Gao J (28) | 2016 | Chinese | China | 27806314 | 32± 11.9 | 65.2/34.8 | 35±12.6 | 60/40 | 351/311 |
| 29 | Gao J (29) | 2017 | Chinese | China | 28946141 | 32± 11.9 | 65.2/34.8 | 35±12.6 | 60/40 | 351/310 |
| 30 | Xia YF (30) | 2006 | Chinese | China | 16550745 | 30.1 ± 8.5 | 41/59 | NA | NA | 435/100 |
| 31 | Lim CS (31) | 2008 | Korean | Korea | 18793525 | 34.2±14.2 | 57.7/42.3 | 47.5±9.9 | 49.5/50.5 | 260/315 |
| 32 | Hahn WH (32) | 2010 | Korean pediatric patients | Korea | 20563733 | 11.75±3.90 | 55/45 | 42.68±12.61 | 44/56 | 160/454 |
| 33 | Zhong Z (33) | 2017 | Chinese | China | 28636766 | 34.70 ± 11.19 | 48.3/51.7 | 34.96 ± 10.91 | 48.7/51.3 | 962/963 |
| 34 | Feng Y (34) | 2019 | Chinese | China | 30928649 | 32.44 ± 11.80 | 65.3/34.7 | 51.16 ± 11.49 | 72.4/27.6 | 357/384 |
| 35 | Shi D (35) | 2020 | Chinese | China | 31227791 | 34.63 ± 11.24 | 48.7/51.3 | 34.84 ± 10.94 | 48.8/51.2 | 1000/1000 |
| 36 | Lu C (36) | 2015 | Chinses Uyghur | China | 26136946 | 38.81±11.06 | 47.8/52.2 | 37.53±11.68 | 46.7/53.3 | 180/180 |
| 37 | Fu D (37) | 2020 | Chinese | China | 32747022 | 34.64 ± 11.19 | 48.43/51.57 | 34.94 ± 10.90 | 48.43/51.57 | 960/956 |
| 38 | Liu XQ (38) | 2008 | Caucasians | Canada | 18256354 | 45 (43.2–46.8) | 65/35 | 45 (42.4–47.6) | 66.7/33.3 | 206/111 |
|  |  |  |  |  |  | 48 (46.3–49.7) | 66.7/33.3 | 47 (44.9–49.2) | 69.5/30.5 | 255/187 |
|  |  |  |  |  |  | 41 (39.3–42.7) | 73.1/26.9 | 43 (41.7–44.3) | 69.8/30.2 | 271/205 |
| 39 | Sato F (39) | 2004 | Japanese | Japan | 15191521 | NA | NA | NA | 49.2/50.8 | 329/297 |
| 40 | Carturan S (40) | 2004 | Caucasians | Italy | 15593052 | 48.1 ± 13.79 | 72.3/27.3 | NA | 69.5/30.5 | 101/118 |
| 41 | Lim CS (41) | 2005 | Korean | Korea | 15730046 | 34.6 ± 1.5 | 64.8/35.2 | 53.7 ± 1.2 | 65.5/34.5 | 108/54 |
| 42 | Vuong MT (42) | 2009 | Sweden | Sweden | 19258388 | 38.5 ± 14.4 | 68.9/31.1 | 44.8 ± 13.0 | 67.3/32.7 | 212/477 |
| 43 | Brezzi B (43) | 2009 | Italian | Italy | 19967654 | 33.4 ± 11.2 | 71.4/28.6 | NA | NA | 105/200 |
| 44 | Suh JS (44) | 2011 | Korean pediatric patients | Korea | 22977507 | 38.2±13.8 | NA | 12.4±4.5 | NA | 187/262 |
| 45 | Suh JS (45) | 2013 | Korean pediatric patients | Korea | 23659670 | 12.55±5.14 | 58.8/41.2 | 37.51±13.55 | 54.4/45.6 | 194/287 |

1. Shi D, Zhong Z, Wang M, Cai L, Fu D, Peng Y, et al. Identification of susceptibility locus shared by IgA nephropathy and inflammatory bowel disease in a Chinese Han population. *J Hum Genet* (2020) 65(3):241-9. Epub 2019/12/21. doi: 10.1038/s10038-019-0699-9. PubMed PMID: 31857673.

2. Zhou XJ, Cheng FJ, Qi YY, Zhao YF, Hou P, Zhu L, et al. FCGR2B and FCRLB gene polymorphisms associated with IgA nephropathy. *PLoS One* (2013) 8(4):e61208. Epub 2013/04/18. doi: 10.1371/journal.pone.0061208. PubMed PMID: 23593433; PubMed Central PMCID: PMCPMC3625155.

3. Wang H, Sui W, Xue W, Wu J, Chen J, Dai Y. Univariate and multiple linear regression analyses for 23 single nucleotide polymorphisms in 14 genes predisposing to chronic glomerular diseases and IgA nephropathy in Han Chinese. *Saudi J Kidney Dis Transpl* (2014) 25(5):992-7. Epub 2014/09/07. doi: 10.4103/1319-2442.139882. PubMed PMID: 25193896.

4. Szelestei T, Bähring S, Kovács T, Vas T, Salamon C, Busjahn A, et al. Association of a uteroglobin polymorphism with rate of progression in patients with IgA nephropathy. *Am J Kidney Dis* (2000) 36(3):468-73. Epub 2000/09/08. doi: 10.1053/ajkd.2000.9786. PubMed PMID: 10977777.

5. Steinmetz OM, Panzer U, Harendza S, Mertens PR, Ostendorf T, Floege J, et al. No association of the -2518 MCP-1 A/G promoter polymorphism with incidence and clinical course of IgA nephropathy. *Nephrol Dial Transplant* (2004) 19(3):596-601. Epub 2004/02/10. doi: 10.1093/ndt/gfg577. PubMed PMID: 14767014.

6. Lee JS, Park HK, Suh JS, Hahn WH, Kang SW, Park HJ, et al. Toll-like receptor 1 gene polymorphisms in childhood IgA nephropathy: a case-control study in the Korean population. *Int J Immunogenet* (2011) 38(2):133-8. Epub 2010/11/27. doi: 10.1111/j.1744-313X.2010.00978.x. PubMed PMID: 21108742.

7. Park HJ, Hahn WH, Suh JS, Kim MJ, Kang SW, Lee JS, et al. Association between toll-like receptor 10 (TLR10) gene polymorphisms and childhood IgA nephropathy. *Eur J Pediatr* (2011) 170(4):503-9. Epub 2010/10/19. doi: 10.1007/s00431-010-1325-1. PubMed PMID: 20953797.

8. Gao J, Wei L, Liu X, Wang L, Niu D, Jin T, et al. Association Between IFN-γ Gene Polymorphisms and IgA Nephropathy in a Chinese Han Population. *Kidney Blood Press Res* (2017) 42(1):136-44. Epub 2017/04/10. doi: 10.1159/000473889. PubMed PMID: 28391282.

9. Zhang D, Xie M, Yang X, Zhang Y, Su Y, Wang Y, et al. Determination of IL-1B (rs16944) and IL-6 (rs1800796) genetic polymorphisms in IgA nephropathy in a northwest Chinese Han population. *Oncotarget* (2017) 8(42):71750-8. Epub 2017/10/27. doi: 10.18632/oncotarget.17603. PubMed PMID: 29069743; PubMed Central PMCID: PMCPMC5641086.

10. Gao J, Wei L, Fu R, Wei J, Niu D, Wang L, et al. Association of Interleukin-10 Polymorphisms (rs1800872, rs1800871, and rs1800896) with Predisposition to IgA Nephropathy in a Chinese Han Population: A Case-Control Study. *Kidney Blood Press Res* (2017) 42(1):89-98. Epub 2017/03/31. doi: 10.1159/000471899. PubMed PMID: 28359052.

11. Wu C, Li G, Wang L. The interaction effect of rs4077515 and rs17019602 increases the susceptibility to IgA nephropathy. *Oncotarget* (2017) 8(44):76492-7. Epub 2017/11/05. doi: 10.18632/oncotarget.20401. PubMed PMID: 29100328; PubMed Central PMCID: PMCPMC5652722.

12. Kim HJ, Chung JH, Kang S, Kim SK, Cho BS, Kim SD, et al. Association of CTLA4, CD28 and ICOS gene polymorphisms with clinicopathologic characteristics of childhood IgA nephropathy in Korean population. *J Genet* (2011) 90(1):151-5. Epub 2011/06/17. doi: 10.1007/s12041-011-0042-5. PubMed PMID: 21677403.

13. Zhou XJ, Nath SK, Qi YY, Sun C, Hou P, Zhang YM, et al. Novel identified associations of RGS1 and RASGRP1 variants in IgA Nephropathy. *Sci Rep* (2016) 6:35781. Epub 2016/11/03. doi: 10.1038/srep35781. PubMed PMID: 27804980; PubMed Central PMCID: PMCPMC5090199.

14. Suh JS, Hahn WH, Cho BS. Polymorphisms of CXCL8 and its receptor CXCR2 contribute to the development and progression of childhood IgA nephropathy. *J Interferon Cytokine Res* (2011) 31(3):309-15. Epub 2011/01/11. doi: 10.1089/jir.2010.0031. PubMed PMID: 21214373.

15. Cheng W, Zhou X, Zhu L, Shi S, Lv J, Liu L, et al. Polymorphisms in the nonmuscle myosin heavy chain 9 gene (MYH9) are associated with the progression of IgA nephropathy in Chinese. *Nephrol Dial Transplant* (2011) 26(8):2544-9. Epub 2011/01/20. doi: 10.1093/ndt/gfq768. PubMed PMID: 21245129.

16. Zhou XJ, Tsoi LC, Hu Y, Patrick MT, He K, Berthier CC, et al. Exome Chip Analyses and Genetic Risk for IgA Nephropathy among Han Chinese. *Clin J Am Soc Nephrol* (2021) 16(2):213-24. Epub 2021/01/20. doi: 10.2215/cjn.06910520. PubMed PMID: 33462083; PubMed Central PMCID: PMCPMC7863642.

17. Yang B, Zhang J, Liu X, Huang Z, Su Z, Liao Y, et al. Genetic polymorphisms in HLA-DP and STAT4 are associated with IgA nephropathy in a Southwest Chinese population. *Oncotarget* (2018) 9(6):7066-74. Epub 2018/02/23. doi: 10.18632/oncotarget.23829. PubMed PMID: 29467950; PubMed Central PMCID: PMCPMC5805536.

18. Jacob M, Ohl K, Goodarzi T, Harendza S, Eggermann T, Fitzner C, et al. CTLA-4 Polymorphisms in Patients with IgA Nephropathy Correlate with Proteinuria. *Kidney Blood Press Res* (2018) 43(2):360-6. Epub 2018/03/15. doi: 10.1159/000488069. PubMed PMID: 29539619.

19. Wolf G, Panzer U, Harendza S, Wenzel U, Stahl RA. No association between a genetic variant of the p22(phox) component of NAD(P)H oxidase and the incidence and progression of IgA nephropathy. *Nephrol Dial Transplant* (2002) 17(8):1509-12. Epub 2002/07/31. doi: 10.1093/ndt/17.8.1509. PubMed PMID: 12147803.

20. Wei L, Fu R, Liu X, Wang L, Wang M, Yu Q, et al. Rs1520220 and Rs2195239 Polymorphisms of IGF-1 Gene Associated with Histopathological Grades in IgA Nephropathy in Northwestern Chinese Han Population. *Kidney Blood Press Res* (2018) 43(1):80-7. Epub 2018/02/07. doi: 10.1159/000486914. PubMed PMID: 29402846.

21. Li GS, Zhang H, Lv JC, Shen Y, Wang HY. Variants of C1GALT1 gene are associated with the genetic susceptibility to IgA nephropathy. *Kidney Int* (2007) 71(5):448-53. Epub 2007/01/18. doi: 10.1038/sj.ki.5002088. PubMed PMID: 17228361.

22. Hahn WH, Cho BS, Kim SD, Kim SK, Kang S. Interleukin-1 cluster gene polymorphisms in childhood IgA nephropathy. *Pediatr Nephrol* (2009) 24(7):1329-36. Epub 2009/03/13. doi: 10.1007/s00467-009-1146-5. PubMed PMID: 19280228.

23. Jung HY, Cho JH, Lim JH, Yu CH, Choi JY, Yoon SH, et al. Impact of gene polymorphisms of interleukin-18, transforming growth factor-β, and vascular endothelial growth factor on development of IgA nephropathy and thin glomerular basement membrane disease. *Kidney Res Clin Pract* (2012) 31(4):234-41. Epub 2012/12/01. doi: 10.1016/j.krcp.2012.09.006. PubMed PMID: 26889427; PubMed Central PMCID: PMCPMC4716104.

24. Yang B, Feng W, Li Y, Shi Y, Cai B, Liao Y, et al. Interleukin 18 -607 A/C Gene Polymorphism is Associated With Susceptibility to IgA Nephropathy in a Chinese Han Population. *Appl Immunohistochem Mol Morphol* (2017) 25(10):725-30. Epub 2016/03/31. doi: 10.1097/pai.0000000000000364. PubMed PMID: 27028244.

25. Mao J, Du L, Gu W, Dai Y, Liu A, Xia Y, et al. Lack of association between NPHS2 gene polymorphisms and sporadic IgA nephropathy. *Nephrology (Carlton)* (2007) 12(4):371-5. Epub 2007/07/20. doi: 10.1111/j.1440-1797.2007.00803.x. PubMed PMID: 17635752.

26. Gao J, Yu QL, Fu RG, Wei LT, Wang M, Dong FM, et al. Lack of Association Between Polymorphisms in AGT and ATR1 and IgA Nephropathy in a Chinese Population. *Genet Test Mol Biomarkers* (2015) 19(12):710-3. Epub 2015/11/21. doi: 10.1089/gtmb.2015.0167. PubMed PMID: 26588355.

27. Wei LT, Fu RG, Gao J, Yu QL, Dong FM, Wang Z, et al. Association of Megsin Gene Variants With IgA Nephropathy in Northwest Chinese Population: A STROBE-Compliant Observational Study. *Medicine (Baltimore)* (2016) 95(6):e2694. Epub 2016/02/13. doi: 10.1097/md.0000000000002694. PubMed PMID: 26871801; PubMed Central PMCID: PMCPMC4753896.

28. Gao J, Wei L, Wei J, Yao G, Wang L, Wang M, et al. TLR1 polymorphism rs4833095 as a risk factor for IgA nephropathy in a Chinese Han population: A case-control study. *Oncotarget* (2016) 7(50):83031-9. Epub 2016/11/03. doi: 10.18632/oncotarget.12965. PubMed PMID: 27806314; PubMed Central PMCID: PMCPMC5347750.

29. Gao J, Wang M, Wei L, Niu D, Wei J, Ou Y, et al. The Endothelial Nitric Oxide Synthase Gene Polymorphism is Associated with the Susceptibility to Immunoglobulin a Nephropathy in Chinese Population. *Kidney Blood Press Res* (2017) 42(3):608-16. Epub 2017/09/26. doi: 10.1159/000481421. PubMed PMID: 28946141.

30. Xia YF, Huang S, Li X, Yang N, Huang J, Xue C, et al. A family-based association study of megsin A23167G polymorphism with susceptibility and progression of IgA nephropathy in a Chinese population. *Clin Nephrol* (2006) 65(3):153-9. Epub 2006/03/23. doi: 10.5414/cnp65153. PubMed PMID: 16550745.

31. Lim CS, Kim SM, Oh YK, Joo KW, Kim YS, Han JS, et al. Megsin 2093T-2180C haplotype at the 3' untranslated region is associated with poor renal survival in Korean IgA nephropathy patients. *Clin Nephrol* (2008) 70(2):101-9. Epub 2008/09/17. doi: 10.5414/cnp70101. PubMed PMID: 18793525.

32. Hahn WH, Suh JS, Cho BS. Phosphodiesterase-5 gene (PDE5A) polymorphisms are associated with progression of childhood IgA nephropathy. *Pediatr Nephrol* (2010) 25(9):1663-71. Epub 2010/06/22. doi: 10.1007/s00467-010-1579-x. PubMed PMID: 20563733.

33. Zhong Z, Feng SZ, Xu RC, Li ZJ, Huang FX, Yin PR, et al. Association of TNFSF13 polymorphisms with IgA nephropathy in a Chinese Han population. *J Gene Med* (2017) 19(6-7). Epub 2017/06/22. doi: 10.1002/jgm.2966. PubMed PMID: 28636766.

34. Feng Y, Su Y, Ma C, Jing Z, Yang X, Zhang D, et al. 3'UTR variants of TNS3, PHLDB1, NTN4, and GNG2 genes are associated with IgA nephropathy risk in Chinese Han population. *Int Immunopharmacol* (2019) 71:295-300. Epub 2019/04/01. doi: 10.1016/j.intimp.2019.03.041. PubMed PMID: 30928649.

35. Shi D, Zhong Z, Xu R, Li B, Li J, Habib U, et al. Association of ITGAX and ITGAM gene polymorphisms with susceptibility to IgA nephropathy. *J Hum Genet* (2019) 64(9):927-35. Epub 2019/06/23. doi: 10.1038/s10038-019-0632-2. PubMed PMID: 31227791.

36. Lu C, Li WL, Ma YR. Study of correlation between polymorphism of ST6GALNAC2 and susceptibility to IgA nephropathy. *Exp Ther Med* (2015) 9(6):2127-32. Epub 2015/07/03. doi: 10.3892/etm.2015.2404. PubMed PMID: 26136946; PubMed Central PMCID: PMCPMC4473512.

37. Fu D, Zhong Z, Shi D, Peng Y, Li B, Wang D, et al. ST6GAL1 polymorphisms influence susceptibility and progression of IgA nephropathy in a Chinese Han population. *Immunobiology* (2020) 225(4):151973. Epub 2020/08/05. doi: 10.1016/j.imbio.2020.151973. PubMed PMID: 32747022.

38. Liu XQ, Paterson AD, He N, St George-Hyslop P, Rauta V, Gronhagen-Riska C, et al. IL5RA and TNFRSF6B gene variants are associated with sporadic IgA nephropathy. *J Am Soc Nephrol* (2008) 19(5):1025-33. Epub 2008/02/08. doi: 10.1681/asn.2007091013. PubMed PMID: 18256354; PubMed Central PMCID: PMCPMC2386732.

39. Sato F, Narita I, Goto S, Kondo D, Saito N, Ajiro J, et al. Transforming growth factor-beta1 gene polymorphism modifies the histological and clinical manifestations in Japanese patients with IgA nephropathy. *Tissue Antigens* (2004) 64(1):35-42. Epub 2004/06/12. doi: 10.1111/j.1399-0039.2004.00256.x. PubMed PMID: 15191521.

40. Carturan S, Roccatello D, Menegatti E, Di Simone D, Davit A, Piazza A, et al. Association between transforming growth factor beta1 gene polymorphisms and IgA nephropathy. *J Nephrol* (2004) 17(6):786-93. Epub 2004/12/14. PubMed PMID: 15593052.

41. Lim CS, Kim YS, Chae DW, Ahn C, Han JS, Kim S, et al. Association of C-509T and T869C polymorphisms of transforming growth factor-beta1 gene with susceptibility to and progression of IgA nephropathy. *Clin Nephrol* (2005) 63(2):61-7. Epub 2005/02/26. doi: 10.5414/cnp63061. PubMed PMID: 15730046.

42. Vuong MT, Lundberg S, Gunnarsson I, Wramner L, Seddighzadeh M, Hahn-Zoric M, et al. Genetic variation in the transforming growth factor-beta1 gene is associated with susceptibility to IgA nephropathy. *Nephrol Dial Transplant* (2009) 24(10):3061-7. Epub 2009/03/05. doi: 10.1093/ndt/gfp079. PubMed PMID: 19258388; PubMed Central PMCID: PMCPMC2747497.

43. Brezzi B, Del Prete D, Lupo A, Magistroni R, Gomez-Lira M, Bernich P, et al. Primary IgA nephropathy is more severe in TGF-beta1 high secretor patients. *J Nephrol* (2009) 22(6):747-59. Epub 2009/12/08. PubMed PMID: 19967654.

44. Suh JS, Hahn WH, Lee JS, Park HJ, Kim MJ, Kang SW, et al. Coding polymorphisms of bone morphogenetic protein 2 contribute to the development of childhood IgA nephropathy. *Exp Ther Med* (2011) 2(2):337-41. Epub 2011/03/01. doi: 10.3892/etm.2011.195. PubMed PMID: 22977507; PubMed Central PMCID: PMCPMC3440631.

45. Suh JS, Cho SH, Chung JH, Moon A, Park YK, Cho BS. A polymorphism of interleukin-22 receptor alpha-1 is associated with the development of childhood IgA nephropathy. *J Interferon Cytokine Res* (2013) 33(10):571-7. Epub 2013/05/11. doi: 10.1089/jir.2012.0097. PubMed PMID: 23659670; PubMed Central PMCID: PMCPMC3793651.

**Supplementary Table 2**

| Study  number | Author | Year | Ethnicity | Country | PMID | Number | Selection criteria | Number | Selection criteria | SNP | PHWE | Allele model OR | 0.95_LCI | 0.95_UCI | P |
| --- | --- | --- | --- | --- | --- | --- | --- | --- | --- | --- | --- | --- | --- | --- | --- |
| 21 | Li GS | 2007 | Han Chinese | China | [17228361](https://www.ncbi.nlm.nih.gov/pubmed/31857673) | 670 | IgAN | 494 | healthy individuals | C1GALT1 rs5882115 | 0.634 | 0.679 | 0.522 | 0.884 | 0.004 |
| 1 | Shi D | 2020 | Han Chinese | China | [31857673](https://www.ncbi.nlm.nih.gov/pubmed/31857673) | 1399 | IgAN | 1442 | healthy individuals | CFB rs549182 | 0.140 | 1.330 | 1.101 | 1.605 | 0.003 |
| 1 | Shi D | 2020 | Han Chinese | China | [31857673](https://www.ncbi.nlm.nih.gov/pubmed/31857673) | 1975 | IgAN | 2004 | healthy individuals | CFB rs4151657 | 0.460 | 1.154 | 1.050 | 1.268 | 0.003 |
| 1 | Shi D | 2020 | Han Chinese | China | [31857673](https://www.ncbi.nlm.nih.gov/pubmed/31857673) | 1983 | IgAN | 2004 | healthy individuals | CFB rs549182 | 0.740 | 1.190 | 1.016 | 1.395 | 0.031 |
| 1 | Shi D | 2020 | Han Chinese | China | [31857673](https://www.ncbi.nlm.nih.gov/pubmed/31857673) | 1399 | IgAN | 1442 | healthy individuals | CFB rs4151657 | 0.340 | 1.131 | 1.012 | 1.265 | 0.031 |
| 1 | Shi D | 2020 | Han Chinese | China | [31857673](https://www.ncbi.nlm.nih.gov/pubmed/31857673) | 580 | IgAN | 562 | healthy individuals | CFB rs4151657 | 0.900 | 1.212 | 1.016 | 1.446 | 0.033 |
| 12 | Kim HJ | 2011 | Korean pediatric patients | Korea | [21677403](https://www.ncbi.nlm.nih.gov/pubmed/31857673) | 172 | IgAN | 399 | healthy individuals | CTLA4 rs231777 | 0.282 | 1.973 | 1.045 | 3.725 | 0.036 |
| 18 | Jacob M | 2018 | Caucasians | Germany | [29539619](https://www.ncbi.nlm.nih.gov/pubmed/31857673) | 455 | IgAN | 252 | healthy individuals | CTLA4 rs5742909 | 0.153 | 1.573 | 1.016 | 2.436 | 0.042 |
| 12 | Kim HJ | 2011 | Korean pediatric patients | Korea | [21677403](https://www.ncbi.nlm.nih.gov/pubmed/31857673) | 172 | IgAN | 399 | healthy individuals | CTLA4 rs231779 | 0.683 | 1.588 | 0.999 | 2.524 | 0.05 |
| 14 | Suh JS | 2011 | Korean pediatric patients | Korea | [21214373](https://www.ncbi.nlm.nih.gov/pubmed/31857673) | 192 | IgAN | 397 | healthy individuals | CXCL8 rs4073 | 0.911 | 0.741 | 0.561 | 0.978 | 0.034 |
| 29 | Gao J | 2017 | Han Chinese | China | [28946141](https://www.ncbi.nlm.nih.gov/pubmed/31857673) | 351 | IgAN | 310 | healthy individuals | Enos rs1799983 | 0.857 | 0.671 | 0.469 | 0.959 | 0.029 |
| 2 | Zhou XJ | 2013 | Han Chinese | China | [23593433](https://www.ncbi.nlm.nih.gov/pubmed/31857673) | 1,194 | IgAN | 900 | healthy individuals | FCRLA rs1954174 | 0.434 | 1.158 | 1.022 | 1.312 | 0.021 |
| 2 | Zhou XJ | 2013 | Han Chinese | China | [23593433](https://www.ncbi.nlm.nih.gov/pubmed/31857673) | 1,194 | IgAN | 900 | healthy individuals | FCRLA rs2333749 | 0.510 | 0.854 | 0.740 | 0.986 | 0.031 |
| 2 | Zhou XJ | 2013 | Han Chinese | China | [23593433](https://www.ncbi.nlm.nih.gov/pubmed/31857673) | 1,194 | IgAN | 900 | healthy individuals | FCRLA rs1954173 | 0.413 | 0.841 | 0.715 | 0.988 | 0.036 |
| 2 | Zhou XJ | 2013 | Han Chinese | China | [23593433](https://www.ncbi.nlm.nih.gov/pubmed/31857673) | 1,194 | IgAN | 900 | healthy individuals | FCRLB rs4657093 | 0.960 | 0.768 | 0.649 | 0.909 | 0.002 |
| 2 | Zhou XJ | 2013 | Han Chinese | China | [23593433](https://www.ncbi.nlm.nih.gov/pubmed/31857673) | 1,194 | IgAN | 900 | healthy individuals | FCRLB rs1417582 | 0.804 | 0.815 | 0.693 | 0.957 | 0.013 |
| 2 | Zhou XJ | 2013 | Han Chinese | China | [23593433](https://www.ncbi.nlm.nih.gov/pubmed/31857673) | 1,194 | IgAN | 900 | healthy individuals | FCRLB rs1891020 | 0.834 | 0.817 | 0.695 | 0.960 | 0.014 |
| 2 | Zhou XJ | 2013 | Han Chinese | China | [23593433](https://www.ncbi.nlm.nih.gov/pubmed/31857673) | 1,194 | IgAN | 900 | healthy individuals | FCRLB rs12079477 | 0.869 | 0.866 | 0.766 | 0.978 | 0.021 |
| 34 | Feng Y | 2019 | Han Chinese | China | [30928649](https://www.ncbi.nlm.nih.gov/pubmed/31857673) | 357 | IgAN | 384 | healthy individuals | GNG2 rs3204008 | 0.824 | 1.332 | 1.042 | 1.702 | 0.022 |
| 17 | Yang B | 2018 | Han Chinese | China | [29467950](https://www.ncbi.nlm.nih.gov/pubmed/31857673) | 140 | IgAN | 490 | healthy individuals | HLA-DP rs9277535 | 0.990 | 1.958 | 1.497 | 2.560 | 0 |
| 17 | Yang B | 2018 | Han Chinese | China | 29467950 | 140 | IgAN | 490 | healthy individuals | HLA-DP rs3077 | 0.930 | 1.601 | 1.221 | 2.099 | 0.001 |
| 12 | Kim HJ | 2011 | Korean pediatric patients | Korea | [21677403](https://www.ncbi.nlm.nih.gov/pubmed/23593433) | 172 | IgAN | 399 | healthy individuals | ICOS rs4270326 | 0.756 | 2.082 | 1.079 | 4.018 | 0.029 |
| 12 | Kim HJ | 2011 | Korean pediatric patients | Korea | [21677403](https://www.ncbi.nlm.nih.gov/pubmed/23593433) | 172 | IgAN | 399 | healthy individuals | ICOS rs4404254 | 0.338 | 1.919 | 1.029 | 3.579 | 0.04 |
| 12 | Kim HJ | 2011 | Korean pediatric patients | Korea | [21677403](https://www.ncbi.nlm.nih.gov/pubmed/23593433) | 172 | IgAN | 399 | healthy individuals | ICOS rs10183087 | 0.400 | 1.891 | 1.026 | 3.485 | 0.041 |
| 12 | Kim HJ | 2011 | Korean pediatric patients | Korea | [21677403](https://www.ncbi.nlm.nih.gov/pubmed/23593433) | 172 | IgAN | 399 | healthy individuals | ICOS rs11571314 | 0.400 | 1.891 | 1.026 | 3.485 | 0.041 |
| 12 | Kim HJ | 2011 | Korean pediatric patients | Korea | [21677403](https://www.ncbi.nlm.nih.gov/pubmed/23593433) | 172 | IgAN | 399 | healthy individuals | ICOS rs1559931 | 0.400 | 1.891 | 1.026 | 3.485 | 0.041 |
| 8 | [Gao J](https://pubmed.ncbi.nlm.nih.gov/?sort=date&term=Gao+J&cauthor_id=28391282) | 2018 | Han Chinese | China | [28391282](https://www.ncbi.nlm.nih.gov/pubmed/23593433) | 351 | IgAN | 310 | healthy individuals | IFN-γ rs430561 | 0.250 | 0.589 | 0.353 | 0.982 | 0.042 |
| 10 | Gao J | 2017 | Han Chinese | China | [28359052](https://www.ncbi.nlm.nih.gov/pubmed/23593433) | 351 | IgAN | 310 | healthy individuals | IL-10 rs1800872 | 0.123 | 1.293 | 1.028 | 1.626 | 0.028 |
| 10 | Gao J | 2017 | Han Chinese | China | [28359052](https://www.ncbi.nlm.nih.gov/pubmed/23593433) | 351 | IgAN | 310 | healthy individuals | IL-10 rs1800871 | 0.139 | 1.292 | 1.028 | 1.624 | 0.028 |
| 22 | Hahn WH | 2010 | Korean pediatric patients | Korea | [19280228](https://www.ncbi.nlm.nih.gov/pubmed/23593433) | 182 | IgAN | 500 | healthy individuals | IL1RN rs439154 | 0.422 | 0.712 | 0.549 | 0.925 | 0.011 |
| 22 | Hahn WH | 2010 | Korean pediatric patients | Korea | [19280228](https://www.ncbi.nlm.nih.gov/pubmed/23593433) | 182 | IgAN | 500 | healthy individuals | IL1RN rs928940 | 0.200 | 0.752 | 0.587 | 0.964 | 0.024 |
| 22 | Hahn WH | 2010 | Korean pediatric patients | Korea | [19280228](https://www.ncbi.nlm.nih.gov/pubmed/23593433) | 182 | IgAN | 500 | healthy individuals | IL1RN rs315951 | 0.259 | 0.753 | 0.585 | 0.968 | 0.027 |
| 23 | Jung HY | 2012 | Korean | Korea | [26889427](https://www.ncbi.nlm.nih.gov/pubmed/23593433) | 69 | IgAN | 146 | healthy individuals | IL-1β rs1946518 | 0.705 | 2.055 | 1.353 | 3.120 | 0.001 |
| 9 | Zhang D | 2017 | Han Chinese | China | [29069743](https://www.ncbi.nlm.nih.gov/pubmed/23593433) | 417 | IgAN | 463 | healthy individuals | IL-1β rs16944 | 0.162 | 1.385 | 1.115 | 1.720 | 0.003 |
| 22 | Hahn WH | 2010 | Korean pediatric patients | Korea | [19280228](https://www.ncbi.nlm.nih.gov/pubmed/23593433) | 182 | IgAN | 500 | healthy individuals | IL-1β rs1143627 | 0.448 | 0.730 | 0.572 | 0.931 | 0.011 |
| 9 | Zhang D | 2017 | Han Chinese | China | [29069743](https://www.ncbi.nlm.nih.gov/pubmed/23593433) | 417 | IgAN | 463 | healthy individuals | IL-1β rs1143627 | 0.160 | 1.285 | 1.035 | 1.595 | 0.023 |
| 22 | Hahn WH | 2010 | Korean pediatric patients | Korea | [19280228](https://www.ncbi.nlm.nih.gov/pubmed/23593433) | 182 | IgAN | 500 | healthy individuals | IL-1β rs3917356 | 0.170 | 1.322 | 1.037 | 1.687 | 0.025 |
| 22 | Hahn WH | 2010 | Korean pediatric patients | Korea | [19280228](https://www.ncbi.nlm.nih.gov/pubmed/23593433) | 182 | IgAN | 500 | healthy individuals | IL-1β rs1143633 | 0.712 | 0.763 | 0.593 | 0.983 | 0.036 |
| 24 | Yang B | 2017 | Han Chinese | China | [27028244](https://www.ncbi.nlm.nih.gov/pubmed/23593433) | 166 | IgAN | 198 | healthy individuals | IL-1β rs1946518 | 0.390 | 1.349 | 1.007 | 1.807 | 0.045 |
| 9 | Zhang D | 2017 | Han Chinese | China | [29069743](https://www.ncbi.nlm.nih.gov/pubmed/23593433) | 417 | IgAN | 463 | healthy individuals | IL-6 rs1800796 | 0.309 | 1.484 | 1.168 | 1.885 | 0.001 |
| 2 | Zhou XJ | 2013 | Han Chinese | China | [23593433](https://www.ncbi.nlm.nih.gov/pubmed/23593433) | 1,194 | IgAN | 900 | healthy individuals | intergenic rs10917750 | 0.808 | 0.795 | 0.676 | 0.934 | 0.005 |
| 2 | Zhou XJ | 2013 | Han Chinese | China | [23593433](https://www.ncbi.nlm.nih.gov/pubmed/23593433) | 1,194 | IgAN | 900 | healthy individuals | intergenic rs4657039 | 0.160 | 0.838 | 0.729 | 0.963 | 0.013 |
| 2 | Zhou XJ | 2013 | Han Chinese | China | [23593433](https://www.ncbi.nlm.nih.gov/pubmed/23593433) | 1,194 | IgAN | 900 | healthy individuals | intergenic rs7549830 | 0.054 | 1.158 | 1.025 | 1.309 | 0.019 |
| 2 | Zhou XJ | 2013 | Han Chinese | China | [23593433](https://www.ncbi.nlm.nih.gov/pubmed/23593433) | 1,194 | IgAN | 900 | healthy individuals | intergenic rs1503813 | 0.320 | 0.840 | 0.720 | 0.979 | 0.026 |
| 2 | Zhou XJ | 2013 | Han Chinese | China | [23593433](https://www.ncbi.nlm.nih.gov/pubmed/23593433) | 1,194 | IgAN | 900 | healthy individuals | intergenic rs10494356 | 0.691 | 1.151 | 1.016 | 1.303 | 0.027 |
| 35 | Shi D | 2020 | Han Chinese | China | [31227791](https://www.ncbi.nlm.nih.gov/pubmed/23593433) | 1000 | IgAN | 1000 | healthy individuals | ITGAX rs11150614 | 0.984 | 0.843 | 0.736 | 0.966 | 0.014 |
| 35 | Shi D | 2020 | Han Chinese | China | [31227791](https://www.ncbi.nlm.nih.gov/pubmed/23593433) | 1000 | IgAN | 1000 | healthy individuals | ITGAX rs7190997 | 0.989 | 0.857 | 0.749 | 0.981 | 0.025 |
| 1 | Shi D | 2020 | Han Chinese | China | [31857673](https://www.ncbi.nlm.nih.gov/pubmed/23593433) | 1399 | IgAN(stage1) | 1442 | healthy individuals | LEMD2 rs751728 | 0.570 | 0.868 | 0.764 | 0.986 | 0.03 |
| 34 | Feng Y | 2019 | Han Chinese | China | [30928649](https://www.ncbi.nlm.nih.gov/pubmed/23593433) | 357 | IgAN | 384 | healthy individuals | NTN4 rs1362970 | 0.250 | 1.535 | 1.156 | 2.036 | 0.003 |
| 34 | Feng Y | 2019 | Han Chinese | China | [30928649](https://www.ncbi.nlm.nih.gov/pubmed/23593433) | 357 | IgAN | 384 | healthy individuals | PHLDB1 rs7389 | 0.603 | 1.284 | 1.013 | 1.627 | 0.039 |
| 17 | Yang B | 2018 | Han Chinese | China | [29467950](https://www.ncbi.nlm.nih.gov/pubmed/23593433) | 140 | IgAN | 490 | healthy individuals | STAT4 rs7574865 | 0.820 | 1.491 | 1.137 | 1.956 | 0.004 |
| 42 | Vuong MT | 2009 | Sweden | Sweden | [19258388](https://www.ncbi.nlm.nih.gov/pubmed/23593433) | 144 | IgAN(male) | 314 | healthy individuals | TGF‐β1 rs1800469 | 0.660 | 0.666 | 0.494 | 0.898 | 0.008 |
| 42 | Vuong MT | 2009 | Sweden | Sweden | [19258388](https://www.ncbi.nlm.nih.gov/pubmed/23593433) | 212 | IgAN(male) | 477 | healthy individuals | TGF‐β1 rs6957 | 0.102 | 0.644 | 0.449 | 0.924 | 0.017 |
| 42 | Vuong MT | 2009 | Sweden | Sweden | [19258388](https://www.ncbi.nlm.nih.gov/pubmed/23593433) | 144 | IgAN(male) | 314 | healthy individuals | TGF‐β1 rs2241715 | 0.645 | 1.425 | 1.056 | 1.924 | 0.021 |
| 42 | Vuong MT | 2009 | Sweden | Sweden | [19258388](https://www.ncbi.nlm.nih.gov/pubmed/23593433) | 143 | IgAN(male) | 319 | healthy individuals | TGF‐β1 rs1982073 | 0.534 | 1.558 | 1.173 | 2.071 | 0.032 |
| 39 | Sato F | 2004 | Japanese | Japan | [15191521](https://www.ncbi.nlm.nih.gov/pubmed/23593433) | 329 | IgAN | 297 | healthy individuals | TGF‐β1 rs1982073 | 0.652 | 1.041 | 0.834 | 1.300 | 0.032 |
| 23 | Jung HY | 2012 | Korean | Korea | [26889427](https://www.ncbi.nlm.nih.gov/pubmed/23593433) | 69 | IgAN | 146 | healthy individuals | TGF‐β1 rs1982073 | 0.099 | 1.355 | 0.901 | 2.036 | 0.047 |
| 6 | Lee JS | 2010 | Korean pediatric patients | Korea | [21108742](https://www.ncbi.nlm.nih.gov/pubmed/23593433) | 190 | IgAN | 283 | healthy individuals | TLR1 rs5743557 | 0.956 | 1.314 | 1.012 | 1.705 | 0.04 |
| 28 | Gao J | 2016 | Han Chinese | China | [27806314](https://www.ncbi.nlm.nih.gov/pubmed/23593433) | 351 | IgAN | 310 | healthy individuals | TLR1 rs4833095 | 0.944 | 1.265 | 1.011 | 1.582 | 0.04 |
| 7 | Park HJ | 2011 | Korean pediatric patients | Korea | [20953797](https://www.ncbi.nlm.nih.gov/pubmed/23593433) | 199 | IgAN | 289 | healthy individuals | TLR10 rs10004195 | 0.323 | 1.393 | 1.078 | 1.800 | 0.011 |
| 33 | Zhong Z | 2017 | Han Chinese | China | [28636766](https://www.ncbi.nlm.nih.gov/pubmed/23593433) | 962 | IgAN | 963 | healthy individuals | TNFSF13 rs3803800 | 0.213 | 0.805 | 0.707 | 0.915 | 0.001 |
| 34 | Feng Y | 2019 | Han Chinese | China | [30928649](https://www.ncbi.nlm.nih.gov/pubmed/23593433) | 357 | IgAN | 384 | healthy individuals | TNS3 rs3750163 | 1.000 | 6.768 | 2.841 | 16.123 | 0 |
| 23 | Jung HY | 2012 | Korean | Korea | [26889427](https://www.ncbi.nlm.nih.gov/pubmed/23593433) | 69 | IgAN | 146 | healthy individuals | VEGF 405 | 0.823 | 1.784 | 1.182 | 2.694 | 0.006 |

**Supplementary Table 3**

| Study number | Author | Year | Ethnicity | Country | PMID | Number | Selection criteria | Number | Selection criteria | SNP | PHWE | Dominant OR | 0.95_LCI | 0.95_UCI | P |
| --- | --- | --- | --- | --- | --- | --- | --- | --- | --- | --- | --- | --- | --- | --- | --- |
| 21 | G-S Li | 2007 | Han Chinese | China | [17228361](https://www.ncbi.nlm.nih.gov/pubmed/31857673) | 670 | IgAN | 494 | healthy individuals | C1GALT1 rs5882115 | 0.634 | 0.68000 | 0.51 | 0.9043255 | 0.008 |
| 1 | Shi D | 2020 | Han Chinese | China | [31857673](https://www.ncbi.nlm.nih.gov/pubmed/31857673) | 580 | IgAN | 562 | healthy individuals | CFB rs4151657 | 0.900 | 1.34790 | 1.07 | 1.702535 | 0.012 |
| 1 | Shi D | 2020 | Han Chinese | China | [31857673](https://www.ncbi.nlm.nih.gov/pubmed/31857673) | 1983 | IgAN | 2004 | healthy individuals | CFB rs549182 | 0.740 | 1.18795 | 1.00 | 1.406844 | 0.046 |
| 1 | Shi D | 2020 | Han Chinese | China | [31857673](https://www.ncbi.nlm.nih.gov/pubmed/31857673) | 1975 | IgAN | 2004 | healthy individuals | CFB rs4151657 | 0.460 | 1.27427 | 1.12 | 1.443817 | 0 |
| 1 | Shi D | 2020 | Han Chinese | China | [31857673](https://www.ncbi.nlm.nih.gov/pubmed/31857673) | 1399 | IgAN | 1442 | healthy individuals | CFB rs4151657 | 0.340 | 1.24527 | 1.07 | 1.443715 | 0.004 |
| 1 | Shi D | 2020 | Han Chinese | China | [31857673](https://www.ncbi.nlm.nih.gov/pubmed/31857673) | 1399 | IgAN | 1442 | healthy individuals | CFB rs549182 | 0.140 | 1.30343 | 1.07 | 1.592712 | 0.01 |
| 12 | Kim HJ | 2011 | Korean pediatric patients | Korea | [21677403](https://www.ncbi.nlm.nih.gov/pubmed/31857673) | 172 | IgAN | 399 | healthy individuals | CTLA4 rs231779 | 0.683 | 1.97284 | 1.06 | 3.668248 | 0.032 |
| 18 | Jacob M | 2018 | Caucasians | Germany | [29539619](https://www.ncbi.nlm.nih.gov/pubmed/31857673) | 455 | IgAN | 252 | healthy individuals | CTLA4 rs5742909 | 0.153 | 1.65739 | 1.03 | 2.654463 | 0.036 |
| 14 | Suh JS | 2011 | Korean pediatric patients | Korea | [21214373](https://www.ncbi.nlm.nih.gov/pubmed/31857673) | 192 | IgAN | 397 | healthy individuals | CXCL8 rs2227543 | 0.790 | 1.64122 | 1.12 | 2.400149 | 0.011 |
| 14 | Suh JS | 2011 | Korean pediatric patients | Korea | [21214373](https://www.ncbi.nlm.nih.gov/pubmed/31857673) | 192 | IgAN | 397 | healthy individuals | CXCL8 rs2227306 | 0.395 | 1.57689 | 1.08 | 2.303144 | 0.018 |
| 2 | Zhou XJ | 2013 | Han Chinese | China | [23593433](https://www.ncbi.nlm.nih.gov/pubmed/31857673) | 1,194 | IgAN | 900 | healthy individuals | FCRLA rs2333749 | 0.510 | 0.82870 | 0.70 | 0.9878526 | 0.036 |
| 2 | Zhou XJ | 2013 | Han Chinese | China | [23593433](https://www.ncbi.nlm.nih.gov/pubmed/31857673) | 1,194 | IgAN | 900 | healthy individuals | FCRLA rs1954173 | 0.413 | 0.81090 | 0.67 | 0.9765775 | 0.027 |
| 2 | Zhou XJ | 2013 | Han Chinese | China | [23593433](https://www.ncbi.nlm.nih.gov/pubmed/31857673) | 1,194 | IgAN | 900 | healthy individuals | FCRLB rs4657093 | 0.960 | 0.75043 | 0.62 | 0.908325 | 0.003 |
| 2 | Zhou XJ | 2013 | Han Chinese | China | [23593433](https://www.ncbi.nlm.nih.gov/pubmed/31857673) | 1,194 | IgAN | 900 | healthy individuals | FCRLB rs1891020 | 0.834 | 0.79251 | 0.66 | 0.9543369 | 0.014 |
| 2 | Zhou XJ | 2013 | Han Chinese | China | [23593433](https://www.ncbi.nlm.nih.gov/pubmed/31857673) | 1,194 | IgAN | 900 | healthy individuals | FCRLB rs1417582 | 0.804 | 0.78954 | 0.66 | 0.9509608 | 0.013 |
| 17 | Yang B | 2018 | Han Chinese | China | [29467950](https://www.ncbi.nlm.nih.gov/pubmed/31857673) | 140 | IgAN | 490 | healthy individuals | HLA-DP rs9277535 | 0.990 | 2.05354 | 1.34 | 3.158399 | 0.001 |
| 12 | Kim HJ | 2011 | Korean pediatric patients | Korea | [21677403](https://www.ncbi.nlm.nih.gov/pubmed/31857673) | 172 | IgAN | 399 | healthy individuals | ICOS rs4270326 | 0.756 | 2.23918 | 1.08 | 4.632189 | 0.03 |
| 12 | Kim HJ | 2011 | Korean pediatric patients | Korea | [21677403](https://www.ncbi.nlm.nih.gov/pubmed/31857673) | 172 | IgAN | 399 | healthy individuals | ICOS rs10183087 | 0.400 | 2.17830 | 1.09 | 4.347564 | 0.027 |
| 12 | Kim HJ | 2011 | Korean pediatric patients | Korea | [21677403](https://www.ncbi.nlm.nih.gov/pubmed/31857673) | 172 | IgAN | 399 | healthy individuals | ICOS rs11571314 | 0.400 | 2.17830 | 1.09 | 4.347564 | 0.027 |
| 12 | Kim HJ | 2011 | Korean pediatric patients | Korea | [21677403](https://www.ncbi.nlm.nih.gov/pubmed/31857673) | 172 | IgAN | 399 | healthy individuals | ICOS rs1559931 | 0.400 | 2.17830 | 1.09 | 4.347564 | 0.027 |
| 12 | Kim HJ | 2011 | Korean pediatric patients | Korea | 21677403 | 172 | IgAN | 399 | healthy individuals | ICOS rs4404254 | 0.338 | 2.21088 | 1.09 | 4.467172 | 0.027 |
| 8 | [Gao J](https://pubmed.ncbi.nlm.nih.gov/?sort=date&term=Gao+J&cauthor_id=28391282) | 2018 | Han Chinese | China | [28391282](https://www.ncbi.nlm.nih.gov/pubmed/28391282) | 351 | IgAN | 310 | healthy individuals | IFN-γ rs430561 | 0.250 | 0.57263 | 0.34 | 0.9672329 | 0.037 |
| 10 | Jie Gao | 2017 | Han Chinese | China | [28359052](https://www.ncbi.nlm.nih.gov/pubmed/23593433) | 351 | IgAN | 310 | healthy individuals | IL-10 rs1800871 | 0.139 | 1.48487 | 1.09 | 2.022402 | 0.012 |
| 10 | Jie Gao | 2017 | Han Chinese | China | [28359052](https://www.ncbi.nlm.nih.gov/pubmed/23593433) | 351 | IgAN | 310 | healthy individuals | IL-10 rs1800872 | 0.123 | 1.48731 | 1.09 | 2.026514 | 0.012 |
| 22 | Hahn WH | 2010 | Korean pediatric patients | Korea | [19280228](https://www.ncbi.nlm.nih.gov/pubmed/23593433) | 182 | IgAN | 500 | healthy individuals | IL1RN rs439154 | 0.422 | 0.62568 | 0.44 | 0.8848362 | 0.008 |
| 9 | Zhang D | 2017 | Han Chinese | China | [29069743](https://www.ncbi.nlm.nih.gov/pubmed/23593433) | 417 | IgAN | 463 | healthy individuals | IL-1β rs16944 | 0.162 | 0.64107 | 0.44 | 0.9283985 | 0.019 |
| 22 | Hahn WH | 2010 | Korean pediatric patients | Korea | [19280228](https://www.ncbi.nlm.nih.gov/pubmed/23593433) | 182 | IgAN | 500 | healthy individuals | IL-1β rs1143633 | 0.712 | 1.44564 | 1.04 | 2.000897 | 0.026 |
| 22 | Hahn WH | 2010 | Korean pediatric patients | Korea | [19280228](https://www.ncbi.nlm.nih.gov/pubmed/23593433) | 182 | IgAN | 500 | healthy individuals | IL-1β rs1143627 | 0.448 | 0.66882 | 0.47 | 0.9427977 | 0.022 |
| 38 | Liu XQ | 2008 | Caucasians | Canada | [18256355](https://www.ncbi.nlm.nih.gov/pubmed/23593433) | 271 | IgAN(St. Etienne) | 205 | healthy individuals | IL4R rs1805015 | 0.050 | 0.43366 | 0.28 | 0.6642273 | 0 |
| 38 | Liu XQ | 2008 | Caucasians | Canada | [18256355](https://www.ncbi.nlm.nih.gov/pubmed/23593433) | 271 | IgAN(St. Etienne) | 205 | healthy individuals | IL5RA rs340833 | 0.918 | 1.78266 | 1.18 | 2.69622 | 0.006 |
| 38 | Liu XQ | 2008 | Caucasians | Canada | [18256354](https://www.ncbi.nlm.nih.gov/pubmed/23593433) | 255 | IgAN(Toronto) | 187 | healthy individuals | IL5RA rs340833 | 0.084 | 0.62327 | 0.40 | 0.983457 | 0.042 |
| 9 | Zhang D | 2017 | Han Chinese | China | [29069743](https://www.ncbi.nlm.nih.gov/pubmed/23593433) | 417 | IgAN | 463 | healthy individuals | IL-6 rs1800796 | 0.309 | 1.49274 | 1.10 | 2.026464 | 0.01 |
| 2 | Zhou XJ | 2013 | Han Chinese | China | [23593433](https://www.ncbi.nlm.nih.gov/pubmed/23593433) | 1,194 | IgAN | 900 | healthy individuals | intergenic rs10917750 | 0.808 | 0.76460 | 0.63 | 0.9211601 | 0.005 |
| 2 | Zhou XJ | 2013 | Han Chinese | China | [23593433](https://www.ncbi.nlm.nih.gov/pubmed/23593433) | 1,194 | IgAN | 900 | healthy individuals | intergenic rs1503813 | 0.320 | 0.81119 | 0.68 | 0.9715359 | 0.023 |
| 2 | Zhou XJ | 2013 | Han Chinese | China | [23593433](https://www.ncbi.nlm.nih.gov/pubmed/23593433) | 1,194 | IgAN | 900 | healthy individuals | intergenic rs4657039 | 0.160 | 0.77764 | 0.65 | 0.925347 | 0.005 |
| 35 | Shi D | 2020 | Han Chinese | China | [31227791](https://www.ncbi.nlm.nih.gov/pubmed/23593433) | 1000 | IgAN | 1000 | healthy individuals | ITGAM rs4597342 | 0.520 | 0.81126 | 0.68 | 0.9679836 | 0.02 |
| 35 | Shi D | 2020 | Han Chinese | China | [31227791](https://www.ncbi.nlm.nih.gov/pubmed/23593433) | 1000 | IgAN | 1000 | healthy individuals | ITGAX rs7190997 | 0.989 | 0.82213 | 0.69 | 0.9807116 | 0.03 |
| 35 | Shi D | 2020 | Han Chinese | China | [31227791](https://www.ncbi.nlm.nih.gov/pubmed/23593433) | 1000 | IgAN | 1000 | healthy individuals | ITGAX rs11150614 | 0.984 | 0.80465 | 0.67 | 0.9603661 | 0.016 |
| 35 | Shi D | 2020 | Han Chinese | China | [31227791](https://www.ncbi.nlm.nih.gov/pubmed/23593433) | 1000 | IgAN | 1000 | healthy individuals | ITGAX rs1140195 | 0.623 | 0.82623 | 0.69 | 0.9870476 | 0.035 |
| 2 | Zhou XJ | 2013 | Han Chinese | China | [23593433](https://www.ncbi.nlm.nih.gov/pubmed/23593433) | 1,194 | IgAN | 900 | healthy individuals | NA rs6696854 | 0.840 | 0.83275 | 0.70 | 0.9903014 | 0.038 |
| 2 | Zhou XJ | 2013 | Han Chinese | China | [23593433](https://www.ncbi.nlm.nih.gov/pubmed/23593433) | 1,194 | IgAN | 900 | healthy individuals | NA rs10800309 | 0.548 | 0.83572 | 0.70 | 0.9939791 | 0.043 |
| 2 | Zhou XJ | 2013 | Han Chinese | China | [23593433](https://www.ncbi.nlm.nih.gov/pubmed/23593433) | 1,194 | IgAN | 900 | healthy individuals | NA rs12749327 | 0.517 | 1.39554 | 1.02 | 1.914907 | 0.039 |
| 2 | Zhou XJ | 2013 | Han Chinese | China | [23593433](https://www.ncbi.nlm.nih.gov/pubmed/23593433) | 1,194 | IgAN | 900 | healthy individuals | NA rs905589 | 0.264 | 0.82830 | 0.69 | 0.9914477 | 0.04 |
| 34 | Feng Y | 2019 | Han Chinese | China | [30928649](https://www.ncbi.nlm.nih.gov/pubmed/23593433) | 357 | IgAN | 384 | healthy individuals | NTN4 rs1362970 | 0.250 | 1.52835 | 1.08 | 2.156891 | 0.016 |
| 37 | Fu D | 2020 | Han Chinese | China | [32747022](https://www.ncbi.nlm.nih.gov/pubmed/23593433) | 960 | IgAN | 956 | healthy individuals | ST6GAL1 rs12054151 | 0.767 | 1.44441 | 1.11 | 1.875033 | 0.006 |
| 37 | Fu D | 2020 | Han Chinese | China | [32747022](https://www.ncbi.nlm.nih.gov/pubmed/23593433) | 960 | IgAN | 956 | healthy individuals | ST6GAL1 rs2239611 | 0.689 | 1.55012 | 1.24 | 1.944657 | 0 |
| 37 | Fu D | 2020 | Han Chinese | China | [32747022](https://www.ncbi.nlm.nih.gov/pubmed/23593433) | 960 | IgAN | 956 | healthy individuals | ST6GAL1 rs1990677 | 0.499 | 1.52527 | 1.04 | 2.235852 | 0.031 |
| 37 | Fu D | 2020 | Han Chinese | China | [32747022](https://www.ncbi.nlm.nih.gov/pubmed/23593433) | 960 | IgAN | 956 | healthy individuals | ST6GAL1 rs4686838 | 0.379 | 0.36516 | 0.30 | 0.4431336 | 0 |
| 37 | Fu D | 2020 | Han Chinese | China | [32747022](https://www.ncbi.nlm.nih.gov/pubmed/23593433) | 960 | IgAN | 956 | healthy individuals | ST6GAL1 rs2284750 | 0.377 | 0.54145 | 0.45 | 0.657507 | 0 |
| 37 | Fu D | 2020 | Han Chinese | China | [32747022](https://www.ncbi.nlm.nih.gov/pubmed/23593433) | 960 | IgAN | 956 | healthy individuals | ST6GAL1 rs6784233 | 0.157 | 1.38865 | 1.09 | 1.763951 | 0.007 |
| 37 | Fu D | 2020 | Han Chinese | China | [32747022](https://www.ncbi.nlm.nih.gov/pubmed/23593433) | 960 | IgAN | 956 | healthy individuals | ST6GAL1 rs7634389 | 0.083 | 1.26157 | 1.02 | 1.555686 | 0.03 |
| 36 | Lu C | 2015 | Uyghur Chinese | China | [26136946](https://www.ncbi.nlm.nih.gov/pubmed/23593433) | 180 | IgAN | 180 | healthy individuals | ST6GALNAC2 rs3840858 | 0.7015 | 3.67568 | 1.75 | 7.73074 | 0.001 |
| 39 | Sato F | 2004 | Japanese | Japan | [15191521](https://www.ncbi.nlm.nih.gov/pubmed/23593433) | 329 | IgAN | 297 | healthy individuals | TGF‐β1 rs1982073 | 0.652 | 1.04169 | 0.73 | 1.485997 | 0.036 |
| 42 | Vuong MT | 2009 | Sweden | Sweden | [19258388](https://www.ncbi.nlm.nih.gov/pubmed/23593433) | 144 | IgAN(male) | 314 | healthy individuals | TGF‐β1 rs2241715 | 0.645 | 1.73093 | 1.16 | 2.578755 | 0.007 |
| 42 | Vuong MT | 2009 | Sweden | Sweden | [19258388](https://www.ncbi.nlm.nih.gov/pubmed/23593433) | 143 | IgAN(male) | 319 | healthy individuals | TGF‐β1 rs1982073 | 0.534 | 1.88571 | 1.23 | 2.893031 | 0.036 |
| 42 | Vuong MT | 2009 | Sweden | Sweden | [19258388](https://www.ncbi.nlm.nih.gov/pubmed/23593433) | 145 | IgAN(male) | 315 | healthy individuals | TGF‐β1 rs180047 | 0.146 | 0.04851 | 0.00 | 0.9071811 | 0.043 |
| 42 | Vuong MT | 2009 | Sweden | Sweden | [19258388](https://www.ncbi.nlm.nih.gov/pubmed/23593433) | 212 | IgAN(male) | 477 | healthy individuals | TGF‐β1 rs6957 | 0.102 | 0.14469 | 0.04 | 0.542827 | 0.004 |
| 6 | Lee JS | 2010 | Korean pediatric patients | Korea | [21108742](https://www.ncbi.nlm.nih.gov/pubmed/23593433) | 190 | IgAN | 283 | healthy individuals | TLR1 rs5743557 | 0.956 | 1.52226 | 1.00 | 2.317318 | 0.05 |
| 6 | Lee JS | 2010 | Korean pediatric patients | Korea | [21108742](https://www.ncbi.nlm.nih.gov/pubmed/23593433) | 190 | IgAN | 283 | healthy individuals | TLR1 rs4833095 | 0.083 | 1.96849 | 1.16 | 3.343953 | 0.012 |
| 7 | Park HJ | 2011 | Korean pediatric patients | Korea | [20953797](https://www.ncbi.nlm.nih.gov/pubmed/23593433) | 199 | IgAN | 289 | healthy individuals | TLR10 rs10004195 | 0.323 | 1.98017 | 1.28 | 3.060338 | 0.002 |
| 38 | Liu XQ | 2008 | Caucasians | Canada | [18256355](https://www.ncbi.nlm.nih.gov/pubmed/23593433) | 271 | IgAN(St. Etienne) | 205 | healthy individuals | TNFRSF6B rs1291205 | 0.965 | 0.44846 | 0.31 | 0.6547217 | 0 |
| 38 | Liu XQ | 2008 | Caucasians | Canada | [18256355](https://www.ncbi.nlm.nih.gov/pubmed/23593433) | 271 | IgAN(St. Etienne) | 205 | healthy individuals | TNFRSF6B rs1291206 | 0.958 | 0.45731 | 0.31 | 0.6676927 | 0 |
| 38 | Liu XQ | 2008 | Caucasians | Canada | [18256355](https://www.ncbi.nlm.nih.gov/pubmed/23593433) | 271 | IgAN(St. Etienne) | 205 | healthy individuals | TNFRSF6B rs3208008 | 0.881 | 0.46880 | 0.32 | 0.6846247 | 0 |
| 33 | Zhong Z | 2017 | Han Chinese | China | [28636766](https://www.ncbi.nlm.nih.gov/pubmed/23593433) | 962 | IgAN | 963 | healthy individuals | TNFSF13 rs3803800 | 0.213 | 0.65110 | 0.51 | 0.8314157 | 0.001 |
| 34 | Feng Y | 2019 | Han Chinese | China | [30928649](https://www.ncbi.nlm.nih.gov/pubmed/23593433) | 357 | IgAN | 384 | healthy individuals | TNS3 rs3750163 | 1.000 | 9.83621 | 3.45 | 28.01009 | 0 |

**Supplementary table 4**

| Study number | 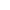   \| author \| \| --- \| | year | ethnicity | country | PMID | SNP | PHWE | Overdominant(OR) | 0.95_LCI | 0.95_UCI | P |
| --- | --- | --- | --- | --- | --- | --- | --- | --- | --- | --- | --- | --- |
| 21 | Li GS | 2007 | Han Chinese | China | [17228361](https://www.ncbi.nlm.nih.gov/pubmed/31857673) | C1GALT1 rs5882115 | 0.634 | 1.387428 | 1.039074 | 1.852571 | 0.026 |
| 1 | Shi D | 2020 | Han Chinese | China | [31857673](https://www.ncbi.nlm.nih.gov/pubmed/31857673) | CFB rs4151657 | 0.460 | 0.7895995 | 0.6966085 | 0.895004 | 0 |
| 1 | Shi D | 2020 | Han Chinese | China | [31857673](https://www.ncbi.nlm.nih.gov/pubmed/31857673) | CFB rs4151657 | 0.340 | 0.7982231 | 0.6880699 | 0.9260107 | 0.003 |
| 1 | Shi D | 2020 | Han Chinese | China | [31857673](https://www.ncbi.nlm.nih.gov/pubmed/31857673) | CFB rs4151657 | 0.900 | 0.7702989 | 0.6098155 | 0.9730163 | 0.029 |
| 1 | Shi D | 2020 | Han Chinese | China | [31857673](https://www.ncbi.nlm.nih.gov/pubmed/31857673) | CFB rs549182 | 0.140 | 0.807918 | 0.659098 | 0.9903407 | 0.04 |
| 14 | Suh JS | 2011 | Korean pediatric patients | Korea | [21214373](https://www.ncbi.nlm.nih.gov/pubmed/31857673) | CXCL8 rs2227543 | 0.790 | 0.5917547 | 0.4061796 | 0.862115 | 0.006 |
| 14 | Suh JS | 2011 | Korean pediatric patients | Korea | [21214373](https://www.ncbi.nlm.nih.gov/pubmed/31857673) | CXCL8 rs2227306 | 0.395 | 0.6024845 | 0.4115853 | 0.8819255 | 0.009 |
| 14 | Suh JS | 2011 | Korean pediatric patients | Korea | [21214373](https://www.ncbi.nlm.nih.gov/pubmed/31857673) | CXCL8 rs4073 | 0.911 | 0.6470588 | 0.4445677 | 0.9417804 | 0.023 |
| 2 | Zhou XJ | 2013 | Han Chinese | China | [23593433](https://www.ncbi.nlm.nih.gov/pubmed/31857673) | FCRLA rs1954173 | 0.413 | 1.222685 | 1.010304 | 1.479712 | 0.039 |
| 2 | Zhou XJ | 2013 | Han Chinese | China | [23593433](https://www.ncbi.nlm.nih.gov/pubmed/31857673) | FCRLB rs4657093 | 0.960 | 1.281395 | 1.053119 | 1.559153 | 0.013 |
| 2 | Zhou XJ | 2013 | Han Chinese | China | [23593433](https://www.ncbi.nlm.nih.gov/pubmed/31857673) | FCRLB rs1417582 | 0.804 | 1.236373 | 1.021057 | 1.497095 | 0.03 |
| 2 | Zhou XJ | 2013 | Han Chinese | China | [23593433](https://www.ncbi.nlm.nih.gov/pubmed/31857673) | FCRLB rs1891020 | 0.834 | 1.232054 | 1.017676 | 1.491591 | 0.032 |
| 17 | Yang B | 2018 | Han Chinese | China | [29467950](https://www.ncbi.nlm.nih.gov/pubmed/31857673) | HLA-DP rs3077 | 0.930 | 4.617284 | 2.809256 | 7.588952 | 0 |
| 12 | Kim HJ | 2011 | Korean pediatric patients | Korea | [21677403](https://www.ncbi.nlm.nih.gov/pubmed/31857673) | ICOS rs10183087 | 0.400 | 0.4515625 | 0.2211878 | 0.9218802 | 0.029 |
| 12 | Kim HJ | 2011 | Korean pediatric patients | Korea | [21677403](https://www.ncbi.nlm.nih.gov/pubmed/31857673) | ICOS rs11571314 | 0.400 | 0.4515625 | 0.2211878 | 0.9218802 | 0.029 |
| 12 | Kim HJ | 2011 | Korean pediatric patients | Korea | [21677403](https://www.ncbi.nlm.nih.gov/pubmed/31857673) | ICOS rs1559931 | 0.400 | 0.4515625 | 0.2211878 | 0.9218802 | 0.029 |
| 12 | Kim HJ | 2011 | Korean pediatric patients | Korea | [21677403](https://www.ncbi.nlm.nih.gov/pubmed/31857673) | ICOS rs4404254 | 0.338 | 0.4434783 | 0.2140752 | 0.9187097 | 0.029 |
| 12 | Kim HJ | 2011 | Korean pediatric patients | Korea | [21677403](https://www.ncbi.nlm.nih.gov/pubmed/31857673) | ICOS rs4270326 | 0.756 | 0.4703833 | 0.2230102 | 0.9921539 | 0.048 |
| 8 | [Gao J](https://pubmed.ncbi.nlm.nih.gov/?sort=date&term=Gao+J&cauthor_id=28391282) | 2018 | Han Chinese | China | [28391282](https://www.ncbi.nlm.nih.gov/pubmed/28391282) | IFN-γ rs430561 | 0.250 | 1.746324 | 1.033877 | 2.949718 | 0.037 |
| 38 | Liu XQ | 2008 | Caucasians | Canada | [18256355](https://www.ncbi.nlm.nih.gov/pubmed/31857673) | IGAN1 rs1342646 | 0.059 | 1.557687 | 1.080724 | 2.245152 | 0.017 |
| 10 | Gao J | 2017 | Han Chinese | China | 28359052 | IL-10 rs1800872 | 0.123 | 0.7216312 | 0.5291697 | 0.9840918 | 0.039 |
| 10 | Gao J | 2017 | Han Chinese | China | [28359052](https://www.ncbi.nlm.nih.gov/pubmed/23593433) | IL-10 rs1800871 | 0.139 | 0.723212 | 0.5306717 | 0.9856104 | 0.04 |
| 22 | Hahn WH | 2010 | Korean pediatric patients | Korea | [19280228](https://www.ncbi.nlm.nih.gov/pubmed/23593433) | IL-1β rs1143633 | 0.712 | 1.502286 | 1.060556 | 2.127999 | 0.022 |
| 24 | Yang B | 2017 | Han Chinese | China | [27028244](https://www.ncbi.nlm.nih.gov/pubmed/23593433) | IL-1β rs1946518 | 0.390 | 1.533333 | 1.00525 | 2.338833 | 0.047 |
| 45 | Suh JS | 2013 | Korean pediatric patients | Korea | [23659670](https://www.ncbi.nlm.nih.gov/pubmed/23593433) | IL22R1 rs3795299 | 0.208 | 0.6611372 | 0.4572847 | 0.9558648 | 0.028 |
| 38 | Liu XQ | 2008 | Caucasians | Canada | [18256355](https://www.ncbi.nlm.nih.gov/pubmed/23593433) | IL4R rs1805015 | 0.050 | 2.44964 | 1.58403 | 3.788273 | 0 |
| 2 | Zhou XJ | 2013 | Han Chinese | China | [23593433](https://www.ncbi.nlm.nih.gov/pubmed/23593433) | intergenic rs1503813 | 0.320 | 1.206449 | 1.002794 | 1.451463 | 0.047 |
| 2 | Zhou XJ | 2013 | Han Chinese | China | [23593433](https://www.ncbi.nlm.nih.gov/pubmed/23593433) | intergenic rs4657039 | 0.160 | 1.268076 | 1.062073 | 1.514036 | 0.009 |
| 2 | Zhou XJ | 2013 | Han Chinese | China | [23593433](https://www.ncbi.nlm.nih.gov/pubmed/23593433) | intergenic rs10917750 | 0.808 | 1.280086 | 1.056732 | 1.55065 | 0.012 |
| 35 | Shi D | 2020 | Han Chinese | China | [31227791](https://www.ncbi.nlm.nih.gov/pubmed/23593433) | ITGAM rs4597342 | 0.520 | 1.215618 | 1.016807 | 1.4533 | 0.032 |
| 35 | Shi D | 2020 | Han Chinese | China | [31227791](https://www.ncbi.nlm.nih.gov/pubmed/23593433) | ITGAX rs11150619 | 0.152 | 1.348561 | 1.12236 | 1.620352 | 0.001 |
| 2 | Zhou XJ | 2013 | Han Chinese | China | [23593433](https://www.ncbi.nlm.nih.gov/pubmed/23593433) | NA rs12749327 | 0.517 | 0.6994809 | 0.5080507 | 0.9630406 | 0.028 |
| 2 | Zhou XJ | 2013 | Han Chinese | China | [23593433](https://www.ncbi.nlm.nih.gov/pubmed/23593433) | NA rs10800309 | 0.548 | 1.193672 | 1.000124 | 1.424677 | 0.05 |
| 37 | Fu D | 2020 | Han Chinese | China | [32747022](https://www.ncbi.nlm.nih.gov/pubmed/23593433) | ST6GAL1 rs4686838 | 0.379 | 1.841069 | 1.490156 | 2.274617 | 0 |
| 37 | Fu D | 2020 | Han Chinese | China | [32747022](https://www.ncbi.nlm.nih.gov/pubmed/23593433) | ST6GAL1 rs2284750 | 0.377 | 1.582382 | 1.274685 | 1.964356 | 0 |
| 37 | Fu D | 2020 | Han Chinese | China | [32747022](https://www.ncbi.nlm.nih.gov/pubmed/23593433) | ST6GAL1 rs2239611 | 0.689 | 0.6653703 | 0.5211227 | 0.8495458 | 0.001 |
| 37 | Fu D | 2020 | Han Chinese | China | [32747022](https://www.ncbi.nlm.nih.gov/pubmed/23593433) | ST6GAL1 rs12054151 | 0.767 | 0.7115116 | 0.538914 | 0.9393867 | 0.016 |
| 37 | Fu D | 2020 | Han Chinese | China | [32747022](https://www.ncbi.nlm.nih.gov/pubmed/23593433) | ST6GAL1 rs1990677 | 0.499 | 0.6568396 | 0.4438072 | 0.9721301 | 0.036 |
| 36 | Lu C | 2015 | Uyghur Chinese | China | [26136946](https://www.ncbi.nlm.nih.gov/pubmed/23593433) | ST6GALNAC2 rs3840858 | 0.7015 | 0.2720588 | 0.1293537 | 0.5721985 | 0.001 |
| 36 | Lu C | 2015 | Uyghur Chinese | China | [26136946](https://www.ncbi.nlm.nih.gov/pubmed/23593433) | ST6GALNAC2 rs23840858 | 0.083 | 0.6428571 | 0.4154673 | 0.9947 | 0.047 |
| 17 | Yang B | 2018 | Han Chinese | China | [29467950](https://www.ncbi.nlm.nih.gov/pubmed/23593433) | STAT4 rs7574865 | 0.820 | 2.377486 | 1.553347 | 3.638879 | 0 |
| 42 | Vuong MT | 2009 | Sweden | Sweden | [19258388](https://www.ncbi.nlm.nih.gov/pubmed/23593433) | TGF‐β1 rs2241715 | 0.645 | 0.6069547 | 0.4080355 | 0.9028478 | 0.014 |
| 38 | Liu XQ | 2008 | Caucasians | Canada | [18256355](https://www.ncbi.nlm.nih.gov/pubmed/23593433) | TNFRSF6B rs1291205 | 0.965 | 1.777923 | 1.209218 | 2.614096 | 0.003 |
| 38 | Liu XQ | 2008 | Caucasians | Canada | [18256355](https://www.ncbi.nlm.nih.gov/pubmed/23593433) | TNFRSF6B rs1291206 | 0.958 | 1.742222 | 1.184463 | 2.562628 | 0.005 |
| 38 | Liu XQ | 2008 | Caucasians | Canada | [18256355](https://www.ncbi.nlm.nih.gov/pubmed/23593433) | TNFRSF6B rs3208008 | 0.881 | 1.698387 | 1.154023 | 2.499533 | 0.007 |
| 34 | Feng Y | 2019 | Han Chinese | China | [30928649](https://www.ncbi.nlm.nih.gov/pubmed/23593433) | TNS3 rs3750163 | 1.000 | 0.0558308 | 0.0132647 | 0.2349903 | 0 |

**Supplementary table5:**

| number | author | year | ethnicity | country | PMID | gene | SNP | PHWE | Homozygote(OR) | 0.95_LCI | 0.95_UCI | P |
| --- | --- | --- | --- | --- | --- | --- | --- | --- | --- | --- | --- | --- |
| 2 | Zhou XJ | 2013 | Chinese | China | [23593433](https://www.ncbi.nlm.nih.gov/pubmed/31857673) | ATF6 | ATF6 rs905594 | 0.140 | 4.034438 | 1.335102 | 12.19134 | 0.013 |
| 44 | Suh JS | 2011 | pediatric patients | Korea | [22977507](https://www.ncbi.nlm.nih.gov/pubmed/31857673) | BMP2 | BMP2 rs235768 | 0.570 | 0.666309 | 0.461289 | 0.96245 | 0.03 |
| 44 | Suh JS | 2011 | pediatric patients | Korea | [22977507](https://www.ncbi.nlm.nih.gov/pubmed/31857673) | BMP2 | BMP2 rs1049007 | 0.000 | 0.085273 | 0.052018 | 0.139787 | 0 |
| 21 | Li GS | 2007 | Chinese | China | [17228361](https://www.ncbi.nlm.nih.gov/pubmed/31857673) | C1GALT1 | C1GALT1 rs5882115 | 0.030 | 0.704507 | 0.547382 | 0.906735 | 0.007 |
| 16 | Zhou XJ | 2021 | Chinese | China | [33462083](https://www.ncbi.nlm.nih.gov/pubmed/31857673) | CCR6 | CCR6 rs3093023 | 0.434 | 1.38404 | 1.064795 | 1.799 | 0.015 |
| 16 | Zhou XJ | 2021 | Chinese | China | [33462083](https://www.ncbi.nlm.nih.gov/pubmed/31857673) | CCR6 | CCR6 rs3093023 | 0.054 | 1.366695 | 1.062454 | 1.758059 | 0.015 |
| 1 | Shi D | 2020 | Chinese | china | [31857673](https://www.ncbi.nlm.nih.gov/pubmed/31857673) | CFB | CFB rs549182 | 0.869 | 0.750804 | 0.588042 | 0.958615 | 0.022 |
| 16 | Zhou XJ | 2021 | Chinese | China | [33462083](https://www.ncbi.nlm.nih.gov/pubmed/31857673) | FBXL21 | FBXL21 rs40986 | 0.691 | 1.339154 | 1.032906 | 1.736203 | 0.028 |
| 16 | Zhou XJ | 2021 | Chinese | China | [33462083](https://www.ncbi.nlm.nih.gov/pubmed/31857673) | FBXL21 | FBXL21 rs40986 | 0.148 | 1.304299 | 1.01389 | 1.67789 | 0.039 |
| 2 | Zhou XJ | 2013 | Chinese | china | [23593433](https://www.ncbi.nlm.nih.gov/pubmed/31857673) | FCRLA | FCRLA rs1954174 | 0.185 | 1.302331 | 1.012772 | 1.674679 | 0.04 |
| 2 | Zhou XJ | 2013 | Chinese | china | [23593433](https://www.ncbi.nlm.nih.gov/pubmed/31857673) | FCRLB | FCRLB rs1891019 | 0.868 | 1.333294 | 1.011311 | 1.75779 | 0.041 |
| 2 | Zhou XJ | 2013 | Chinese | china | [23593433](https://www.ncbi.nlm.nih.gov/pubmed/31857673) | FCRLB | FCRLB rs12079477 | 0.956 | 1.728512 | 1.016313 | 2.9398 | 0.043 |
| 17 | Yang B | 2018 | Chinese | China | [29467950](https://www.ncbi.nlm.nih.gov/pubmed/31857673) | HLA-DP | HLA-DP rs3077 | 0.323 | 1.976351 | 1.163836 | 3.356113 | 0.012 |
| 17 | Yang B | 2018 | Chinese | China | [29467950](https://www.ncbi.nlm.nih.gov/pubmed/31857673) | HLA-DP | HLA-DP rs9277535 | 0.309 | 1.848155 | 1.164448 | 2.933301 | 0.009 |
| 9 | Zhang D | 2017 | Chinese | China | [29069743](https://www.ncbi.nlm.nih.gov/pubmed/31857673) | IL-1β | IL-1β rs16944 | 0.162 | 1.63015 | 1.118994 | 2.374803 | 0.011 |
| 9 | Zhang D | 2017 | Chinese | China | [29069743](https://www.ncbi.nlm.nih.gov/pubmed/31857673) | IL-1β | IL-1β rs1143627 | 0.160 | 1.45714 | 1.000903 | 2.121342 | 0.049 |
| 22 | Hahn WH | 2010 | Korean pediatric patients | Korea | [19280228](https://www.ncbi.nlm.nih.gov/pubmed/31857673) | IL-1β | IL-1β rs1143627 | 0.000 | 1.479574 | 1.179222 | 1.856428 | 0.001 |
| 22 | Hahn WH | 2010 | Korean pediatric patients | Korea | [19280228](https://www.ncbi.nlm.nih.gov/pubmed/31857673) | IL-1β | IL-1β rs3917356 | 0.000 | 1.533347 | 1.176941 | 1.997681 | 0.002 |
| 23 | Jung HY | 2012 | Korean | Korea | [26889427](https://www.ncbi.nlm.nih.gov/pubmed/31857673) | IL-1β | IL-1β rs1946518 | 0.000 | 0.79079 | 0.625237 | 1.00018 | 0.05 |
| 22 | Hahn WH | 2010 | Korean pediatric patients | Korea | [19280228](https://www.ncbi.nlm.nih.gov/pubmed/31857673) | IL1RN | IL1RN rs928940 | 0.000 | 1.639854 | 1.289854 | 2.084827 | 0 |
| 45 | Suh JS | 2013 | pediatric patients | Korea | 23659670 | IL22R1 | IL22R1 rs3795299 | 0.000 | 1.27992 | 1.121502 | 1.460714 | 0 |
| 22 | Hahn WH | 2010 | Korean pediatric patients | Korea | [19280228](https://www.ncbi.nlm.nih.gov/pubmed/23593433) | IL1RN | IL1RN rs439154 | 0.000 | 1.187571 | 1.03107 | 1.367827 | 0.017 |
| 22 | Hahn WH | 2010 | Korean pediatric patients | Korea | [19280228](https://www.ncbi.nlm.nih.gov/pubmed/23593433) | IL1RN | IL1RN rs315951 | 0.000 | 0.578987 | 0.353342 | 0.948731 | 0.03 |
| 9 | Zhang D | 2017 | Chinese | China | [29069743](https://www.ncbi.nlm.nih.gov/pubmed/23593433) | IL-6 | IL-6 rs1800796 | 0.000 | 0.780819 | 0.615499 | 0.990542 | 0.042 |
| 2 | Zhou XJ | 2013 | Chinese | china | [23593433](https://www.ncbi.nlm.nih.gov/pubmed/23593433) | intergenic | intergenic rs7549830 | 0.000 | 1.289657 | 1.006775 | 1.652024 | 0.044 |
| 2 | Zhou XJ | 2013 | Chinese | china | [23593433](https://www.ncbi.nlm.nih.gov/pubmed/23593433) | intergenic | intergenic rs10494356 | 0.930 | 3.220588 | 2.004834 | 5.173588 | 0 |
| 1 | Shi D | 2020 | Chinese | china | [31857673](https://www.ncbi.nlm.nih.gov/pubmed/23593433) | LEMD2 | LEMD2 rs751728 | 0.820 | 2.588944 | 1.596014 | 4.199605 | 0 |
| 2 | Zhou XJ | 2013 | Chinese | china | [23593433](https://www.ncbi.nlm.nih.gov/pubmed/23593433) | NA | NA rs6657266 | 0.990 | 3.701299 | 2.186932 | 6.264307 | 0 |
| 2 | Zhou XJ | 2013 | Chinese | china | [23593433](https://www.ncbi.nlm.nih.gov/pubmed/23593433) | NA | NA rs12745240 | 0.634 | 0.194286 | 0.04014 | 0.940374 | 0.042 |
| 2 | Zhou XJ | 2013 | Chinese | china | [23593433](https://www.ncbi.nlm.nih.gov/pubmed/23593433) | NA | NA rs1063178 | 0.259 | 0.457815 | 0.254568 | 0.823334 | 0.009 |
| 34 | Feng Y | 2019 | chinese | China | [30928649](https://www.ncbi.nlm.nih.gov/pubmed/23593433) | NTN4 | NTN4 rs1362970 | 0.448 | 0.554397 | 0.343279 | 0.895352 | 0.016 |
| 13 | Zhou XJ | 2016 | Chinese | China | [27804980](https://www.ncbi.nlm.nih.gov/pubmed/23593433) | PCNXL3 | PCNXL3 rs2009453 | 0.200 | 0.559048 | 0.336219 | 0.929558 | 0.025 |
| 13 | Zhou XJ | 2016 | Chinese | China | [27804980](https://www.ncbi.nlm.nih.gov/pubmed/23593433) | RASGRP1 | RASGRP1 rs7170151 | 0.170 | 1.675084 | 1.055394 | 2.658636 | 0.029 |
| 13 | Zhou XJ | 2016 | Chinese | China | [27804980](https://www.ncbi.nlm.nih.gov/pubmed/23593433) | RGS1 | RGS1 rs12022418 | 0.422 | 0.523603 | 0.291861 | 0.939354 | 0.03 |
| 16 | Zhou XJ | 2021 | Chinese | China | [33462083](https://www.ncbi.nlm.nih.gov/pubmed/23593433) | STAT3 | STAT3 rs744166 | 0.705 | 3.870968 | 1.645176 | 9.108077 | 0.002 |
| 16 | Zhou XJ | 2021 | Chinese | China | [33462083](https://www.ncbi.nlm.nih.gov/pubmed/23593433) | STAT3 | STAT3 rs744166 | 0.823 | 3.1261 | 1.342395 | 7.279898 | 0.008 |
| 17 | Yang B | 2018 | Chinese | China | [29467950](https://www.ncbi.nlm.nih.gov/pubmed/23593433) | STAT4 | STAT4 rs7574865 | 0.213 | 0.611801 | 0.46695 | 0.801587 | 0 |
| 42 | Vuong MT | 2009 | Sweden | Sweden | [19258388](https://www.ncbi.nlm.nih.gov/pubmed/23593433) | TGF‐β1 | TGF‐β1 rs6957 | 0.250 | 1.985393 | 1.094705 | 3.600776 | 0.024 |
| 42 | Vuong MT | 2009 | Sweden | Sweden | [19258388](https://www.ncbi.nlm.nih.gov/pubmed/23593433) | TGF‐β1 | TGF‐β1 rs180047 | 0.102 | 0.137168 | 0.036323 | 0.517999 | 0.003 |
| 6 | Lee JS | 2010 | Korean pediatric patients | Korea | [21108742](https://www.ncbi.nlm.nih.gov/pubmed/23593433) | TLR1 | TLR1 rs5743557 | 0.146 | 0.049315 | 0.002634 | 0.923182 | 0.044 |
| 7 | Park HJ | 2011 | Korean pediatric patients | Korea | [20953797](https://www.ncbi.nlm.nih.gov/pubmed/23593433) | TLR10 | TLR10 rs10004195 | 0.052 | 0.074526 | 0.009816 | 0.565837 | 0.012 |
| 33 | Zhong Z | 2017 | chinese | China | [28636766](https://www.ncbi.nlm.nih.gov/pubmed/23593433) | TNFSF13 | TNFSF13 rs3803800 | 0.052 | 0.155628 | 0.035314 | 0.685838 | 0.014 |
| 23 | Jung HY | 2012 | Korean | Korea | [26889427](https://www.ncbi.nlm.nih.gov/pubmed/23593433) | VEGF | VEGF 405C-G | 0.208 | 0.325752 | 0.130305 | 0.814354 | 0.016 |

**Supplementary Table6:**

|  | \| 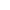author \| \| --- \| | year | ethnicity | country | PMID | SNP | PHWE | Heterozygote(OR) | 0.95_LCI | 0.95_UCI | P |
| --- | --- | --- | --- | --- | --- | --- | --- | --- | --- | --- | --- | --- |
| 1 | Shi D | 2020 | Chinese | China | [31857673](https://www.ncbi.nlm.nih.gov/pubmed/31857673) | CFB rs4151657 | 0.460 | 1.299812 | 1.14021 | 1.481754 | 0 |
| 1 | Shi D | 2020 | Chinese | China | [31857673](https://www.ncbi.nlm.nih.gov/pubmed/31857673) | CFB rs4151657 | 0.340 | 1.276475 | 1.092897 | 1.490888 | 0.002 |
| 1 | Shi D | 2020 | Chinese | China | [31857673](https://www.ncbi.nlm.nih.gov/pubmed/31857673) | CFB rs4151657 | 0.900 | 1.357275 | 1.063215 | 1.732664 | 0.014 |
| 1 | Shi D | 2020 | Chinese | China | [31857673](https://www.ncbi.nlm.nih.gov/pubmed/31857673) | CFB rs549182 | 0.140 | 1.249885 | 1.01952 | 1.532301 | 0.032 |
| 2 | Zhou XJ | 2013 | Chinese | China | [23593433](https://www.ncbi.nlm.nih.gov/pubmed/31857673) | NA rs6657266 | 0.000 | 0.0871056 | 0.0633466 | 0.1197757 | 0 |
| 2 | Zhou XJ | 2013 | Chinese | China | [23593433](https://www.ncbi.nlm.nih.gov/pubmed/31857673) | FCGR2B rs12118043 | 0.012 | 0.7007164 | 0.5596106 | 0.8774021 | 0.002 |
| 2 | Zhou XJ | 2013 | Chinese | China | [23593433](https://www.ncbi.nlm.nih.gov/pubmed/31857673) | FCGR2B rs12118043 | 0.011 | 0.7007164 | 0.5596106 | 0.8774021 | 0.002 |
| 2 | Zhou XJ | 2013 | Chinese | China | [23593433](https://www.ncbi.nlm.nih.gov/pubmed/31857673) | intergenic rs4657039 | 0.160 | 0.771569 | 0.643495 | 0.9251333 | 0.005 |
| 2 | Zhou XJ | 2013 | Chinese | China | [23593433](https://www.ncbi.nlm.nih.gov/pubmed/31857673) | intergenic rs10917750 | 0.808 | 0.7701089 | 0.6349335 | 0.9340627 | 0.008 |
| 2 | Zhou XJ | 2013 | Chinese | China | [23593433](https://www.ncbi.nlm.nih.gov/pubmed/31857673) | FCRLB rs4657093 | 0.960 | 0.7669771 | 0.629744 | 0.9341158 | 0.008 |
| 2 | Zhou XJ | 2013 | Chinese | China | [23593433](https://www.ncbi.nlm.nih.gov/pubmed/31857673) | FCRLB rs1891019 | 0.030 | 0.7681942 | 0.6196268 | 0.9523835 | 0.016 |
| 2 | Zhou XJ | 2013 | Chinese | China | [23593433](https://www.ncbi.nlm.nih.gov/pubmed/31857673) | FCRLB rs1417582 | 0.804 | 0.7974089 | 0.6576874 | 0.9668135 | 0.021 |
| 2 | Zhou XJ | 2013 | Chinese | China | [23593433](https://www.ncbi.nlm.nih.gov/pubmed/31857673) | NA rs2165090 | 0.030 | 0.7937518 | 0.6518442 | 0.966553 | 0.022 |
| 2 | Zhou XJ | 2013 | Chinese | China | [23593433](https://www.ncbi.nlm.nih.gov/pubmed/31857673) | FCRLB rs1891020 | 0.834 | 0.8004059 | 0.6602914 | 0.9702528 | 0.023 |
| 2 | Zhou XJ | 2013 | Chinese | China | [23593433](https://www.ncbi.nlm.nih.gov/pubmed/31857673) | NA rs12749327 | 0.517 | 1.42754 | 1.036835 | 1.965471 | 0.029 |
| 2 | Zhou XJ | 2013 | Chinese | China | [23593433](https://www.ncbi.nlm.nih.gov/pubmed/31857673) | FCRLA rs1954173 | 0.413 | 0.8110807 | 0.6694205 | 0.9827184 | 0.033 |
| 2 | Zhou XJ | 2013 | Chinese | China | [23593433](https://www.ncbi.nlm.nih.gov/pubmed/31857673) | intergenic rs1503813 | 0.320 | 0.8170646 | 0.67799 | 0.9846672 | 0.034 |
| 2 | Zhou XJ | 2013 | Chinese | China | [23593433](https://www.ncbi.nlm.nih.gov/pubmed/31857673) | NA rs10800309 | 0.548 | 0.8255099 | 0.6881396 | 0.9903029 | 0.039 |
| 2 | Zhou XJ | 2013 | Chinese | China | [23593433](https://www.ncbi.nlm.nih.gov/pubmed/31857673) | NA rs7539036 | 0.035 | 1.285152 | 1.003568 | 1.645742 | 0.047 |
| 2 | Zhou XJ | 2013 | Chinese | China | [23593433](https://www.ncbi.nlm.nih.gov/pubmed/31857673) | NA rs6696854 | 0.840 | 0.8325025 | 0.6939057 | 0.9987819 | 0.048 |
| 2 | Zhou XJ | 2013 | Chinese | China | 23593433 | NA rs905589 | 0.264 | 0.828642 | 0.6879557 | 0.9980985 | 0.048 |
| 6 | Lee JS | 2010 | Korean pediatric patients | Korea | [21108742](https://www.ncbi.nlm.nih.gov/pubmed/23593433) | TLR1 rs4833095 | 0.083 | 2.1261 | 1.218108 | 3.710918 | 0.008 |
| 7 | Park HJ | 2011 | Korean pediatric patients | Korea | [20953797](https://www.ncbi.nlm.nih.gov/pubmed/23593433) | TLR10 rs10004195 | 0.323 | 1.981982 | 1.253534 | 3.133743 | 0.003 |
| 8 | [Gao J](https://pubmed.ncbi.nlm.nih.gov/?sort=date&term=Gao+J&cauthor_id=28391282) | 2018 | Chinese | China | [28391282](https://www.ncbi.nlm.nih.gov/pubmed/23593433) | IFN-γ rs430561 | 0.250 | 0.5726316 | 0.3390155 | 0.9672329 | 0.037 |
| 10 | [Gao J](https://pubmed.ncbi.nlm.nih.gov/?sort=date&term=Gao+J&cauthor_id=28391282) | 2017 | Chinese | China | [28359052](https://www.ncbi.nlm.nih.gov/pubmed/23593433) | IL-10 rs1800872 | 0.123 | 1.501976 | 1.081055 | 2.086788 | 0.015 |
| 10 | [Gao J](https://pubmed.ncbi.nlm.nih.gov/?sort=date&term=Gao+J&cauthor_id=28391282) | 2017 | Chinese | China | [28359052](https://www.ncbi.nlm.nih.gov/pubmed/23593433) | IL-10 rs1800871 | 0.139 | 1.498693 | 1.07935 | 2.080958 | 0.016 |
| 12 | Kim HJ | 2011 | Korean pediatric patients | Korea | [21677403](https://www.ncbi.nlm.nih.gov/pubmed/23593433) | CD29 rs3181097 | 0.016 | 2.296875 | 1.103525 | 4.78071 | 0.026 |
| 12 | Kim HJ | 2011 | Korean pediatric patients | Korea | [21677403](https://www.ncbi.nlm.nih.gov/pubmed/23593433) | ICOS rs4404254 | 0.338 | 2.288265 | 1.101335 | 4.754375 | 0.027 |
| 12 | Kim HJ | 2011 | Korean pediatric patients | Korea | [21677403](https://www.ncbi.nlm.nih.gov/pubmed/23593433) | ICOS rs10183087 | 0.400 | 2.247299 | 1.097481 | 4.601768 | 0.027 |
| 12 | Kim HJ | 2011 | Korean pediatric patients | Korea | [21677403](https://www.ncbi.nlm.nih.gov/pubmed/23593433) | ICOS rs11571314 | 0.400 | 2.247299 | 1.097481 | 4.601768 | 0.027 |
| 12 | Kim HJ | 2011 | Korean pediatric patients | Korea | [21677403](https://www.ncbi.nlm.nih.gov/pubmed/23593433) | ICOS rs1559931 | 0.400 | 2.247299 | 1.097481 | 4.601768 | 0.027 |
| 12 | Kim HJ | 2011 | Korean pediatric patients | Korea | [21677403](https://www.ncbi.nlm.nih.gov/pubmed/23593433) | ICOS rs4270326 | 0.756 | 2.180769 | 1.03162 | 4.609988 | 0.041 |
| 12 | Kim HJ | 2011 | Korean pediatric patients | Korea | [21677403](https://www.ncbi.nlm.nih.gov/pubmed/23593433) | CTLA4 rs231779 | 0.683 | 1.937322 | 1.019518 | 3.681365 | 0.043 |
| 13 | Zhou XJ | 2016 | Chinese | China | [27804980](https://www.ncbi.nlm.nih.gov/pubmed/23593433) | RGS1 rs12022418 | 0.000 | 1.236073 | 1.005081 | 1.520151 | 0.045 |
| 14 | Suh JS | 2011 | Korean pediatric patients | Korea | [21214373](https://www.ncbi.nlm.nih.gov/pubmed/23593433) | CXCL8 rs2227543 | 0.790 | 1.741433 | 1.173249 | 2.584779 | 0.006 |
| 14 | Suh JS | 2011 | Korean pediatric patients | Korea | [21214373](https://www.ncbi.nlm.nih.gov/pubmed/23593433) | CXCL8 rs2227306 | 0.395 | 1.683763 | 1.134176 | 2.499664 | 0.01 |
| 16 | Zhou XJ | 2021 | Chinese | China | [33462083](https://www.ncbi.nlm.nih.gov/pubmed/23593433) | FBXL21 rs40986 | 0.000 | 0.7673813 | 0.6028736 | 0.9767787 | 0.031 |
| 16 | Zhou XJ | 2021 | Chinese | China | [33462083](https://www.ncbi.nlm.nih.gov/pubmed/23593433) | CCR6 rs3093023 | 0.000 | 1.273448 | 1.017657 | 1.593533 | 0.035 |
| 16 | Zhou XJ | 2021 | Chinese | China | [33462083](https://www.ncbi.nlm.nih.gov/pubmed/23593433) | CCR6 rs3093023 | 0.000 | 1.131393 | 1.002148 | 1.277306 | 0.046 |
| 17 | Yang B | 2018 | Chinese | China | [29467950](https://www.ncbi.nlm.nih.gov/pubmed/23593433) | HLA-DP rs3077 | 0.930 | 0.3074198 | 0.1820647 | 0.5190842 | 0 |
| 17 | Yang B | 2018 | Chinese | China | [29467950](https://www.ncbi.nlm.nih.gov/pubmed/23593433) | STAT4 rs7574865 | 0.820 | 0.5600479 | 0.3543432 | 0.8851691 | 0.013 |
| 18 | Jacob M | 2018 | Caucasians | Germany | [29539619](https://www.ncbi.nlm.nih.gov/pubmed/23593433) | STAT4 rs5742909 | 0.153 | 1.683391 | 1.034466 | 2.739391 | 0.036 |
| 21 | Li GS | 2007 | Chinese | China | [17228361](https://www.ncbi.nlm.nih.gov/pubmed/23593433) | C1GALT1 rs5882115 | 0.634 | 0.7100885 | 0.5316033 | 0.9485 | 0.02 |
| 22 | Hahn WH | 2010 | Korean pediatric patients | Korea | [19280228](https://www.ncbi.nlm.nih.gov/pubmed/23593433) | IL-1B rs1143633 | 0.712 | 0.6032078 | 0.4155427 | 0.8756251 | 0.008 |
| 33 | Zhong Z | 2017 | Chinese | China | [28636766](https://www.ncbi.nlm.nih.gov/pubmed/23593433) | TNFSF13 rs3803800 | 0.213 | 0.6811554 | 0.5261266 | 0.8818652 | 0.004 |
| 34 | Feng Y | 2019 | Chinese | China | [30928649](https://www.ncbi.nlm.nih.gov/pubmed/23593433) | TNS3 rs3750163 | 1.000 | 17.98621 | 4.272992 | 75.70892 | 0 |
| 35 | Shi D | 2020 | Chinese | China | [31227791](https://www.ncbi.nlm.nih.gov/pubmed/23593433) | ITGAM rs4597342 | 0.520 | 0.8011732 | 0.6650555 | 0.9651502 | 0.03 |
| 35 | Shi D | 2020 | Chinese | China | [31227791](https://www.ncbi.nlm.nih.gov/pubmed/23593433) | ITGAX rs11150614 | 0.984 | 0.8202085 | 0.6807781 | 0.9881958 | 0.037 |
| 35 | Shi D | 2020 | Chinese | China | [31227791](https://www.ncbi.nlm.nih.gov/pubmed/23593433) | ITGAX rs1140195 | 0.623 | 0.8210266 | 0.6804152 | 0.9906961 | 0.04 |
| 42 | Vuong MT | 2009 | Sweden | Sweden | [19258388](https://www.ncbi.nlm.nih.gov/pubmed/23593433) | TGF‐β1 rs2241715 | 0.645 | 1.763136 | 1.164292 | 2.66999 | 0.007 |
| 42 | Vuong MT | 2009 | Sweden | Sweden | 19258388 | TGF‐β1 rs6957 | 0.102 | 0.1647059 | 0.0423612 | 0.6403985 | 0.009 |
| 42 | Vuong MT | 2009 | Sweden | Sweden | 19258388 | TGF‐β1 rs180047 | 0.146 | 0.0446735 | 0.002295 | 0.869583 | 0.04 |

**Supplementary Table7:**

|  | \| 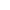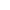author \| \| --- \| | year | ethnicity | country | PMID | SNP | PHWE | Recessive(OR) | 0.95_LCI | 0.95_UCI | P |
| --- | --- | --- | --- | --- | --- | --- | --- | --- | --- | --- | --- | --- |
| 44 | Suh JS | 2011 | pediatric patients | Korea | [22977507](https://www.ncbi.nlm.nih.gov/pubmed/31857673) | BMP2 rs1049007 | 0.052 | 0.1558029 | 0.035554 | 0.682756 | 0.014 |
| 44 | Suh JS | 2011 | pediatric patients | Korea | [22977507](https://www.ncbi.nlm.nih.gov/pubmed/31857673) | BMP2 rs235768 | 0.052 | 0.0728793 | 0.009642 | 0.550867 | 0.011 |
| 21 | Li GS | 2007 | Chinese | China | [17228361](https://www.ncbi.nlm.nih.gov/pubmed/31857673) | C1GALT1 rs1008898 | 0.065 | 1.352463 | 1.025251 | 1.784104 | 0.033 |
| 16 | Zhou XJ | 2021 | Chinese | China | [33462083](https://www.ncbi.nlm.nih.gov/pubmed/31857673) | CCR6 rs3093023(A/G) | 0.000 | 1.481797 | 1.190766 | 1.843959 | 0 |
| 16 | Zhou XJ | 2021 | Chinese | China | [33462083](https://www.ncbi.nlm.nih.gov/pubmed/31857673) | CCR6 rs3093023(A/G) | 0.000 | 1.214486 | 1.075702 | 1.371177 | 0.002 |
| 1 | Shi D | 2020 | Chinese | china | [31857673](https://www.ncbi.nlm.nih.gov/pubmed/31857673) | CFB rs549182 (A/G) | 0.140 | 3.896315 | 1.289986 | 11.76855 | 0.016 |
| 14 | Suh JS | 2011 | Korean pediatric patients | Korea | [21214373](https://www.ncbi.nlm.nih.gov/pubmed/31857673) | CXCL8 rs4073(A/T) | 0.911 | 0.6058537 | 0.413402 | 0.887899 | 0.01 |
| 16 | Zhou XJ | 2021 | Chinese | China | [33462083](https://www.ncbi.nlm.nih.gov/pubmed/31857673) | FBXL21 rs40986(C/T) | 0.000 | 0.6087347 | 0.372166 | 0.995679 | 0.048 |
| 2 | Zhou XJ | 2013 | Chinese | china | [23593433](https://www.ncbi.nlm.nih.gov/pubmed/31857673) | FCRLA rs1954174(T/C) | 0.434 | 1.313906 | 1.035815 | 1.666657 | 0.024 |
| 17 | Yang B | 2018 | Chinese | China | [29467950](https://www.ncbi.nlm.nih.gov/pubmed/31857673) | HLA-DP rs3077(G/A) | 0.930 | 4.932584 | 3.144951 | 7.736334 | 0 |
| 17 | Yang B | 2018 | Chinese | China | [29467950](https://www.ncbi.nlm.nih.gov/pubmed/31857673) | HLA-DP rs9277535(G/A) | 0.990 | 2.842105 | 1.838324 | 4.393982 | 0 |
| 38 | Liu XQ | 2008 | Caucasians | Canada | [18256355](https://www.ncbi.nlm.nih.gov/pubmed/31857673) | IGAN1 rs1342646(A/G) | 0.059 | 2.15873 | 1.429505 | 3.25995 | 0 |
| 38 | Liu XQ | 2008 | Caucasians | Canada | [18256355](https://www.ncbi.nlm.nih.gov/pubmed/31857673) | IGAN1 rs1203344(A/G) | 0.001 | 0.454023 | 0.259994 | 0.792853 | 0.006 |
| 38 | Liu XQ | 2008 | Caucasians | Canada | [18256355](https://www.ncbi.nlm.nih.gov/pubmed/31857673) | IGAN1 rs1203350(A/T) | 0.001 | 0.454023 | 0.258205 | 0.798345 | 0.006 |
| 9 | Zhang D | 2017 | Chinese | China | [29069743](https://www.ncbi.nlm.nih.gov/pubmed/31857673) | IL-1B rs16944(G/A) | 0.162 | 1.440016 | 1.039528 | 1.994796 | 0.028 |
| 22 | Hahn WH | 2010 | Korean pediatric patients | Korea | [19280228](https://www.ncbi.nlm.nih.gov/pubmed/31857673) | IL-1B rs3917356(G/A) | 0.170 | 1.575564 | 1.072024 | 2.315621 | 0.021 |
| 23 | Jung HY | 2012 | Korean | Korea | [26889427](https://www.ncbi.nlm.nih.gov/pubmed/31857673) | IL-1B rs1946518(C/A) | 0.705 | 2.853598 | 1.535734 | 5.302364 | 0.001 |
| 24 | Yang B | 2017 | Chinese Han population | China | [27028244](https://www.ncbi.nlm.nih.gov/pubmed/31857673) | IL-1B rs1946518(C/A) | 0.390 | 1.885001 | 1.183359 | 3.002663 | 0.008 |
| 22 | Hahn WH | 2010 | Korean pediatric patients | Korea | [19280228](https://www.ncbi.nlm.nih.gov/pubmed/31857673) | IL1RN rs928940(G/T) | 0.200 | 0.6221452 | 0.393763 | 0.982989 | 0.042 |
| 44 | Suh JS | 2013 | pediatric patients | Korea | [23659670](https://www.ncbi.nlm.nih.gov/pubmed/31857673) | IL22R1 rs3795299(C/G) | 0.208 | 0.2839325 | 0.115565 | 0.697597 | 0.006 |
| 22 | Hahn WH | 2010 | Korean pediatric patients | Korea | 19280228 | IL1RN rs315951(C/G) | 0.259 | 0.4652855 | 0.268381 | 0.806655 | 0.006 |
| 38 | Liu XQ | 2008 | Caucasians | Canada | [18256355](https://www.ncbi.nlm.nih.gov/pubmed/23593433) | IL5RA rs340833(A/G) | 0.918 | 2.528808 | 1.650067 | 3.875523 | 0 |
| 9 | Zhang D | 2017 | Chinese | China | [29069743](https://www.ncbi.nlm.nih.gov/pubmed/23593433) | IL-6 rs1800796(C/G) | 0.309 | 1.678732 | 1.072331 | 2.628051 | 0.023 |
| 2 | Zhou XJ | 2013 | Chinese | china | [23593433](https://www.ncbi.nlm.nih.gov/pubmed/23593433) | intergenic rs7549830(C/T) | 0.054 | 1.297275 | 1.052893 | 1.59838 | 0.015 |
| 35 | Shi D | 2020 | chinese | China | [31227791](https://www.ncbi.nlm.nih.gov/pubmed/23593433) | ITGAX rs11150619(C/T) | 0.152 | 7.369979 | 3.902877 | 13.91706 | 0 |
| 1 | Shi D | 2020 | Chinese | china | [31857673](https://www.ncbi.nlm.nih.gov/pubmed/23593433) | LEMD2 rs751728(A/G) | 0.570 | 0.6895846 | 0.47938 | 0.991963 | 0.045 |
| 2 | Zhou XJ | 2013 | Chinese | china | [23593433](https://www.ncbi.nlm.nih.gov/pubmed/23593433) | NA rs1063178(T/C) | 0.185 | 1.286375 | 1.03512 | 1.598617 | 0.023 |
| 2 | Zhou XJ | 2013 | Chinese | china | [23593433](https://www.ncbi.nlm.nih.gov/pubmed/23593433) | NA rs12745240(A/G) | 0.148 | 1.286375 | 1.03512 | 1.598617 | 0.023 |
| 2 | Zhou XJ | 2013 | Chinese | china | [23593433](https://www.ncbi.nlm.nih.gov/pubmed/23593433) | NA rs6657266(T/C) | 0.000 | 0.3881244 | 0.254101 | 0.592837 | 0 |
| 34 | Feng Y | 2019 | chinese | China | [30928649](https://www.ncbi.nlm.nih.gov/pubmed/23593433) | NTN4 rs1362970(C/A) | 0.250 | 1.820635 | 1.012567 | 3.273572 | 0.045 |
| 13 | Zhou XJ | 2016 | Chinese | China | [27804980](https://www.ncbi.nlm.nih.gov/pubmed/23593433) | RASGRP1 rs7170151(/C) | 0.000 | 1.348882 | 1.101929 | 1.65118 | 0.004 |
| 13 | Zhou XJ | 2016 | Chinese | China | [27804980](https://www.ncbi.nlm.nih.gov/pubmed/23593433) | RGS1 rs12022418(/C) | 0.000 | 1.426622 | 1.105559 | 1.840922 | 0.006 |
| 37 | Fu D | 2020 | chinese | China | [32747022](https://www.ncbi.nlm.nih.gov/pubmed/23593433) | ST6GAL1 rs7634389(T/C) | 0.083 | 1.286982 | 1.015798 | 1.630563 | 0.037 |
| 37 | Fu D | 2020 | chinese | China | [32747022](https://www.ncbi.nlm.nih.gov/pubmed/23593433) | ST6GAL1 rs2284750(C/T) | 0.377 | 0.6357138 | 0.489796 | 0.825102 | 0.001 |
| 37 | Fu D | 2020 | chinese | China | [32747022](https://www.ncbi.nlm.nih.gov/pubmed/23593433) | ST6GAL1 rs4686838(G/A) | 0.379 | 0.4800525 | 0.383384 | 0.601097 | 0 |
| 16 | Zhou XJ | 2021 | Chinese | China | [33462083](https://www.ncbi.nlm.nih.gov/pubmed/23593433) | STAT3 rs744166(G/A) | 0.000 | 1.150375 | 1.005893 | 1.31561 | 0.041 |
| 17 | Yang B | 2018 | Chinese | China | [29467950](https://www.ncbi.nlm.nih.gov/pubmed/23593433) | STAT4 rs7574865(G/T) | 0.820 | 3.301813 | 2.101682 | 5.187258 | 0 |
| 23 | Jung HY | 2012 | Korean | Korea | [26889427](https://www.ncbi.nlm.nih.gov/pubmed/23593433) | TGF‐β1 rs1982073(T/C) | 0.099 | 1.159375 | 0.618292 | 2.173975 | 0.025 |
| 41 | Lim CS | 2005 | korean | Korea | [15730046](https://www.ncbi.nlm.nih.gov/pubmed/23593433) | TGF‐β1 rs1800469(C/T) | 0.340 | 2.175824 | 1.01478 | 4.665257 | 0.046 |
| 42 | Vuong MT | 2009 | Sweden | Sweden | [19258388](https://www.ncbi.nlm.nih.gov/pubmed/23593433) | TGF‐β1 rs1800469(C/T) | 0.660 | 0.5234633 | 0.349893 | 0.783137 | 0.002 |
| 38 | Liu XQ | 2008 | Caucasians | Canada | [18256355](https://www.ncbi.nlm.nih.gov/pubmed/23593433) | TNFRSF6B rs3208008(A/C) | 0.881 | 0.2228774 | 0.080258 | 0.618932 | 0.004 |
| 38 | Liu XQ | 2008 | Caucasians | Canada | [18256355](https://www.ncbi.nlm.nih.gov/pubmed/23593433) | TNFRSF6B rs1291205(C/G) | 0.965 | 0.2220395 | 0.079959 | 0.616589 | 0.004 |
| 38 | Liu XQ | 2008 | Caucasians | Canada | [18256355](https://www.ncbi.nlm.nih.gov/pubmed/23593433) | TNFRSF6B rs1291206(A/G) | 0.958 | 0.2220395 | 0.079959 | 0.616589 | 0.004 |
| 33 | Zhong Z | 2017 | chinese | China | [28636766](https://www.ncbi.nlm.nih.gov/pubmed/23593433) | TNFSF13 rs3803800(A/G) | 0.213 | 0.8157251 | 0.676316 | 0.983871 | 0.033 |
| 23 | Jung HY | 2012 | Korean | Korea | [26889427](https://www.ncbi.nlm.nih.gov/pubmed/23593433) | VEGF 405C-G | 0.823 | 2.243061 | 1.197006 | 4.203256 | 0.012 |
