# Supplementary material for: Association of Immune and Inflammatory Gene Polymorphism With the Risk of IgA Nephropathy: A Systematic Review and Meta-Analysis of 45 Studies
Source: Front Immunol. 2021 Jun 30;12:683913. doi: 10.3389/fimmu.2021.683913 (PMC8329849; doi:10.3389/fimmu.2021.683913)
Supplement: Supplementary file 14 [file Table_2.docx]

|  | **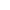**   \| **Study** \| \| --- \| | **Adequate definition of cases** | **Representativeness of the Cases** | **Selection of controls** | **Definition of controls** | **Control for important factor** | **Ascertainment of exposure** | **Same method of ascertainment for cases and controls** | **Non-response rate** | **Score** |
| --- | --- | --- | --- | --- | --- | --- | --- | --- | --- | --- | --- |
| 1 | Shi D (1) | * | * | / | * | ** | * | * | * | 8 |
| 2 | Zhou XJ (2) | * | * | / | / | * | * | * | * | 6 |
| 3 | Wang H (3) | * | * | / | / | ** | * | * | * | 7 |
| 4 | Szelestei T (4) | * | * | / | / | / | * | * | * | 5 |
| 5 | Steinmetz OM (5) | * | * | * | / | ** | * | * | * | 8 |
| 6 | Lee JS (6) | * | * | * | / | * | * | * | * | 7 |
| 7 | Park HJ (7) | * | * | * | * | * | * | * | * | 8 |
| 8 | Gao J(8) | * | * | / | / | ** | * | * | * | 7 |
| 9 | Zhang D (9) | * | * | * | * | * | * | * | * | 8 |
| 10 | Gao J (10) | * | * | / | * | * | * | * | * | 7 |
| 11 | Wu C (11) | * | * | * | * | * | * | * | * | 8 |
| 12 | Kim HJ (12) | * | * | * | * | * | * | * | * | 8 |
| 13 | Zhou XJ (13) | * | * | / | / | ** | * | * | * | 7 |
| 14 | Suh JS (14) | * | * | / | * | / | * | * | * | 6 |
| 15 | Cheng W (15) | * | * | / | / | * | * | * | * | 6 |
| 16 | Zhou XJ (16) | * | * | * | * | ** | * | * | * | 9 |
| 17 | Yang B (17) | * | * | / | * | ** | * | * | * | 8 |
| 18 | Jacob M (18) | * | * | * | / | * | * | * | * | 7 |
| 19 | Wolf G (19) | * | * | * | / | / | * | * | * | 6 |
| 20 | Wei L (20) | * | * | / | * | ** | * | * | * | 8 |
| 21 | Li GS (21) | * | * | / | * | * | * | * | * | 7 |
| 22 | Hahn WH (22) | * | * | / | * | / | * | * | * | 6 |
| 23 | Jung HY (23) | * | * | / | * | / | * | * | * | 6 |
| 24 | Yang B (24) | * | * | / | * | ** | * | * | * | 8 |
| 25 | Mao J (25) | * | * | / | / | * | * | * | * | 6 |
| 26 | Gao J (26) | * | * | / | / | ** | * | * | * | 7 |
| 27 | Wei LT (27) | * | * | / | / | ** | * | * | * | 7 |
| 28 | Gao J (28) | * | * | / | * | ** | * | * | * | 8 |
| 29 | Gao J (29) | * | * | / | * | ** | * | * | * | 8 |
| 30 | Xia YF (30) | * | * | * | / | * | * | * | * | 7 |
| 31 | Lim CS (31) | * | * | / | * | * | * | * | * | 7 |
| 32 | Hahn WH (32) | * | * | / | * | / | * | * | * | 6 |
| 33 | Zhong Z (33) | * | * | * | * | ** | * | * | * | 9 |
| 34 | Feng Y (34) | * | * | / | * | * | * | * | * | 7 |
| 35 | Shi D (35) | * | * | / | * | ** | * | * | * | 8 |
| 36 | Lu C (36) | * | * | / | / | * | * | * | * | 6 |
| 37 | Fu D (37) | * | * | / | / | * | * | * | * | 6 |
| 38 | Liu XQ (38) | * | * | / | / | * | * | * | * | 6 |
| 39 | Sato F (39) | * | * | * | * | / | * | * | * | 7 |
| 40 | Carturan S (40) | * | * | * | / | * | * | * | * | 6 |
| 41 | Lim CS (41) | * | * | / | * | * | * | * | * | 7 |
| 42 | Vuong MT (42) | * | * | / | / | ** | * | * | * | 7 |
| 43 | Brezzi B (43) | * | * | * | * | ** | * | * | * | 9 |
| 44 | Suh JS (44) | * | * | / | * | / | * | * | * | 6 |
| 45 | Suh JS (45) | * | * | / | * | / | * | * | * | 6 |
